# Chronic obstructive pulmonary disease and risk of incident cardiovascular disease, chronic kidney disease, and death: a systematic review and meta-analysis

**DOI:** 10.1016/j.eclinm.2026.104131

**Published:** 2026-08-07

**Authors:** Tobin Joseph, Chenyi Gao, Ramesh Nadarajah, Riyad Al-Lehebi, Luis Alves, David D. Berg, Amy Couper, Indranil Datta, Loay Eleyan, Mohammad Haris, Nathanial Hawkins, John R. Hurst, Christine R. Jenkins, Alan Kaplan, Janwillem W.H. Kocks, Konstantinos Kostikas, Carolyn SP. Lam, Therese Laperre, Fernando Martinez, Alexandra Mircescu, Maria Felicia Montero Arias, Umbreen Nadeem, Long Nguyen, Kate Wignall, Rachel Pullen, Keerthenan Raveendra, Chin Kook Rhee, Naveed Sattar, Daiana Stolz, Yongchang Sun, Gary Tse, Tonya Winders, David B. Price, Mohit Bhutani, Jianhua Wu, Chris P. Gale

**Affiliations:** a Leeds Institute of Cardiovascular and Metabolic Medicine, University of Leeds, Leeds, UK; b Leeds Institute for Data Analytics, University of Leeds, Leeds, UK; c Department of Cardiology, Leeds Teaching Hospitals NHS Trust, Leeds, UK; d Wolfson Institute of Population Health, Queen Mary University of London, London, UK; e Department of Pulmonology, King Fahad Medical City, Riyadh, Saudi Arabia College of Medicine, Alfaisal University, Riyadh, Saudi Arabia; f EPI Unit, Institute of Public Health, University of Porto, Porto, Portugal; g Division of Cardiovascular Medicine, Brigham and Women’s Hospital, Harvard Medical School, Boston, MA, USA; h Optimum Patient Care Global, Cambridge, UK; i Observational and Pragmatic Research Institute, Singapore, Republic of Singapore; j Division of Cardiology, Centre for Cardiovascular Innovation, University of British Columbia, Vancouver, British Columbia, Canada; k UCL Respiratory, University College London, London, UK; l Head Respiratory Group, The George Institute, Randwick, New South Wales, Australia; m Family Physician Airways Group of Canada, Stouffville, Ontario, Canada; n Department of Family and Community Medicine, University of Toronto, Toronto, Canada; o General Practitioners Research Institute, Groningen, the Netherlands; p Groningen Research Institute Asthma and COPD (GRIAC), University of Groningen, University Medical Center Groningen, Groningen, the Netherlands; q Dept of Pulmonology, University of Groningen, University Medical Center Groningen, Groningen, the Netherlands; r Respiratory Medicine Department, University of Ioannina, Ioannina, Greece; s National Heart Centre Singapore in Singapore, Duke-National University of Singapore (C.S.P.L), Singapore; t Translational Research in Immunology and Inflammation, Laboratory of Experimental Medicine and Pediatrics, University of Antwerp, Antwerp, Belgium; u Department of Pneumology, Antwerp University Hospital, Edegem, Belgium; v University of Massachusetts Chan Medical School/UMassMemorial Health, Worcester, MA, USA; w Department of Respiratory Medicine, Hospital México, San José, 10107, Costa Rica; x Department of Pulmonology, Hospital Clínica Bíblica, Calle Central Alfredo Volio, San José, 10101, Costa Rica; y Observational and Pragmatic Research Institute, Cambridge, UK; z Division of Pulmonary, Allergy and Critical Care Medicine, Department of Internal Medicine, Konkuk University Medical Center, Konkuk University School of Medicine, Seoul, Republic of Korea; aa School of Cardiovascular and Metabolic Health, University of Glasgow, Glasgow, UK; ab Clinic of Respiratory Medicine and Faculty of Medicine, University of Freiburg, Freiburg, Germany; ac Department of Respiratory and Critical Care Medicine, Peking University Third Hospital, Beijing, China; ad School of Nursing and Health Sciences, Hong Kong Metropolitan University, Hong Kong SAR, China; ae Tianjin Key Laboratory of Ionic-Molecular Function of Cardiovascular Disease, Department of Cardiology, Tianjin Institute of Cardiology, Second Hospital of Tianjin Medical University, Tianjin, China; af Global Allergy & Airways Patient Platform, Vienna, Austria; ag Division of Applied Health Sciences, Centre of Academic Primary Care, University of Aberdeen, Aberdeen, UK; ah Department of Medicine, University of Alberta, Edmonton, Alberta, Canada; ai Laboratory for Integrative and Translational Research in Population Health (ITR), Porto, Portugal

**Keywords:** Chronic obstructive pulmonary disease, Cardiovascular disease, Chronic kidney disease, Mortality, Meta-analysis, Systematic review

## Abstract

**Background:**

Chronic obstructive pulmonary disease (COPD) is associated with cardiovascular disease and chronic kidney disease, but with conflicting estimates. We aimed to quantify the association of COPD and incident cardiovascular diseases, chronic kidney disease and death.

**Methods:**

In this systematic review and meta-analysis, we searched MEDLINE and Embase for case-control studies reporting associations between COPD and cardiovascular diseases, chronic kidney disease and death from database inception until 15 April 2026. Two reviewers independently extracted study characteristics and reported risk ratios of incident outcomes associated with COPD, specifically: atrial fibrillation and flutter, ventricular fibrillation and tachycardia, myocardial infarction, ischaemic stroke, heart failure, peripheral arterial disease, chronic kidney disease, cardiovascular mortality and all-cause mortality. Pooled estimates were obtained using random-effects models with restricted maximum likelihood estimation as substantial heterogeneity was anticipated. Ten sensitivity analyses stratifying studies by design, follow-up duration, region, clinical context, sample size, publication date, COPD ascertainment, leave-one-out, ratio adjustment and risk of bias, subgroup analyses by age and risk group were undertaken, and subsequent univariate and multivariate meta regression was performed. Study quality was assessed using the risk of bias in non-randomised follow-up studies of exposure effects (ROBINS-E) tool, and certainty of evidence was assessed using Grading of Assessment, Evaluation, Development and Evaluation (GRADE) criteria. Risk of publication bias was assessed using funnel plots and Egger’s regression. This review was registered on PROSPERO (CRD42025639084).

**Findings:**

Of 140 case-control studies including 30,144,481 patients (3,062,712 with COPD) with median follow -up duration of 3.0 years (IQR 1.0–5.2), COPD was associated with increased risk of heart failure (risk ratio [RR] 2.33, 95% CI 1.78–3.06), ventricular tachycardia (2.05, 1.27–3.31), peripheral arterial disease (1.99, 1.49–2.65), heart failure hospitalisation (1.78, 1.28–2.47), chronic kidney disease (1.65, 1.25–2.16), all-cause mortality (1.55, 1.42–1.70), cardiovascular mortality (1.55, 1.33–1.82), myocardial infarction (1.47, 1.28–1.68), ischaemic stroke (1.38, 1.18–1.62), and atrial fibrillation (1.38, 1.19–1.61) when compared to those without COPD. Despite substantial heterogeneity for all outcomes, associations between COPD and outcomes were broadly consistent across sensitivity analyses.

**Interpretation:**

COPD is associated with an increased risk for a range of incident cardiovascular diseases, chronic kidney disease and mortality. The impact of current and novel treatments on a broader range of cardiovascular and kidney outcomes in patients with COPD requires prospective randomised assessment.

**Funding:**

The British Heart Foundation.

**Research in context**

**Evidence before this study**

We conducted an extensive search in MEDINE and Embase from inception to 15th July 2024 with no language restrictions, for meta-analyses investigating cardio-kidney associations with COPD. Terms used included “COPD,” “cardiovascular,” “mortality,” “kidney” alongside disease specific terms. One study including 28 cohorts pooled risk of comorbid cardiovascular disease (ischaemic heart disease, dysrhythmia, heart failure, arterial disease and disorders of the pulmonary circulation; odds ratio [OR] 2.46; 95% confidence interval [CI] 2.02–3.00; p < 0.0001). However, this study demonstrates associations with prevalent disease and does not quantify risk of developing incident disease. Risk estimates of chronic kidney disease and both cardiovascular and all-cause mortality for those with COPD compared to those without are conflicting.

**Added value of this study**

Our study provides contemporaneous and comprehensive risk estimates for the association of COPD and a range of specific cardiovascular diseases, chronic kidney disease, and cardiovascular and all-cause mortality. Involving data from over 30 million patients it is the largest comprehensive meta-analysis to date both in terms of patient number and range of investigated outcomes. Patients with COPD have double the risk than the general population of developing incident heart failure, ventricular tachycardia or peripheral arterial disease. COPD is also associated with an increased risk of a range of cardiovascular-kidney outcomes including myocardial infarction, ischaemic stroke, atrial fibrillation, chronic kidney disease, heart failure hospitalisation and both cardiovascular and all-cause mortality. Furthermore, increased risk was robust across ten sensitivity analyses.

**Implications of all the available evidence**

COPD is associated with increased risk of cardiovascular disease, chronic kidney disease, and both cardiovascular and all-cause mortality. Interventions are needed to reduce the risk of cardiovascular and kidney outcomes in adults with COPD.

## Introduction

Chronic obstructive pulmonary disease (COPD) affects an estimated 480 million people, is one of the top three causes of death, and is projected to rapidly increase in prevalence.^1^^,^^2^ Current guidelines and clinical care focus on the reduction of respiratory outcomes – namely the prevention of respiratory failure, moderate to severe COPD exacerbations and improving symptoms. However, studies show that COPD is associated with adverse cardiovascular and kidney events.^3^, ^4^, ^5^, ^6^, ^7^

Accordingly, it is now proposed that cardiopulmonary risk,^3^^,^^7^^,^^8^ namely the risk of developing a moderate-to-severe exacerbation of COPD, myocardial infarction, stroke, heart failure, heart failure hospitalisation, atrial flutter or fibrillation, ventricular tachycardia or fibrillation or death due to any of these^7^ describes important outcomes to which treatments for COPD may be targeted, extending beyond a narrow emphasis on respiratory sequalae.

Previous investigations of the risk of cardiovascular and kidney diseases in COPD^6^ have offered conflicting results.^4^^,^^5^^,^^9^ In part, this is due to small sample sizes and heterogeneity amongst samples leading to non-significant or imprecise risk estimates. Pooling all available evidence may allow the determination of robust estimates of associations to facilitate public health efforts to reduce cardiovascular-kidney events in patients with COPD and inform outcome measure selection for randomised clinical trials.

Therefore, we conducted a systematic review and meta-analysis of associations between COPD and incident cardiovascular disease, chronic kidney disease and death, aiming to provide a comprehensive and contemporaneous set of relative risk estimates.

## Methods

### Search strategy and selection criteria

We searched MEDLINE and Embase databases through the Ovid platform from inception to April 15, 2026 (Supplementary Tables S1 and S2). We used a combination of keywords and subject headings related to cardiovascular events, chronic kidney disease and mortality in COPD, with records further limited to human studies and no language restrictions. We conducted forward and backward citation searching from included studies and previous systematic reviews. The grey literature was searched. We removed duplicates using Endnote’s duplicate identification strategy and by manual exclusion.

For inclusion, studies had to investigate the risk of one of the cardiovascular components for cardiopulmonary risk^7^ such as heart failure and heart failure hospitalisation, myocardial infarction, ischaemic stroke, cardiovascular death or arrhythmia (defined as atrial fibrillation, atrial flutter, ventricular tachycardia and ventricular fibrillation), or chronic kidney disease, peripheral arterial disease, or all-cause mortality (study definitions in Supplementary Table S3). Studies had to be a case control design, but we did not stipulate the minimum number of cases or duration of follow-up. Studies could be prospective or retrospective. We excluded case reports, letters, editorials, randomised controlled trials, case series and reviews. Systematic reviews were excluded, but references were searched for further potential studies to be included. We excluded sub-analyses or sequential analyses of initial study reports.

We uploaded records to a systematic review web application (Rayyan, Qatar Computing Research Institute). Two of TJ, KR, MH, UN, LE, AM, LN and ID independently screened titles, abstracts and full texts, with discrepancies settled by discussion and involvement of a third reviewer when required.

This review was registered on PROSPERO (CRD42025639084).

### Data analysis

Five investigators (AM, LN, LE, UE, ID) independently extracted the data from the studies with data checked by another investigator (TJ). All data were drawn from the primary reference and then assessed for risk of bias using the risk of bias in non-randomised follow-up studies of exposure effects (ROBINS-E) tool.^10^ The same five investigators also provided their risk of bias rating, that were checked by another investigator (TJ).

We extracted information on study characteristics (study name, investigator’s name, recruitment period, median duration of follow-up, year of publication of the primary findings), number of participants with and without COPD, mean age, percentage of men and risk of outcomes. We extracted raw event numbers, incidence rates, risk estimates and their 95% confidence intervals (CIs) for the association between COPD and all-cause mortality, cardiovascular mortality and disease specific events: myocardial infarction, ischaemic stroke, atrial fibrillation, atrial flutter, ventricular tachycardia, ventricular fibrillation, incident development of heart failure, chronic kidney disease, peripheral arterial disease and heart failure hospitalisation.

Where studies reported multiple effect estimates for the same outcome, we applied a pre-specified hierarchy: 1) preference was given to the estimate with the longest duration of follow-up; 2) if overlapping cohorts were reported, the largest cohort was selected; 3) for non-overlapping cohorts, all estimates were retained; and then 4) estimates for distinct outcomes were all included. To avoid double counting from multiple studies investigating the same outcome in the same region (and possible sample overlap), we applied the following hierarchy: 1) reviewed initial centre/location of the investigation 2) if similar location, review inclusion years to ensure no inclusion time overlap 3) if these could not be satisfactorily met, the largest study was included and the remaining excluded.

Certainty of evidence was assessed using the Grading of Assessment, Evaluation, Development and Evaluation (GRADE) criteria, encompassing five domains: risk of bias, inconsistency, indirectness, imprecision, and publication bias, with eventual gradings per outcome graded as high, moderate, low, or very low.^11^

We performed meta-analyses to synthesise observational data for binary outcomes, including only those reported in at least two studies with ≥10 participants and reported number of events in both COPD and non-COPD cohorts. Hazard ratios and odds ratios, if no other estimate were available, were converted to approximate relative risks (RRs) to allow consistent pooling, as described previously.^12^ Where only raw event counts were available, crude RRs were calculated to further facilitate effective pooling. Studies that did not report sufficient event data were excluded.

Pooled estimates were obtained using random-effects models with restricted maximum likelihood estimation, as moderate to high heterogeneity across study populations was anticipated. Between-study heterogeneity was quantified with the I^2^ statistic and Cochran’s Q test, with I^2^ > 40% or Q p < 0.10 considered evidence of substantial heterogeneity. When ≥10 studies were available, small-study effects were explored by visual inspection of funnel plots and formally tested using Egger’s regression.

Relative risks with 95% confidence intervals were the primary summary measure. To improve clinical interpretation, we also estimated absolute risk differences (ARDs) between COPD and control groups. For each outcome, the synthesised baseline absolute event rate in the control group was multiplied by (RR − 1), yielding the corresponding ARD.

Ten sensitivity analyses were conducted, stratifying studies by: design (prospective versus retrospective); follow-up duration (<6 versus ≥6 months); region (Asia, Europe, North America, multiple regions); clinical context (community, inpatient admission, peri-procedural (including specific procedure), intensive care admission); sample size (excluding the lowest tertile); publication date (before versus after 31st December 2010); risk of bias (high versus low/moderate, assessed by ROBINS-E); COPD ascertainment (clinically diagnosed, code defined and spirometry); crude versus adjusted ratio and leave-one out analyses. Subgroup analyses by age (<75 versus ≥75 years) and clinical risk were undertaken. Community dwelling individuals, regardless of their other comorbidities or inclusion criteria for the study, were deemed low risk. This included data from general population, prospective registries. High risk included those either admitted for critical illness, acute coronary syndrome or undergoing an invasive procedure (e.g. coronary artery bypass grafting, transcatheter aortic valve implantation, major non cardiac surgery, intensive care admission). Analyses were not attempted for outcomes supported by fewer than two studies.

To further explore the source of heterogeneity, we performed meta-regression analysis considering age (mean), sex (male%), risk of bias, smoking (%), diabetes (%), hypertension (%), and COPD ascertainment method.

All tests were two-sided, with statistical significance defined at p < 0.05. Where studies reported multiple cohorts, each was analysed independently. All analyses were conducted using R version 4.4.1 with the *metafor* package.

### Ethics

Ethical approval was not sought for this study.

### Role of funding source

The funder did not influence the study design, data collection, analysis, interpretation of data, manuscript preparation, or the decision to submit the paper for publication. The authors retained full access to all study data and held ultimate responsibility for the decision to submit the manuscript for publication.

## Results

**Fig. 1:**
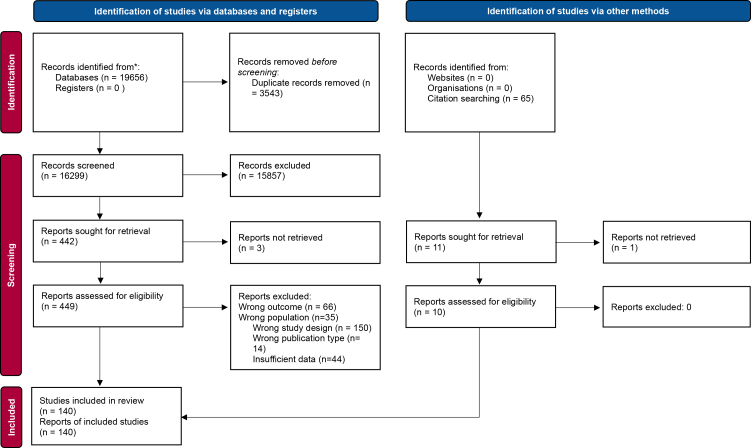
**Preferred reporting items for systematic reviews and meta-analyses (PRISMA) flow diagram for study selection**.

We reviewed 16,299 studies and excluded 15,857 studies following title and abstract screening. After reviewing 442 full text articles we excluded 309 studies, and after citation searching we included 10 additional studies. At extraction, 44 studies were excluded due to insufficient event data (Supplementary Table S4) to leave a final number of 140 studies that included a total of 3,062,712 patients with COPD and 27,072,352 controls (total n = 30,144,481; Fig. 1). The median duration of follow-up across the studies was 3.0 years (IQR 1.0–5.2).

According to the ROBINS-E tool, 54 (38.6%) of 140 studies were judged to be at high risk of bias. Detailed study characteristics, risk of bias assessments, and outcome definitions used are included in the Supplementary Tables S4–S7 and Supplementary Figs. S1 and S2. Outcome measure definitions were broadly similar, however, the criteria in larger prospective studies and secondary analysis of trials were more detailed. Funnel plots showed no evidence of publication bias for any outcome (Supplementary Figs. S2–S13; Egger’s test p > 0.05).

**Fig. 2:**
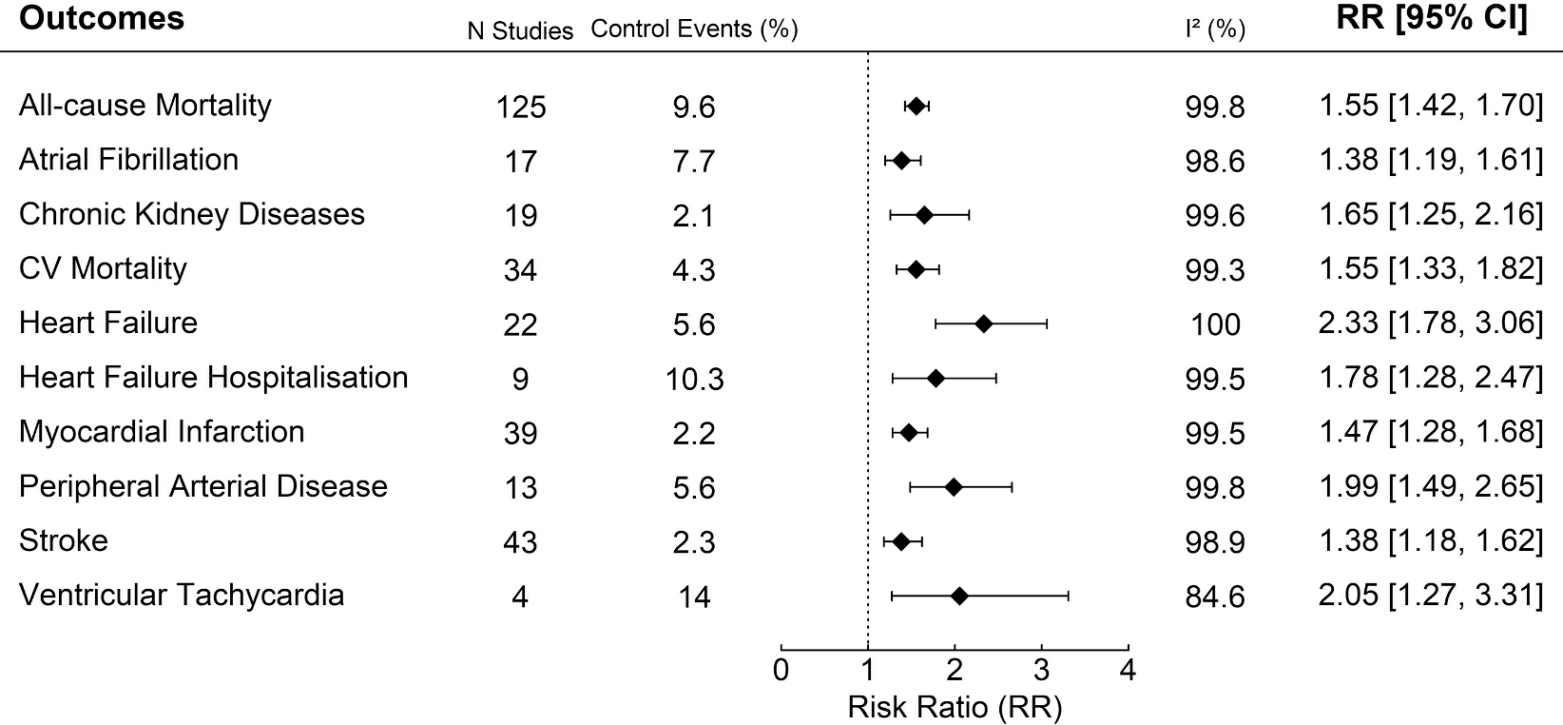
**Pooled relative risks for all outcomes.** CV—cardiovascular; CI—confidence interval; I^2^ denotes the heterogeneity statistic.; RR—risk ratio.

**Fig. 3:**
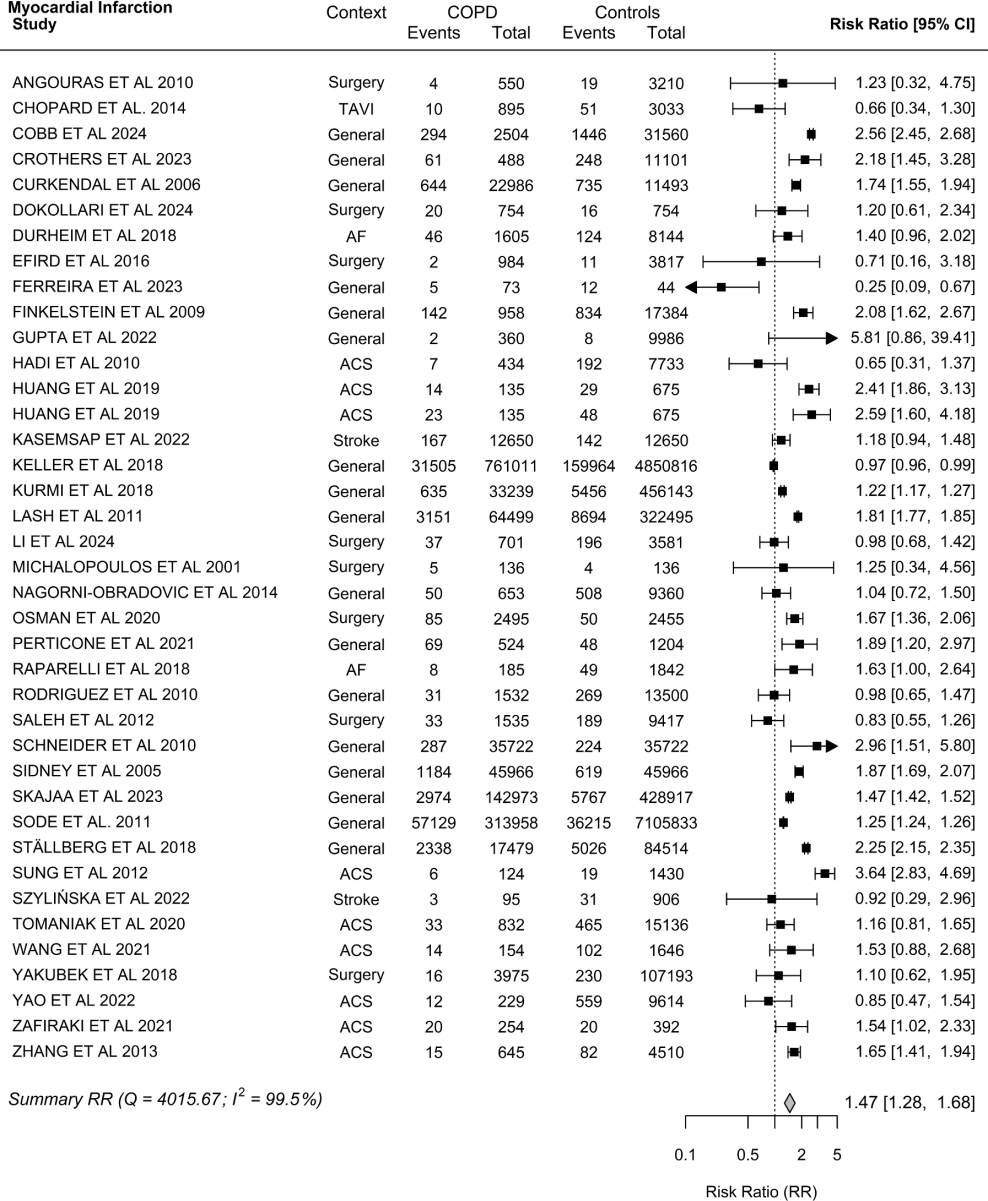
**Association between COPD and myocardial infarction.** ACS—acute coronary syndrome; AF—atrial fibrillation; COPD—chronic obstructive pulmonary disease; CI—confidence interval; I^2^ denotes the heterogeneity statistic.; RR—risk ratio; TAVI—transcatheter aortic valve implantation.

38 studies^13^, ^14^, ^15^, ^16^, ^17^, ^18^, ^19^, ^20^, ^21^, ^22^, ^23^, ^24^, ^25^, ^26^, ^27^, ^28^, ^29^, ^30^, ^31^, ^32^, ^33^, ^34^, ^35^, ^36^, ^37^, ^38^, ^39^, ^40^, ^41^, ^42^, ^43^, ^44^, ^45^, ^46^, ^47^, ^48^, ^49^, ^50^ including 13,634,312 patients (1,472,505 [10.8%] with COPD) reported myocardial infarction as an outcome with a median follow-up 3.0 years. The myocardial infarction event rate in the control group was 2.22% (95% CI 1.59–3.09%). The pooled relative risk was 1.47 (95% CI 1.28–1.68, Figs. 2 and 3) for patients with COPD. The absolute risk difference was 1.04% (95% CI 0.74–1.45%). The analysis showed considerable heterogeneity (I^2^ = 99.5%).

**Fig. 4:**
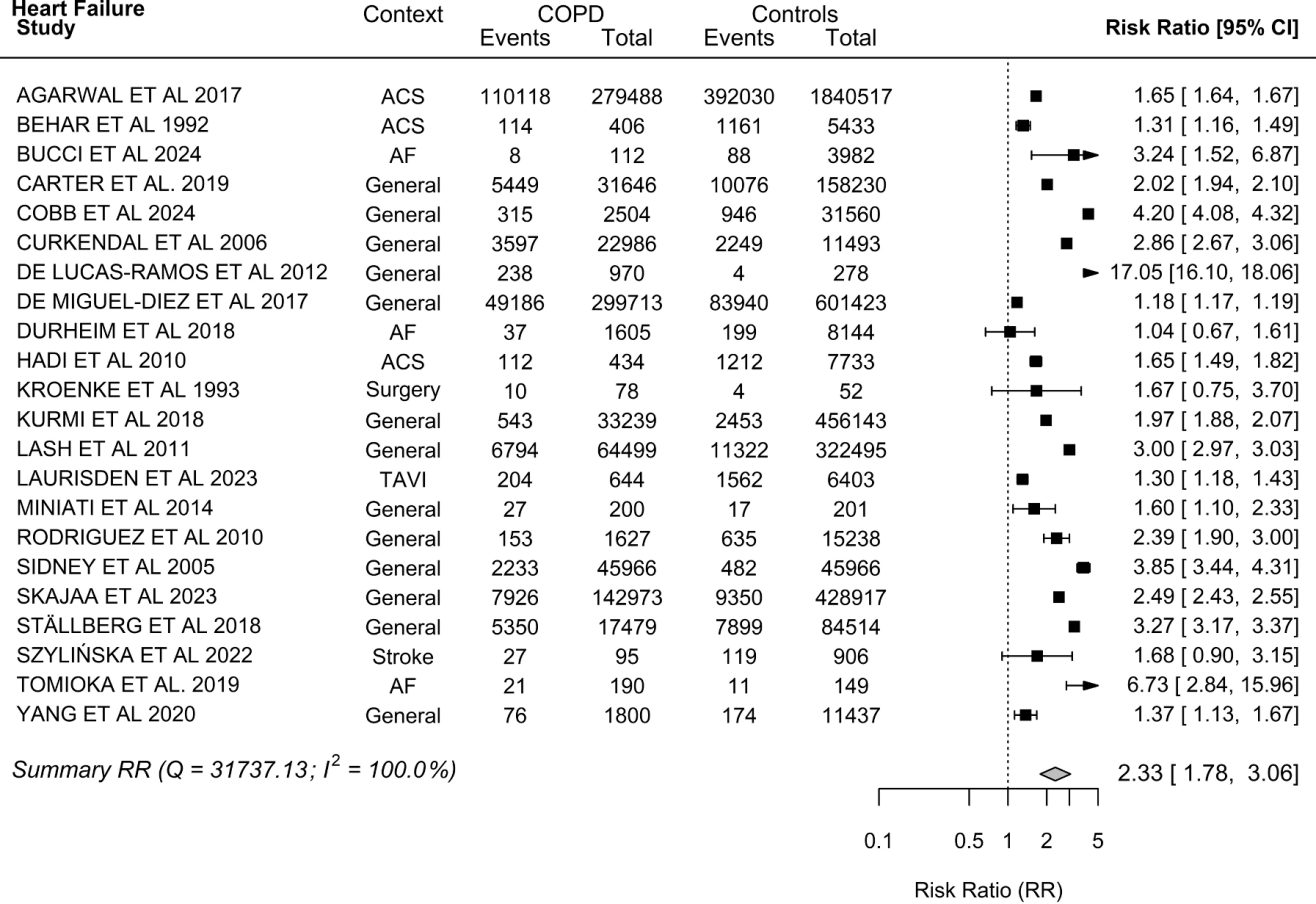
**Association between COPD and Heart Failure.** ACS—acute coronary syndrome; AF—atrial fibrillation; COPD—chronic obstructive pulmonary disease; CI—confidence interval; I^2^ denotes the heterogeneity statistic.; RR—risk ratio; TAVI—transcatheter aortic valve implantation.

22 studies^15^^,^^17^^,^^19^^,^^24^^,^^28^^,^^29^^,^^36^^,^^39^^,^^40^^,^^42^^,^^44^^,^^51^, ^52^, ^53^, ^54^, ^55^, ^56^, ^57^, ^58^, ^59^, ^60^, ^61^ including 4,989,868 patients (948,075 [19.0%] with COPD) reported heart failure as an outcome with a median follow-up of 3.0 years. The heart failure event rate in the control group was 5.60% (95% CI 3.48–8.89). The pooled relative risk was 2.33 (95% CI 1.78–3.06, Figs. 2 and 4) for patients with COPD. The absolute risk difference was 7.45% (95% CI 4.63–11.83%). The analysis showed considerable heterogeneity (I^2^ 100.0%).

Eight studies^43^^,^^62^, ^63^, ^64^, ^65^, ^66^, ^67^, ^68^ including 326,497 patients (69,880 [21.4%] with COPD) reported heart failure hospitalisation as an outcome with a median follow-up of 1.9 years. The heart failure hospitalisation event rate in the control group was 10.29% (95% CI 5.07–19.75%). The pooled relative risk was 1.78 (95% CI 1.28–2.47, Fig. 2) for patients with COPD. The absolute risk difference was 8.02% (95% CI 3.95–15.4%). The analysis showed considerable heterogeneity (I^2^ 99.5%). In subgroup analysis, the pooled relative risk was lower with increasing age (1.80 for age < 75 years compared to 1.75 for age ≥ 75 years).

17 studies^18^^,^^29^^,^^37^^,^^39^^,^^44^^,^^52^^,^^54^^,^^59^^,^^69^, ^70^, ^71^, ^72^, ^73^, ^74^, ^75^, ^76^, ^77^ including 833,506 patients (200,875 [24.1%] with COPD) reported atrial fibrillation as an outcome with a median follow-up of 5.2 years. The atrial fibrillation event rate in the control group was 7.66% (95% CI 4.59–12.50%). The pooled relative risk was 1.38 (95% CI 1.19–1.61, Fig. 2) for patients with COPD. The absolute risk difference was 2.95% (95% CI 1.77–4.81%) for patients with COPD. The analysis showed considerable heterogeneity (I^2^ 98.6%).

Four studies^57^^,^^78^, ^79^, ^80^ including 6788 patients (3075 [45.3%] with COPD) reported ventricular tachycardia as an outcome with a median follow-up of 0.1 years. The ventricular tachycardia event rate in the control group was 13.97% (95% CI 8.53–22.04%). The absolute risk difference was 14.70% (95% CI 8.97–23.20%). The pooled relative risk was 2.05 (95% CI 1.27–3.31, Fig. 2) for patients with COPD. The analysis showed considerable heterogeneity (I^2^ 84.6%).

There were insufficient studies to conduct a meta-analysis for atrial flutter or ventricular fibrillation.

42 studies^13^, ^14^, ^15^^,^^17^^,^^18^^,^^20^^,^^22^^,^^24^^,^^25^^,^^27^^,^^28^^,^^30^, ^31^, ^32^, ^33^, ^34^, ^35^^,^^37^, ^38^, ^39^, ^40^^,^^42^^,^^45^, ^46^, ^47^, ^48^, ^49^^,^^55^^,^^59^^,^^61^^,^^62^^,^^69^^,^^76^^,^^77^^,^^81^, ^82^, ^83^, ^84^, ^85^, ^86^, ^87^, ^88^ including 7,527,090 patients (1,211,862 [16.1%] with COPD) reported ischaemic stroke as an outcome with a median follow-up of 1.9 years. The stroke event rate in the control group was 2.26% (95% CI 1.72–2.97%). The pooled relative risk was 1.38 (95% CI 1.18–1.62, Fig. 2) for patients with COPD. There was no significant increase in absolute risk (0.86%, 95% CI 0.66–1.14%). The analysis showed considerable heterogeneity (I^2^ 98.9%).

13 studies^4^^,^^22^^,^^29^^,^^30^^,^^40^^,^^54^, ^55^, ^56^^,^^58^^,^^72^^,^^76^^,^^89^^,^^90^ including 2,195,145 patients (597,079 [27.2%] with COPD) reported peripheral arterial disease as an outcome with a median follow-up of 4.0 years. The peripheral arterial disease event rate in the control group was 5.56% (95% CI 2.69–11.12%). The pooled relative risk was 1.99 (1.49–2.65, Fig. 2) for patients with COPD. The absolute risk difference was 5.48% (95% CI 2.65–10.96%). The analysis showed considerable heterogeneity (I^2^ 99.9%).

**Fig. 5:**
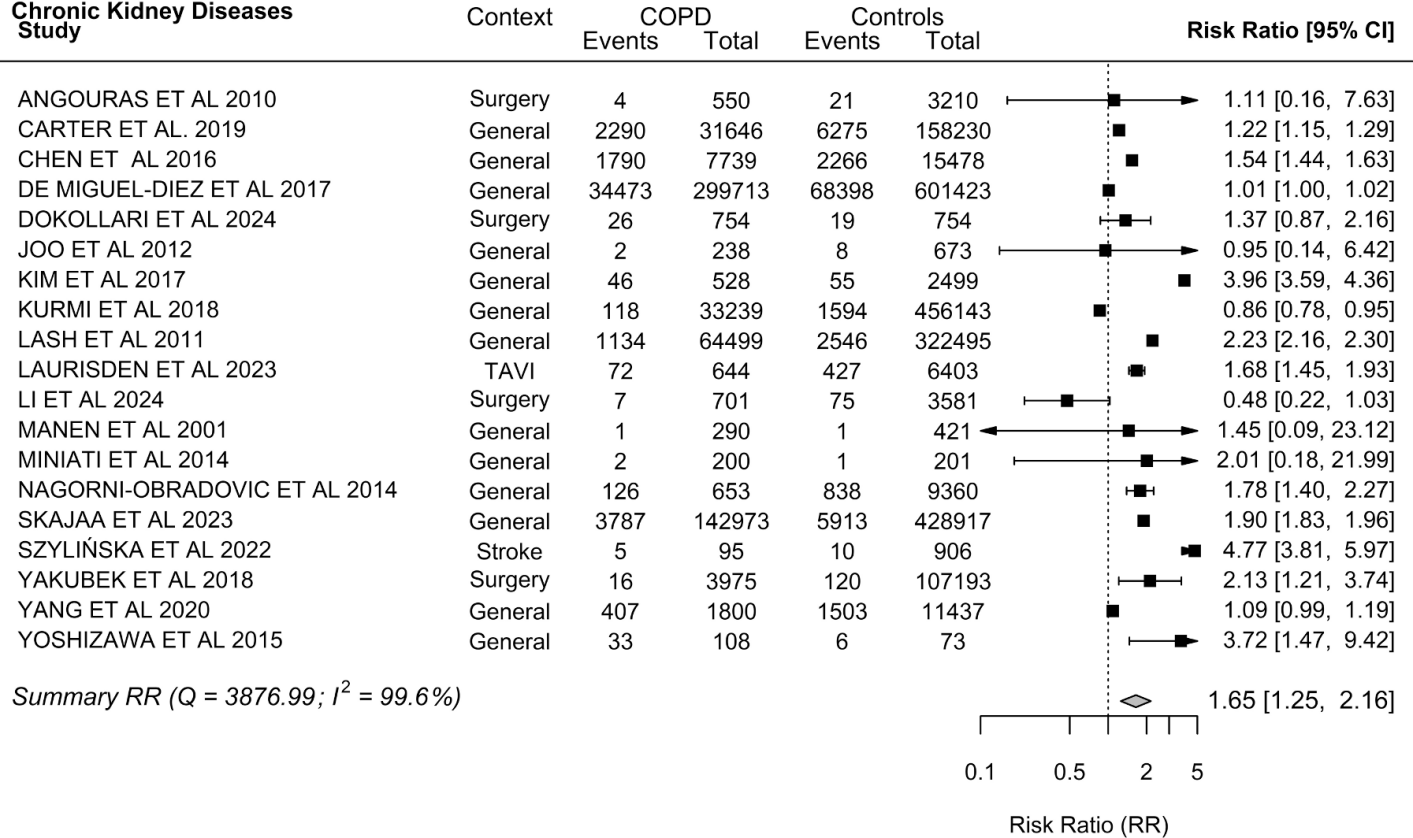
**Association between COPD and chronic kidney disease.** COPD—chronic obstructive pulmonary disease; CI—confidence interval; I^2^ denotes the heterogeneity statistic.; RR—risk ratio; TAVI—transcatheter aortic valve implantation.

19 studies^5^^,^^13^^,^^18^^,^^28^, ^29^, ^30^^,^^32^^,^^40^^,^^44^^,^^47^^,^^54^^,^^56^^,^^58^^,^^59^^,^^61^^,^^88^^,^^91^, ^92^, ^93^ including 2,719,742 (590,184 [21.7%] with COPD) reported chronic kidney disease as an outcome with a median follow up of 1.0 years. The chronic kidney disease event rate in the control group was 2.08% (95% CI 1.08–3.95%). The pooled relative risk was 1.65 (1.25–2.16, Figs. 2 and 5) for patients with COPD. The absolute risk difference was 1.34% (95% CI 0.7–2.56%). The analysis showed considerable heterogeneity (I^2^ 99.7%).

27 studies^14^^,^^19^^,^^25^^,^^28^^,^^35^^,^^36^^,^^39^^,^^46^^,^^48^^,^^49^^,^^53^^,^^59^^,^^60^^,^^63^^,^^65^^,^^67^, ^68^, ^69^^,^^84^^,^^94^, ^95^, ^96^, ^97^, ^98^, ^99^, ^100^, ^101^ including 6,368,920 patients (312,077 [4.9%] with COPD) reported cardiovascular death as an outcome with median follow-up of 3.0 years. The cardiovascular mortality event rate in the control group was 4.34% (95% CI 2.50–7.43%). The pooled relative risk was 1.55 (1.33–1.82, Fig. 2) for patients with COPD. The absolute risk difference was 2.4% (95% CI 1.38–4.10%). The analysis showed considerable heterogeneity (I^2^ 99.3).

93 studies^13^^,^^14^^,^^18^, ^19^, ^20^^,^^23^, ^24^, ^25^, ^26^, ^27^^,^^29^^,^^30^^,^^33^^,^^35^, ^36^, ^37^^,^^43^, ^44^, ^45^, ^46^, ^47^, ^48^^,^^50^, ^51^, ^52^, ^53^^,^^56^, ^57^, ^58^, ^59^, ^60^^,^^62^, ^63^, ^64^, ^65^^,^^67^, ^68^, ^69^^,^^73^, ^74^, ^75^^,^^77^^,^^81^, ^82^, ^83^, ^84^, ^85^^,^^94^, ^95^, ^96^, ^97^, ^98^, ^99^^,^^101^, ^102^, ^103^, ^104^, ^105^, ^106^, ^107^, ^108^, ^109^, ^110^, ^111^, ^112^, ^113^, ^114^, ^115^, ^116^, ^117^, ^118^, ^119^, ^120^, ^121^, ^122^, ^123^, ^124^, ^125^, ^126^, ^127^, ^128^, ^129^, ^130^^,^^131^, ^132^, ^133^, ^134^, ^135^, ^136^, ^137^, ^138^, ^139^, ^140^, ^141^, ^142^, ^143^, ^144^, ^145^, ^146^, ^147^, ^148^, ^149^, ^150^ including 19,936,003 patients (2,153,088 [10.8%] with COPD) reported all-cause mortality death as an outcome with a median follow-up of 1.94 years. The all-cause mortality event rate in the control group was 9.6% (95% CI 7.50–12.09%). The pooled relative risk was 1.55 (1.42–1.70, Fig. 2) for patients with COPD. The absolute risk difference was 5.30% (95% CI 4.16–6.70%). The analysis showed considerable heterogeneity (I^2^ 99.8).

Analyses for each of the events studied had considerable heterogeneity (I^2^ range 84.6%–100.0%). In the ten sensitivity analyses (design, follow up duration, removing high risk of bias, clinical context, continent, publication year, small sample sizes removed, COPD ascertainment, crude versus adjusted ratio, leave-one-out), the relative risks of outcomes remained consistent for heart failure, heart failure hospitalisation, peripheral arterial disease, chronic kidney disease, cardiovascular and all-cause mortality, and all demonstrated similar direction (i.e., increased) and magnitude for risk. For myocardial infarction, patients who underwent transcatheter aortic valve intervention had a lower relative risk (0.66, 95% CI 0.34–1.30). For atrial fibrillation, the sensitivity analysis for short study duration showed a mildly reduced relative risk (0.98, 0.70–1.37) of one study. For ventricular tachycardia, relative risks were lower in one study that was in the surgical context (0.83, 0.46–1.48). For ischaemic stroke, there was a reduced relative risk in patients undergoing transcatheter aortic valve intervention (0.89, 0.55–1.46) and coronary artery bypass grafting (0.58, 0.28–1.22). For both cardiovascular and all-cause mortality, risks remained elevated when study follow up was shorter or longer than one year. High levels of heterogeneity (I^2^ > 40%) continued to be observed in most sensitivity analyses (Supplementary Tables S6–S19).

COPD was variably defined, with several studies not reporting their criteria for COPD diagnosis (Supplementary Table S20). 25 studies included spirometry diagnosis whereas 50 were from administrative coding alone, and the remaining studies were based on a combination of chart review, self-reported or clinical judgement. Relative risks remained elevated regardless of COPD ascertainment method used, or the type of estimate used to synthesise results (crude versus adjusted) (Supplementary Tables S6–S19). Leave-one-out analyses showed similar risk estimates for all investigated outcomes (Supplementary Figs. S14–S25).

In subgroup analysis, the relative risk was higher when patients were younger than 75 years for myocardial infarction, heart failure, heart failure hospitalisation and ischaemic stroke (Supplementary Tables S6–S19). When samples were divided into risk categories (high versus low-risk context) derived risk estimates for all outcomes remained elevated, and all demonstrated significant heterogeneity. However, for all outcomes except chronic kidney disease, the low clinical risk group demonstrated high risk of incident cardiovascular and mortality events (Supplementary Tables S6–S19).

All outcomes had analyses conducted where high risk of bias studies were removed. The RR was 1.51 (95% CI 1.24–1.83, I^2^ 99.7%) for myocardial infarction, 2.14 (1.66–2.75, I^2^ 99.9%) for heart failure, 1.78 (1.28–2.47, I^2^ 99.5%) for heart failure hospitalisation, 1.43 (1.17–1.73, I^2^ 99.3%) for atrial fibrillation, 1.29 (1.04–1.61, I^2^ 99.2%) for stroke, 1.90 (1.30–2.78, I^2^ 99.9%) for peripheral arterial disease, 1.51 (1.09–2.09, I^2^ 99.8%) for chronic kidney disease, 1.51 (1.25–1.82, I^2^ 99.3%) for cardiovascular mortality and 1.49 (1.36–1.63, I^2^ 99.8%) for all-cause mortality. For both all-cause and cardiovascular mortality, the risk remained elevated whether follow up was shorted or longer than one year (Supplementary Tables S6–S19).

Univariate meta-regression revealed that COPD ascertainment by spirometry compared to clinical diagnosis was associated with an increased risk estimate of heart failure hospitalisation (2.36, 95% CI 1.38–4.02, p = 0.0016) and all-cause mortality over 1 year (1.97, 1.00–3.87, p = 0.049), however, COPD ascertainment by code compared to clinical diagnosis was associated with a lower risk of all-cause mortality within 1 year (0.61, 0.45–0.81, p < 0.0009). Additionally, high study-level mean age was associated with slight reductions in all-cause mortality risk within 1 year (0.97, 0.95–1.00, p = 0.030), over 1 year (0.96, 0.93–0.98, p = 0.0013) or any time frame (0.98, 0.97–0.99, p < 0.0004). Substantial heterogeneity remained. All other co-variates investigated did not demonstrate significant associations for all outcomes (all p > 0.05; Supplementary Table S21).

Multivariate meta-regression demonstrated that study-level prevalence hypertension (1.01, 95% CI 1.00–1.01, p = 0.012) and male sex (1.01, 1.00–1.01, p = 0.047) were associated with all-cause mortality. Study level prevalence of diabetes was associated with myocardial infarction (1.03, 1.00–1.07, p = 0.033). Substantial heterogeneity remained. All other co-variates investigated did not demonstrate significant associations for all outcomes (all p > 0.05; Supplementary Table S22).

The initial rating for certainty of evidence each domain was high, as non-randomised studies are the typical study choice for the investigation of associations with outcomes.^11^ However, considering the substantial heterogeneity present for all outcomes, even if effect estimates remained similar in direction and magnitude, led to all outcomes being downgraded to moderate. There was no evidence of dissemination bias, and outcome findings were robust to sensitivity analyses removing high risk of bias studies. Therefore, the final rating for certainty of evidence is moderate for all outcomes.

## Discussion

Synthesis of evidence from 140 case-control studies representing 30,144,481 individuals demonstrates the possible increased risk that COPD imparts on individuals in terms of new onset cardiovascular diseases, chronic kidney disease and death when compared to people without COPD. Such multisystem and syndemic effects include, but are not limited to, a 133% higher risk for heart failure, 99% higher risk for peripheral arterial disease, 65% higher risk for chronic kidney disease and 55% higher risk for cardiovascular death. The absolute risk for heart failure and heart failure hospitalisation was the highest among the events examined, although the precision of this estimate was limited by substantial study heterogeneity despite persistence of these associations across 10 sensitivity analyses. Furthermore, the certainty of evidence is moderate suggesting that the outcomes assessed are probably associated with the presence of COPD, however, given the imprecision of the estimate, the magnitude of this relationship cannot be confidently stated.^11^ In subgroup analyses, those in lower clinical risk scenarios were paradoxically at even higher risk of incident events across the majority of outcomes, and lower mean age also increased the risk for incident myocardial infarction, heart failure, heart failure hospitalisation and ischaemic stroke.

This study extends beyond the previous evidence base for the association of COPD with cardiovascular diseases. An earlier analysis of 27 studies reported a two-times increased pooled odds ratio for cardiovascular comorbidities yet had limited breadth for the association of COPD and of dysrhythmias, arterial diseases and heart failure, though did find a high overall risk for a cardiovascular disease.^6^ Another systematic review described the increased risk for atrial fibrillation and ventricular arrhythmias,^151^ but was again limited by including only a small number of studies with limited sample size, leading to possible underestimation of the absolute risk to people with COPD. There previously has been limited investigation into the association of COPD with chronic kidney disease, and whether COPD is associated with increased incidence, however a systematic review found that chronic kidney disease is a prevalent condition amongst those with COPD.^152^ Whilst our findings are consistent with these reports, we extend the evidence base by evaluating a much larger pooled population, different atherosclerotic cardiovascular diseases and arrhythmic events, as well as including related cardiovascular risk equivalents such as chronic kidney disease.^153^ In addition, the absolute risk estimated should be interpreted with caution given its sensitivity to baseline events rate and heterogeneity in studies. We included these to provide important clinical context, as knowledge of both the estimated relative and absolute risks may impact clinical decision making. To our knowledge, this is the largest study to date evaluating the association of COPD with incident cardio-kidney diseases. Our findings are consistent through sensitivity analyses, demonstrating that COPD could be close to the cardio-kidney-metabolic axis considering the range of associations suggested by this study.

COPD is associated with cardiovascular diseases and chronic kidney diseases based on shared risk factors, including smoking, chronic inflammation, socioeconomic deprivation across the life-course, obesity, reduced activity, amongst others.^152^^,^^154^ COPD can also have direct cardiovascular effects – static and dynamic hyperinflation can affect cardiac performance, hypoxic vasoconstriction results in increased pulmonary vascular resistance and can increase right ventricular afterload and simultaneously reduce left ventricular preload.^154^^,^^155^ Chronic hypoxia is well established as a contributing factor for atherosclerotic disease, and can accelerate the development of peripheral vascular disease, ischaemic heart disease, and chronic kidney disease (either as interstitial damage, or progression of atherosclerotic disease to the kidney vessels).^156^ Chronic kidney diseases and cardiovascular diseases are more prone to develop in those with hypertension, diabetes and current smokers.^157^ It is logical, therefore, that those at increased risk of cardiovascular diseases should be at increased risk of incident kidney diseases. This close relationship between cardiovascular and kidney disease (mediated by risk factors and comorbidities) represents a target that could impact multi-organ health.^158^ There are increased treatment options that have a range of beneficial metabolic and kidney effects which confers protection against adverse cardiovascular events and mortality (such as glucagon-like 1 peptide receptor agonists and sodium-glucose co-transporter 2 inhibitors). Identifying those most likely to benefit from such interventions is prudent in effective resource allocation.^158^ Moreover, pulmonary diseases may align through a cardio-kidney-metabolic-pulmonary syndrome.

Beyond broad associations with cardiovascular-kidney disease in COPD, our sensitivity analyses and estimation of absolute risk identified potentially clinically actionable novel findings to inform public health resourcing. First, general population-specific analyses generally provided the highest relative risk estimates for all outcomes when compared to specific clinical contexts (e.g. post-acute coronary syndrome or transcatheter aortic valve intervention) – and reinforced by the low versus high risk subgroup analysis – suggesting that addressing cardio-kidney risk in patients with COPD in the community may provide the greatest overall benefit. Second, those aged below 75 years with COPD were at higher relative risk for adverse cardiovascular-kidney events than older individuals. Thus, while traditionally regarded as a disease among older individuals, COPD in younger individuals could represent a modifiable cardiovascular risk factor and cardiovascular disease could represent a modifiable COPD risk factor. Third, heart failure had the largest absolute risk increase amongst all investigated outcomes, more so than atherosclerotic cardiovascular diseases. While we could not ascertain the type of heart failure (preserved versus reduction ejection fraction), the prevention of heart failure in COPD,^159^ and reduction of adverse outcomes for those with co-existent COPD and heart failure (regardless of type) are important therapeutic targets. Meta-analyses and post-hoc analyses of randomised clinical trials have suggested that triple therapy may delay or reduce cardiovascular events in patients with COPD.^160^^,^^161^ Furthermore, our findings of those in lower clinical risk settings were at higher risk of incident events highlights the possibility that those undergoing high clinical risk care are more likely to be medically optimised. This reinforces the idea that community dwelling individuals with COPD likely have undertreated cardio-kidney-metabolic-pulmonary risk that can be mitigated. Our findings suggest that the impact of current COPD treatments on a broader range of cardiovascular and kidney outcomes requires prospective randomised assessment. Furthermore, whether treatments that impact upon the cardio-kidney axis improves outcomes in patients with COPD is unknown and warrants further study.

There are limitations to this study. First, it was based on reported results of independent published studies and may not have included some unpublished studies, although we did not find evidence of publication bias for any outcome. Second, despite our extensive search strategy, we may not have identified all studies. However, the number of studies in our analysis makes our results robust to single studies inadvertently omitted. Third, there was substantial heterogeneity for the majority of outcomes, which is not unexpected and likely caused by differences in study methods, study and patient characteristics, and changes in medical therapy between studies and study periods. Fourth, definitions for COPD and outcomes varied across studies, and we cannot exclude misclassification. Significant differences in both outcome definitions and the ascertainment of COPD status can further add imprecision to the effect estimates – likely representing a source of heterogeneity. Additionally, meta-regression could not identify a specific cause for heterogeneity. Variations in definitions is a frequent challenge and limits the ability to harmonise data from multiple sources to synthesise effect estimates. This difficulty has led to data standardisation efforts to ensure harmonised data collection.^162^^,^^163^ Fifth, despite our extensive search strategy, we did not identify sufficient studies to enable meta-analysis for atrial flutter and ventricular fibrillation. Sixth, though some studies reported COPD severity, classification was variable, and many studies did not report the severity of airflow obstruction (GOLD stage), which made it difficult to quantify the risk for each outcome in relation to COPD severity. This could limit clinical interpretation, as this study cannot identify the COPD stage at most risk of incident cardio-kidney outcomes. However, this study does demonstrate an association that should prompt clinicians to consider investigation and potential intervention to prevent adverse outcomes. Seventh, certain sensitivity analyses were complicated by incomplete reporting of specific data (e.g. sex-/ethnicity-specific outcomes and aetiology of COPD). However, in the other sensitivity analyses performed the relative risks computed were generally consistent. Eighth, we cannot disregard the potential of residual confounding that could be contributing to the substantial heterogeneity demonstrated and could reduce the perceived strength of associations demonstrated. This is in part due to inconsistent COPD and outcome definitions, and ascertainment of outcomes. This could further affect the robustness of the estimates generated from this analysis and should be interpreted with caution. However, the risks persistently demonstrated similar direction and magnitude across sensitivity and subgroup analyses. Furthermore, risk estimates are made on summary level estimates rather than individual patient data meaning only associations can be drawn from any risk factor, and this further limits the meta-regression analyses as they are based on summary level data. In addition, although we combined adjusted and unadjusted risk estimates which may introduce bias, the findings were consistent across sensitivity analyses and meta-regression analyses suggesting that the overall conclusions were not materially affected by the inclusion of different estimate types. Finally, though using individual patient data is preferred, this would not be practicable with such large combinations of data across an extensive number of studies.

In summary this comprehensive evidence synthesis demonstrates an association of COPD with a range of incident cardiovascular-kidney events and death. Future studies and strategies are required to prevent such disease onset and therefore improve prognosis.

## Contributors

TJ, CPG and RN were involved in the conception and design of the study. CG and JW provided statistical analysis. TJ, ID, AM, KR, MH, UN, LE, LN screened titles, abstracts and completed data extraction. All authors were involved in the critical revision of the manuscript for important intellectual content. TJ was responsible for drafting the manuscript. TJ, CG and JW verified the underlying data. TJ, CG and JW had access to the underlying data. All authors (TJ, CG, RN, RA-L, LA, DDB, AC, ID, LE, MH, NH, JRH, CRJ, AK, JWHK, KK, CSPL, TL, FM, AM, MFMA, UN, LN, KW, RP, KR, CKR, NS, DS, YS, GT, TW, DBP, MB, JW, CPG) read and approved the final version of this manuscript and agree to be accountable for all aspects of the work.

## Data sharing statement

Data generated or analysed during this study are available from the corresponding author following reasonable written request.

## Declaration of interests

**Tobin Joseph** received funding directly for this study (CRCRDF-PCCS030425JOSEPH). The funding source did not influence the study design, data collection, analysis, interpretation of data, manuscript preparation, or the decision to submit the paper for publication. He also reports receiving honoraria from Janssen-Cilag Ltd for invited speaking activities and was also an invited co-author for a publication in *Medicine*.

**Chenyi Gao** is supported by Barts Charity (MGU0504, G-002982) and National Institute for Health and Care Research (NIHR) School for Primary Care Research Career Development Fund C179 (Chenyi Gao). The views expressed are those of the author(s) and not necessarily those of the NIHR or the Department of Health and Social Care.

**Ramesh Nadarajah** is supported by the NIHR and HDR UK. He has received support for attendance at meetings from Medtronic. He serves on a trial steering committee for Boston Scientific. He has received personal fees from Vitacam. He has received grants for research from British Heart Foundation, National Institute for Health Research, and Leeds Hospital Charity.

**Riyad Al-Lehebi** has given lectures at meetings supported by AstraZeneca, Boehringer Ingelheim, Novartis, GlaxoSmithKline, and Sanofi, and participated in advisory board fees from GlaxoSmithKline, AstraZeneca, Novartis, and Abbott.

**Luís Alves** has served as an advisor or consultant for AstraZeneca, GlaxoSmithKline and Merck Sharp & Dohme; served as a speaker or a member of a speakers bureau for AstraZeneca, GlaxoSmithKline, BIAL, Viatris, Abbott, and Novartis Pharmaceuticals Corporation.

**David D Berg** has received institutional research grant support through Brigham and Women’s Hospital from AstraZeneca, Merck, and Pfizer; consulting fees from AstraZeneca, Pfizer, and Youngene Therapeutics; honoraria from the Metabolic Endocrine Education Foundation, Pri-Med, Radcliffe Cardiology, and USV Private Limited; and participates on clinical endpoint committees for studies sponsored by Beckman Coulter, CeleCor Therapeutics, Kowa Pharmaceuticals, Novo Nordisk, and Tosoh Biosciences.

**Amy Couper** is contracted by the Observational and Pragmatic Research Institute (OPRI) Pte Ltd, Singapore, and Optimum Patient Care Global.

**Indranil Datta** reports no conflict of interest.

**Loay Eleyan** reports no conflict of interest.

**Mohammad Haris** reports no conflict of interest.

**Nathanial Hawkins** reports participation on advisory boards for Bayer and GSK; payment or honoraria for presentations and educational events from AstraZeneca; grants from AstraZeneca; and consulting fees from AstraZeneca.

**John R Hurst** has grant support from AstraZeneca, and has received personal payment and payment to his institution for educational and advisory work from AstraZeneca, Boehringer Ingelheim, Chiesi, GlaxoSmithKline and Sanofi.

**Christine R Jenkins** has received grants or institutional support, advisory board fees, educational support, consultation fees, travel support, and honoraria from AstraZeneca, GlaxoSmithKline, Chiesi, Sanofi, and Boehringer Ingelheim. She has also received consulting fees from AstraZeneca, GlaxoSmithKline, Sanofi, and Chiesi; honoraria for lectures, presentations, and educational activities from AstraZeneca, GSK, Sanofi, Chiesi, Orion Pharma, Mundipharma, and Boehringer Ingelheim; expert testimony fees from GlaxoSmithKline; and travel support for speaking engagements from AstraZeneca, GlaxoSmithKline, Sanofi, Chiesi, Orion Pharma, Mundipharma, and Boehringer Ingelheim. She serves on the unpaid LAMA by Night Advisory Board and is an unpaid Director of Lung Foundation Australia and the Asbestos and Dust Diseases Research Institute. She has no conflicts in relation to this work.

**Alan Kaplan** is a member of the advisory board of, or speakers bureau for, ALK, AstraZeneca, Belus, Boehringer Ingelheim, Covis, Eisai, GlaxoSmithKline, Idorsia, Merck Frosst, Moderna, Novo Nordisk, Novartis, Pfizer, Purdue, Sanofi, Teva, Trudel and Valeo.

**Janwillem W. H. Kocks** reports grants, personal fees and non-financial support from AstraZeneca, grants, personal fees and non-financial support from Boehringer Ingelheim, grants and personal fees from Chiesi, grants, personal fees and non-financial support from GSK, non-financial support from Mundi Pharma, grants and personal fees from Teva, personal fees from MSD, personal fees from COVIS Pharma, personal fees from ALK-Abello, grants from Valneva outside the submitted work; and Janwillem Kocks holds <5% shares of Lothar Medtec GmbH and is owner of the General Practitioners Research Institute.

**Konstantinos Kostikas** has received honoraria for presentations and/or consultancy fees from AstraZeneca, Berlin-Chemie, Boehringer Ingelheim, Chiesi, ELPEN, GSK, Guidotti, Menarini, Pfizer, Sanofi, and Specialty Therapeutics. His department has received funding and/or grants from AstraZeneca, Boehringer Ingelheim, Chiesi, ELPEN, GSK, Menarini. He worked with AstraZeneca as Global Medical Head Respiratory Biologics—02.09.2024 to 29.11.2024. He is a member of the GOLD Assembly.

**Carolyn Su Ping Lam** has received research grants from the National Medical Research Council of Singapore, Novo Nordisk, and Roche Diagnostic; has received consulting fees from Alnylam Pharma, AnaCardio AB, Applied Therapeutics, AstraZeneca, Bayer, Biopeutics, Boehringer Ingelheim, Boston Scientific, Bristol Myers Squibb, Corteria, CPC Clinical Research, Cytokinetics, Eli Lilly, Impulse Dynamics, Intellia Therapeutics, Janssen Research & Development LLC, Medscape/WebMD Global LLC, Merck, Novartis, Novo Nordisk, Quidel Corporation, Radcliffe Group Ltd., Roche and Us2.ai; has patent PCT/SG2016/050217 pending and patent US Patent No. 10,631,828 B1; US 10,702,247 B2; US 11,301,996 B2; US 11,446,009 B2; US 11,931,207 B2; US 12,001,939; US 12,400,762 B2; and is a co-founder and non-executive director of Us2.ai.

**Therese S. Lapperre** has received research support from Genentech, Chiesi; and support for attending meetings and/or travel from AstraZeneca and Sanofi. She has participated in advisory boards from AstraZeneca, Chiesi, Sanofi, GSK; and has received payment or honoraria for lectures, presentations, manuscript writing or organisation of educational events from AstraZeneca, Boehringer Ingelheim, Chiesi, GSK, Novartis, Sanofi, Genentech, Roche.

**Fernando Martinez** has received personal fees and non-financial support from the American College of Chest Physicians, AstraZeneca, Boehringer Ingelheim, ConCert, Genentech, GSK, Inova Fairfax Health System, Miller Communications, National Society for Continuing Education, Novartis, Pearl Pharmaceuticals, PeerView Communications, Prime Communications, Puerto Rico Respiratory Society, Chiesi, Sunovion, Theravance, Potomac, University of Alabama Birmingham, Physicians Education Resource, Canadian Respiratory Network, Teva and Dartmouth, Sanofi/Regeneron and payments were made to the COPD Foundation for partnership in SPIROMICS and/or CAPTURE; non-financial support from ProterrixBio, Gilead, Nitto and Zambon; and personal fees from Columbia University, Integritas, MD magazine, Methodist Hospital Brooklyn, New York University, UpToDate, WebMD/MedScape, Western Connecticut Health Network, Patara/Respivant, PlatformIQ, American Thoracic Society, Rockpointe, Rare Disease Healthcare Communications and France Foundation; grant support from NIH; and is a member of steering committees for Afferent/Merck, Biogen, Veracyte, Prometic, Bayer, Bridge Biotherapeutics and ProMedior.

**Alexandra Mircescu** reports no conflict of interest.

**Maria Felicia Montero- Arias** has given lectures at meetings supported by Astra Zeneca, GlaxoSmithKline, Novartis, Boehringer, Ferrer, Roche and FAES pharma; has participated in advisory boards for Astra Zeneca, GlaxoSmithKline, Novartis and Boehringer; has received support for attendance at meetings or congress from AstraZeneca, GlaxoSmithKline, Novartis, Boehringer, and Ferrer and has received research fees or grants from Astra Zeneca, Moderna, Novartis and GlaxoSmithKline. President of the Costa Rican Respiratory Society.

**Umbreen Nadeem and Long Nguyen** report no conflict of interest.

**Kate Wignall** and **Rachel Pullen** report no conflict of interest.

**Keerthenan Raveendra** reports no conflict of interest.

**Chin Kook Rhee** received consulting/lecture fees from Merck Sharp & Dohme, AstraZeneca, GlaxoSmithKline, Novartis, Takeda, Mundipharma, Boehringer Ingelheim, Teva, Sanofi, Organon, Roche, and Bayer.

**Naveed Sattar** has consulted for and/or received speaker honoraria from Abbott Laboratories, AbbVie, Amgen, AstraZeneca, Boehringer Ingelheim, Carmot Therapeutics, Eli Lilly, GlaxoSmithKline, Hanmi Pharmaceuticals, Menarini-Ricerche, Metsera, Novartis, Novo Nordisk, Pfizer, and Roche; and received grant support paid to his University from AstraZeneca, Boehringer Ingelheim, Novartis, and Roche outside the submitted work.

**Daiana Stolz** reports the position of GOLD representative for Switzerland, lecture honoraria from AstraZeneca, Berline-Chemie/Menarini, Boehringer Ingelheim, Chiesi, CSL Behring, GSK, Merck, MSD, Sanofi, Vifor, and Roche; data safety monitoring/advisory board participation with AstraZeneca, Berline-Chemie/Menarini, Boehringer Ingelheim, Chiesi, CSL Behring, Curetis AG, GSK, Merck, MSD, Roche, Novartis, Sanofi and Vifor, and grants to the institution from OM Pharma outside submitted work.

**Yongchang Sun and Gary Tse** report no conflicts of interests.

**Tonya Winders** reports no conflicts of interest. **David B. Price** has advisory board membership with AstraZeneca, Boehringer Ingelheim, Chiesi, GlaxoSmithKline, Novartis, Viatris, Teva Pharmaceuticals; consultancy agreements with AstraZeneca, Boehringer Ingelheim, Chiesi, GlaxoSmithKline, Novartis, Viatris, Teva Pharmaceuticals; grants and unrestricted funding for investigator-initiated studies (conducted through Observational and Pragmatic Research Institute Pte Ltd) from AstraZeneca, Chiesi, Viatris, Novartis, Regeneron Pharmaceuticals, Sanofi Genzyme, and UK National Health Service; payment for lectures/speaking engagements from AstraZeneca, Boehringer Ingelheim, Chiesi, Cipla, Inside Practice, GlaxoSmithKline, Medscape, Viatris, Novartis, Regeneron Pharmaceuticals and Sanofi Genzyme, Teva Pharmaceuticals; payment for travel/accommodation/meeting expenses from AstraZeneca, Boehringer Ingelheim, Novartis, Medscape, Teva Pharmaceuticals.; owns 74% of the social enterprise Optimum Patient Care Ltd (Australia and UK) and 92.61% of Observational and Pragmatic Research Institute Pte Ltd (Singapore); is peer reviewer for grant committees of the UK Efficacy and Mechanism Evaluation Programme, and Health Technology Assessment; and he was an expert witness for GlaxoSmithKline.

**Mohit Bhutani** received grants paid to his institution by CIHR, AstraZeneca, Boehringer Ingelheim, GlaxoSmithKline, Sanofi and Mereo. MB received speaker and consulting fees from AstraZeneca, Boehringer Ingelheim, GlaxoSmithKline, Sanofi, Valeo and Covis.

**Jianhua Wu** is supported by Barts Charity (MGU0504).

**Chris P Gale** reports honoraria from AstraZeneca, Amgen, Bayer, Boehrinher-Ingelheim, Chiesi, Daiichi Sankyo, GlaxoSmithKline, Lung Health Foundation, Menarini, Novartis, iRhythm. He has received research grants to his Institution from BMS, Abbott inc., Daiichi Sankyo, British Heart Foundation, National Institute of Health Research, Horizon 2020, and European Society of Cardiology, outside the submitted work.
