# Supplementary material for: Chronic obstructive pulmonary disease and risk of incident cardiovascular disease, chronic kidney disease, and death: a systematic review and meta-analysis
Source: eClinicalMedicine. 2026 Aug 7;98:104131. doi: 10.1016/j.eclinm.2026.104131 (PMC13475657; doi:10.1016/j.eclinm.2026.104131)
Supplement: Supplementary file 1 [file mmc1.pdf]

Cardiovascular, chronic kidney disease and mortality risk in Chronic Obstructive Pulmonary Disease: A Systematic Review and Meta Analysis

Contents

SUPPLEMENTARY RESULTS 3

Supplementary Table 1: Search Strategy Results for MEDLINE 3

Supplementary Table 2: Search Strategy Results for Embase and Embase Classic 5

Supplementary Table 3: Definition of Outcomes Excluding for All Cause Mortality. 7

Supplementary Table 4: Characteristics of Excluded Studies 13

Supplementary Table 5: Characteristics of Included Studies 16

Supplementary Table 6: Sensitivity and subgroup analysis for Myocardial Infarction 20

Supplementary Table 7: Sensitivity and subgroup analysis for Heart Failure 21

Supplementary Table 8: Sensitivity and subgroup analysis for Heart Failure Hospitalisation 22

Supplementary Table 9: Sensitivity and subgroup analysis for Atrial Fibrillation 23

Supplementary Table 10: Sensitivity and subgroup analysis for Ventricular Tachycardia 24

Supplementary Table 11: Sensitivity and subgroup analysis for Stroke 25

Supplementary Table 12: Sensitivity and subgroup analysis for Peripheral Arterial Disease 26

Supplementary Table 13: Sensitivity and subgroup analysis for Chronic Kidney Disease 27

Supplementary Table 14: Sensitivity and subgroup analysis for Cardiovascular Mortality 28

Supplementary Table 15: Sensitivity and subgroup analysis for Cardiovascular Mortality < 1 year 29

Supplementary Table 16: Sensitivity and subgroup analysis for Cardiovascular Mortality >1 year 30

Supplementary Table 17: Sensitivity and subgroup analysis for All-cause Mortality 31

Supplementary Table 19: Sensitivity and subgroup analysis for All-cause Mortality over 1 year 33

Supplementary Table 20: COPD ascertainment for each study 34

Supplementary Table 21: Univariate meta-regression for significant associations between covariate and outcome 46

Supplementary Table 22: Multivariate meta-regression for significant associations between covariate and outcome 47

Supplementary Figure 1: Risk of bias assessment for each domain in each study 48

Supplementary Figure 3: Funnel plot for myocardial infarction 52

Supplementary Figure 4: Funnel plot for Heart Failure 53

Supplementary Figure 5: Funnel plot for Heart Failure Hospitalisation 54

Supplementary Figure 6: Funnel plot for Atrial Fibrillation 55

Supplementary Figure 7: Funnel plot for Stroke 56

Supplementary Figure 8: Funnel plot for Peripheral Arterial Disease 57
Supplementary Figure 10: Funnel plot for Cardiovascular Mortality 59
Supplementary Figure 11: Funnel plot for All-cause Mortality 60
Supplementary Figure 12: Funnel plot for All-cause Mortality <1 year 61
Supplementary Figure 13: Funnel plot for All-cause Mortality over 1 year 62
Supplementary Figure 14: Leave one out analyses for myocardial infarction 63
Supplementary Figure 15: Leave one out analyses for heart failure 64
Supplementary Figure 16: Leave one out analyses for heart failure hospitalisation.................. 65
Supplementary Figure 17: Leave one out analyses for atrial fibrillation 66
Supplementary Figure 18: Leave one out analyses for stroke 67
Supplementary Figure 19: Leave one out analyses for peripheral arterial diseases 68
Supplementary Figure 20: Leave one out analyses for cardiovascular mortality....................... 69
Supplementary Figure 21: Leave one out analyses for cardiovascular mortality within 1 year... 70
Supplementary Figure 22: Leave one out analyses for cardiovascular mortality over 1 year 71
Supplementary Figure 23: Leave one out analyses for all-cause mortality............................... 72
Supplementary Figure 24: Leave one out analyses for all-cause mortality within one year 74
Supplementary Figure 25: Leave one out analyses for all-cause mortality over one year.......... 75
REFERENCES 76

SUPPLEMENTARY RESULTS

Supplementary Table 1: Search Strategy Results for MEDLINE

#	Query	Results from November 24th, 2025
1	exp Pulmonary Disease, Chronic Obstructive/	73925
2	bronchitis, chronic/ or Bronchitis/ or Bronchiolitis/	26587
3	(chronic obstructive pulmonary disease or COPD).tw.	90977
4	(bronchiolitis or bronchitides or bronchitis).tw.	37916
5	chronic obstructive pulmonary disease.tw.	69964
6	Lung Diseases, Obstructive/	18322
7	emphysema/ or mediastinal emphysema/ or subcutaneous emphysema/ or alpha 1-antitrypsin deficiency/	16819
8	emphysema.tw.	28048
9	obstructive lung disease?.tw.	8419
10	obstructive airway disease?.tw.	1585
11	(chronic obstructive airways disease or COAD).tw.	1992
12	or/1-11	189896
13	(mortality or death* or cardiovascular or vascular or cardiac or coronary or heart or cerebrovascular or myocardial or stroke or athero* or arter* or kidney or renal).tw.	5855126
14	(CKD or CAD or CVD or PAD or PVD or ESRF or ESKD or CKD or PACS or TACS or LACS or POCS or NSTEMI or STEMI or SCD or HF).tw.	330583
15	(AECOPD or IECOPD or NIECOPD or ecopd or exacerbation).tw.	52985
16	exp CARDIOVASCULAR DISEASES/	2907327
17	atrial fibrillation/ or atrial flutter/ or ventricular tachycardia/ or ventricular fibrillation/	114272
18	(AF or flutter or fibrillation or A flutter or VT or vf or ventricular tachycardia or ventricular fibrillation).tw.	197897
19	VASCULAR DISEASES/	40328
20	exp CEREBROVASCULAR DISORDERS/	466503
21	exp HEART FAILURE/	162862
22	STROKE/	148512
23	exp MYOCARDIAL INFARCTION/	203716
24	exp DEATH, SUDDEN, CARDIAC/	19272
25	exp KIDNEY FAILURE, CHRONIC/	105568
26	exp RENAL INSUFFICIENCY, CHRONIC/	147275
27	exp PERIPHERAL ARTERIAL DISEASE/	21401
28	exp PERIPHERAL VASCULAR DISEASES/	67985
29	exp ATHEROSCLEROSIS/	64468
30	exp hospitalization/	323932
31	(hospitali?ation or admission or discharge or readmission or re-admission).tw.	702598
32	or/13-31	7537747
33	PROSPECTIVE STUDIES/	734370
34	OBSERVATIONAL STUDY/	187515
35	COHORT STUDIES/	365619
36	LONGITUDINAL STUDIES/	191720

37	MULTICENTER STUDY/	385452
38	COMPARATIVE STUDY/	1967514
39	FOLLOW-UP STUDIES/	722374
40	PREVALENCE/	382009
41	INCIDENCE/	328589
42	or/33-41	4250099
43	RISK/	129174
44	RISK FACTORS/	1060287
45	risk.tw.	3271023
46	or/43-45	3641388
47	12 and 32 and 42 and 46	9165
48	limit 47 to humans	9150

Supplementary Table 2: Search Strategy Results for Embase and Embase Classic

#	Query	Results from November 24th, 2025
1	exp chronic obstructive lung disease/	220522
2	bronchitis, chronic/ or Bronchitis/ or Bronchiolitis/	77487
3	(chronic obstructive pulmonary disease or COPD or bronchiolitis or bronchitides or bronchitis).tw.	224837
4	emphysema/ or mediastinal emphysema/ or subcutaneous emphysema/ or alpha 1-antitrypsin deficiency/	54558
5	emphysema.tw.	49265
6	obstructive lung disease?.tw.	14478
7	obstructive airway disease?.tw.	2865
8	(chronic obstructive airways disease or COAD).tw.	2448
9	1 or 2 or 3 or 4 or 5 or 6 or 7 or 8	390846
10	(mortality or death* or cardiovascular or vascular or cardiac or coronary or heart or cerebrovascular or myocardial or stroke or athero* or arter* or kidney or renal).tw.	8762273
11	(CKD or CAD or CVD or PAD or PVD or ESRF or ESKD or CKD or PACS or TACS or LACS or POCS or NSTEMI or STEMI or SCD or HF).tw.	588424
12	(AECOPD or IECOPD or NIECOPD or ecopd or exacerbation).tw.	95853
13	exp cardiovascular disease/	6486687
14	exp atrial fibrillation/	269748
15	exp heart atrium flutter/	22159
16	heart ventricle tachycardia/ or heart ventricle arrhythmia/	94417
17	heart ventricle fibrillation/	46576
18	(AF or flutter or fibrillation or A flutter or VT or vf or ventricular tachycardia or ventricular fibrillation).tw.	354243
19	vascular disease/ or peripheral vascular disease/	121649
20	heart failure/	401009
21	stroke/ or myocardial infarction/	808086
22	heart death/	36680
23	chronic kidney failure/	262728
24	(hospitali?ation or admission or discharge or readmission or re-admission).tw.	1224295
25	hospitalization/	698762
26	10 or 11 or 12 or 13 or 14 or 15 or 16 or 17 or 18 or 19 or 20 or 21 or 22 or 23 or 24 or 25	12080939
27	prospective study/	1024954
28	observational study/	504908
29	COHORT STUDIES/	1506443
30	LONGITUDINAL STUDIES/	266151
31	MULTICENTER STUDY/	528478
32	COMPARATIVE STUDY/	1185421
33	FOLLOW-UP STUDIES/	2615622
34	PREVALENCE/	1192835
35	INCIDENCE/	733777
36	27 or 28 or 29 or 30 or 31 or 32 or 33 or 34 or 35	7217383

37	risk/ or risk factors/	2065879
38	9 and 26 and 36 and 37	17226
39	animal/ not human/	1679264
40	nonhuman/	8419849
41	exp animal experiment/	3478298
42	exp experimental animal/	958685
43	animal model/	2001012
44	exp rodent/	4899226
45	(rat or rats or mouse or mice).ti.	1934018
46	39 or 40 or 41 or 42 or 43 or 44 or 45	11462376
47	letter.pt. or letter/	1411833
48	note.pt.	1029433
49	conference abstract.pt.	5752873
50	editorial.pt.	855597
51	case report/ or case study/	3412490
52	(letter or comment*).ti.	279217
53	47 or 48 or 49 or 50 or 51 or 52	11727525
54	46 or 53	21770853
55	38 not 54	11208

Supplementary Table 3: Definition of Outcomes Excluding for All Cause Mortality.

Author	Outcome definition
Agarwal et al 2017[1]	heart failure: Secondary outcomes included new-onset HF (defined as patients without a past history of HF but with HF at the time of discharge). ICD-9-CM codes and Clinical Classification Software codes used to identify baseline characteristics are provided in e-Table 1.
Andell et al 2017[2]	Stroke, AF, CV mortality - NR
Angouras et al 2010[3]	Stroke, CKD, MI: NR
Behar et al 1992[4]	AF: paroxysmal, not otherwise defined; HF: not defined
Bucci et al 2024[5]	HF: not defined; cardiovascular mortality: death due to fatal cardiac (acute coronary syndromes, heart failure, arrhythmia, cardiac perforation, tamponade, or other unspecified cardiac causes) or vascular (ischemic stroke, hemorrhagic stroke, systemic hemorrhages, peripheral embolism, and pulmonary embolism) events
Buklioska-Ilievska et al 2019[6]	PAD: Doppler ultrasonography of lower limb arteries was performed with the same General Electric Vivid 7 ultrasound system receiving information about the localisation, extensiveness and severity of vascular lesions
Buzea et al 2016[7]	VT: specifically 4 or more consecutive ventricular impulses
Campo et al 2013[8]	HF hospitalisation: NR; Stroke: NR
Canepa et al 2017[9]	cardiovascular mortality: cause of death was obtained from mortality registry
Carter et al. 2019[10]	HF: ICD-10 and OPCS-4 coding; AF: ICD-10 and OPCS-4 coding; CKD: ICD-10 and OPCS-4 coding; PAD: ICD-10 and OPCS-4 coding
Chen et al 2016[11]	CKD: NR
Chiang et al 2022[12]	cardiovascular mortality: Mortality from acute myocardial infarction, STEMI and NSTEMI
Chopard et al. 2014[13]	cardiovascular mortality: NR; Stroke: NR; MI:NR
Cobb et al 2024[14]	HF, MI, Stroke: Cardiovascular disease status as determined by a response of 'Yes' to any interview question about a doctor or medical professional previously telling the participant that they had coronary heart disease, heart failure, myocardial infarction or stroke.
Crothers et al 2023[15]	MI: centrally adjudicated MI events available at the time of these analyses.
Curkendal et al 2006[16]	HF, MI, Stroke: ICD-9 Codes
de Lucas-Ramos et al 2012[17]	Stroke, HF, PAD: Specifically designed questionnaire

de Miguel-Diez et al 2017[18]	HF, CKD, PAD: The criteria for diseases and procedures were defined according to the International Classification of Diseases-Ninth Revision, Clinical Modification (ICD-9-CM)
Dokollari et al 2024[19]	CKD, MI, Stroke, AF: NR - from notes review ; all-cause mortality: NA
Durheim et al 2018[20]	cardiovascular mortality, MI, HF: NR
Efird et al 2016[21]	MI, Stroke: NR
Ferreira et al 2023[22]	MI: new Q waves, new persistent ST-segment or T-wave changes, or elevated troponin levels; cardiovascular mortality: an unexplained death without an identifiable non-cardiac cause
Finkelstein et al 2009[23]	MI, PAD, Stroke: subjects were asked whether a doctor or other health professional told them that they had congestive heart failure, poor circulation in legs, stroke, coronary heart disease, angina (angina pectoris), irregular heartbeats, or heart attack (myocardial infarction)
FL Pavarino 2024[24]	Stroke: NR
Gaisl et al 2015[25]	Stroke: MACE were critically reviewed by two investigators (TG, CS) unaware of the participants allocation.
Grymonprez et al. 2019[26]	AF: Three methods were used for the assessment of prevalent and incident AF. Firstly, 10s ECGs with 12 leads and stored digitally, were taken at baseline and during follow-up. All ECGs were analyzed. To verify the diagnosis, all ECGs were independently reviewed by two research physicians. In case of a persisting disagreement between the coding physicians, a cardiologist made the final diagnosis.
Gulea et al 2019[27]	HF hospitalisation: CCS code 108 or ICD-9-CM: 4280, 428.1, 428.20, 428.21<br>428.22, 428.23, 428.30, 428.31, 428.32, 428.33<br>428.40, 428.41, 428.42, 428.43
Gupta et al 2022	MI:ICD-9-CM and ICD-10-CM
Hadi et al 2010[28]	HF, Stroke: NR; MI: All patients had a final diagnosisof ACS, including unstable angina, non–ST-segment elevation myocardial infarction (NSTEMI)and ST-segment elevation myocardial infarction (STEMI). Diagnosis of the different types of ACS and definitions of data variables were based on the American College of Cardiology (ACC) clinical data standards.
Hawkins et al 2009[29]	cardiovascular mortality: sudden cardiac death, fatal MI, and fatal worsening HF; HF hospitalisation: Hospitalization for HF was defined as admission with symptoms or signs of HF requiring intravenous treatment with diuretic, inotropic, or vasodilator therapy
Houben-Wilke et al. 2016[30]	PAD: PAD was objectively diagnosed using the ABI, which represents the ratio of ankle systolic pressure to brachial systolic pressure. A cutpoint of an ABI of 0.90 or less was used to define PAD
Huang et al 2014[31]	cardiovascular mortality: NR

Huang et al 2019[32]	cardiovascular mortality: NR; MI, Stroke: Adjudicated events; others are site reported.
Ingebrigtsen et al 2019[33]	HF hospitalisation: ICD-8: 427.09–427.11 and ICD-10: I50
Joo et al 2012[34]	CKD: Self-reported questionnaire
Kasemsap et al 2022[35]	MI: ICD-10 myocardial infarction (I21)
Keller et al 2018[36]	MI: ICD-10-CM: I21; NR
Kim et al 2017[37]	CKD: eGFR <60 mL/min/1.73 m2
Konecny et al 2018[38]	VT: VT was defined as a run of beats arising from the ventricles with duration of at least 3 beats and with cycle length ≤600 ms (equivalent to ≥100 beats per minute).Frequent VT was defined as >3 separate episodes during the Holter recording.
Kong et al. 2020[39]	VT: VT was defined as occurrence of more than 3 sequential PVCs, with heart rate >100 beats/minute
Kroenke et al 1993[40]	HF, VT: NR
Kurmi et al 2018[41]	CKD: ICD 10 (N02-N03, N07, N11, N18); Stroke: ICD 10 (I63, I61); MI: ICD 10 (Non-fatal MI [I21-23] or fatal IHD [I20-25]); HF: ICD 10 (I50); cardiovascular mortality: ICD 10 (I00-I99)
Lange et al 2010[42]	atrial fibrillation: NR
Lash et al 2011[43]	AF, HF, PAD, MI, CKD: ICD-10-CM
Laurisden et al 2023[44]	HF: ICD-10-CM: I50, I110, I42, J819, I130, I132; PAD: ICD-10-CM: I70-I74, I77; CKD: ICD-10-CM: N03-04, N11, N14-19, N26-27, I12-13, Z992, Q611-614, R34
Li et al 2024[45]	CKD and PAD: Elixhauser comorbidity
Li et al 2024[46]	MI: ICD-10-CM: I97.710, I97.711, I97.120, I97.121, I46.9; Stroke: ICD-10-CM: I60.9, I61.9, I63.22, I63.139, I63.239, I63.019, I63.119, I63.219, I97.811, I97.820, I97.821, I97.810
Liao et al 2019[47]	Stroke: NR
Lash et al 2011[43]	atrial fibrillation and PAD: ICD-9-CM codes from the outpatient and inpatient records
Manen et al 2001[48]	Stroke and CKD: self reported questionnaire
Manganas et al 2007[49]	atrial fibrillation: NR
Michalopoulos et al 2001[50]	MI and Stroke: NR
Miniati et al 2014[51]	HF: Chronic heart failure was recorded if, on transthoracic echocardiography, the left ventricular ejection fraction was ≤40% in at least 2 consecutive studies obtained in the year preceding the study entry; CKD, AF, Stroke: Clinical assessment included detailed

	clinical history and physical examination. In searching for comorbidities, pertinent laboratory tests and/or clinical charts of prior hospitalizations were reviewed; cardiovascular mortality: NR
Nagorni-Obradovic et al 2014[52]	MI, Stroke, CKD: self reported questionnaire
Noh et al 2024[53]	cardiovascular mortality: Outcomes related to mortality (all-cause and CV mortality) were defined as a recording of a death code within the NHID database and a CV cause of death (I00-I99)
Osman et al 2020[54]	MI: ICD9/10: 410, I21, 410.7 & 121A; Stroke: ICD9/10: 997.0, 997.00, 997.01, 997.02, I97.810, I97.811, I97.82, I97.820, I97.821
Parker et al 2014[55]	cardiovascular mortality: Mortality due to CVD was classified by ICD-10 codes: 120.0-125.9, 160–178 (ischemic stroke).
Perticone et al 2021[56]	MI: MI diagnosis was defined according to criteria of the European Society of Cardiology/American College of Cardiology Foundation/American Heart Association/World Heart Federation; Stroke: Stroke was defined as a new neurological deficit of sudden onset that persisted for at least 24 h
Poulsen et al 2015[57]	cardiovascular mortality: NR; Stroke: chart review
Proietti et al 2016[58]	cardiovascular mortality: NR
Qadeer et al 2021[59]	VT: NR
Raparelli et al 2018[60]	MI: definition proposed by the Joint European Society of Cardiology/American College of Cardiology Foundation/American Heart Association/World Heart Federation (ESC/ACCF/AHA/WHF) Task Force; Stroke: clinical manifestations and confirmed by radiological findings; CV mortality: outcome including vascular death(cardiac decompensation, heart failure, sudden or unwitnessed death, dysrhythmia, venous thromboembolism, procedure related death and presumed cardiovascular deaths) , fatal MI and fatal stroke
Rezaei et al 2018[61]	cardiovascular mortality: NR
Rivinius et al 2018[62]	atrial fibrillation: NR
Rodriguez et al 2011[63]	HF, MI, cardiovascular mortality: NR
Rodriguez et al 2010[64]	Stroke: CIAP2 – Classification of Primary Care - Ictus or acute transient ischemia symptoms: K89 – Transient cerebral ischemia; K90 – Stroke/cerebrovascular accident; K91 – cerebrovascular disease;
Rusnak et al 2024[65]	VT:duration of >30 s or causing hemodynamic collapse within 30 s, non-sustained VT by duration <30 s both with wide QRS complex (≥120 ms) at a rate greater than 100 beats per minute
Saleh et al 2012[66]	MI, AF, Stroke: NR

Schneider et al 2010[67]	MI, Stroke: NR
Serban et al 2017[68]	atrial fibrillation: new onset atrial fibrillation in-hospital
Shen et al 2022[69]	Stroke: ICD-9 code 430–434, 436, 438
Shibata et al 2013[70]	cardiovascular mortality: NR
Sidney et al 2005[71]	MI: ICD-9 code 410.x; ICD-10 codes I21.x to I22.x; Stroke: ; ICD-9 codes 431.x to 434.x, and 436.0; ICD-10 codes I60.x, I61.x, I63.x, and I64.x; HF: ICD-9 codes 428.x and 402.x1; ICD-10 codes I50.x; atrial fibrillation: ICD-9 codes 427.31 and 427.32; ICD-10 codes I48.0 and I48.1; cardiovascular mortality: NR
Skajaa et al 2023[72]	HF, PAD, Stroke, CKD, MI: one primary diagnosis and optional secondary diagnoses are recorded according to the International Classification of Diseases (ICD, 8th Revision between 1977 and 1993 (ICD-8) and 10th Revision (ICD-10) thereafter).
Sode et al. 2011[73]	MI: ICD8 410; ICD10 I21
Solderholm et al 2015[74]	Stroke: ICD-9 codes 430, 431, 434 and 436, and ICD 10-codes I60, I61, I63, I64
Song et al 2017[75]	cardiovascular mortality: NR
Soumagne et al 2020[76]	Stroke, PAD, AF: self-reported questionnaire
Ställberg et al 2018[77]	HF, MI, Stroke: diagnoses (according to the International Classification of Diseases, tenth revision [ICD-10] codes), spirometry measurements, laboratory tests, healthcare professional (HCP) visits, and referrals.
Sung et al 2012[78]	MI: STEMI was defined as (1) typical chest pain lasting for more than 30 minutes with ST-segment elevation > 1 mm in two consecutive precordial or inferior leads, (2) typical chest pain lasting for more than 30 minutes with a new-onset complete left bundle branch block.; HF hospitalisation: re-admission for congestive heart failure; defined as congestive heart failure≥New York heart association functional Class III
Szylinska et al 2020[79]	Stroke, AF: NR
Szylińska et al 2022[80]	HF, AF, MI, CKD: chart review
Takabayashi et al 2021[81]	HF hospitalisation: NR; Stroke: NR
Tavazzi et al 2013[82]	cardiovascular mortality and HF hospitalisation: endpoints were adjudicated by an independent endpoint validation committee.
Terzikhan et al 2018[83]	PAD: ABI of 0.90 or less in at least one leg

Tomaniak et al 2020[84]	MI: according to the Third Universal Myocardial Infarction definition, applicable at the time of study conduct, as study specific myocardial infarction criteria ; Stroke: acute onset of focal or global neurological deficit persisting ≥24 hours or <24 hours in case i) therapeutic intervention was required ii) it was confirmed by neuro-imaging or iii) patient's death
Tomioka et al. 2019[85]	HF: Framingham criteria. patients were diagnosed with CHF if they had two major findings or one major and two minor findings according to the criteria. Probable CHF and questionable CHF were not included. Cardiac death was defined as death due to CHF or sudden death.; cardiovascular mortality: death due to CHF or sudden death.
Wakabayashi et al 2010[86]	cardiovascular mortality: all deaths in which a noncardiac cause could not be demonstrated or a neurologic event including stroke or a transient ischemic attack
Yakubek et al 2018[87]	cardiovascular mortality, MI, Stroke: NR
Yang et al 2020[88]	MI, Stroke, CKD: defined by the ACS NSQIP
Yao et al 2022[89]	HF, Stroke: self reported physician diagnoses; CKD: eGFR< 60 mL/min/1.73m2, a urine albumin-creatinine ratio ≥ 30 mg/g, or a self-reported physician diagnosis of CKD.
Yakubek et al 2018[87]	MI: MI was defined in accordance with the Third Universal Definition from the American Heart Association; cardiovascular mortality: deaths attributed to a cardiac etiology were considered cardiogenic deaths; Stroke: Stroke, including ischemic stroke and bleeding stroke, was diagnosed based on neurological symptoms and imaging data
Yoshizawa et al 2015[90]	CKD: eGFR less than 60 mL/min/1.73 m2
Zafiraki et al 2021[91]	cardiovascular mortality, Stroke, MI : NR
Zhang et al 2013[92]	MI: NR

ACS – acute coronary syndrome; ACS NSQIP - American College of Surgeons' National Surgical Quality Improvement Program; ABI – ankle brachial index; AF – Atrial fibrillation; CHF – congestive heart failure; CKD – chronic kidney disease; CCS – clinical classification software; ECG – electrocardiogram; eGFR – estimated glomerular filtration rate; HF – heart failure; ICD – international classification of disease; MACE – major adverse cardiovascular events; MI – myocardial infarction; NR – not reported; NSTEMI – Non ST elevation myocardial infarction; OPCS - Office of Population Censuses and Surveys Classification of Interventions and Procedures; PAD – peripheral arterial disease; STEMI – ST Elevation Myocardial Infarction; VT – ventricular tachycardia;

Supplementary Table 4: Characteristics of Excluded Studies

Author name	Location	Reason for exclusion
Al-Khatib et al 2002[93]	United States	Insufficient event number data provided
Andell et al 2015[94]	Multinational (800 sites in 43 countries)	Insufficient event number data provided
Angeli et al 2019[95]	Italy	Insufficient event number data provided
Baty et al 2013[96]	Switzerland	Insufficient event number data provided
Bursi et al 2011[97]	United States	Insufficient event number data provided
Castagna et al 2007[98]	France	Insufficient event number data provided
Cave et al 2021[99]	Canada	Insufficient event number data provided
Chen et al 2021[100]	Taiwan	Insufficient event number data provided
Cohen et al 1995[101]	Israel	Insufficient event number data provided
Cuthbert et al 2018[102]	United Kingdom	Insufficient event number data provided
De Luca et al 2022[103]	Global	Insufficient event number data provided
de Miguel-Diez et al 2019[104]	Spain	Insufficient event number data provided
de Miguel-Diez et al 2019[105]	Spain	Insufficient event number data provided
de Miguel-Diez et al 2021[106]	Spain	Insufficient event number data provided
Distelmaier et al 2013[107]	Austria	Insufficient event number data provided
Eriksson et al 2018[108]	Sweden	Insufficient event number data provided

Feary et al 2010[109]	UK	Insufficient event number data provided
Funk et al 2013[110]	Austria	Insufficient event number data provided
Garcia-Olmos et al. 2013[111]	Spain	Insufficient event number data provided
Groenewegen et al 2022[112]	Netherlands	Insufficient event number data provided
Hong et al 2019[113]	Canada	Insufficient event number data provided
Kang et al 2024[114]	South Korea	Insufficient event number data provided
Lazzeri et al 2013[115]	Italy	Insufficient event number data provided
Liao and Lu 2016[116]	Taiwan	Insufficient event number data provided
Lim et al 2018[117]	South Korea	Insufficient event number data provided
Makris et al 2011[118]	France	Insufficient event number data provided
Mapel et al. 2009[119]	United States	Insufficient event number data provided
Navaneethan et al 2016[120]	USA	Insufficient event number data provided
Nilsson et al 2017[121]	Denmark	Insufficient event number data provided
Nishiyama et al 2010[122]	Japan	Insufficient event number data provided
Omote et al 2017[123]	South Korea	Insufficient event number data provided
Perez-Padilla et al 2018[124]	Chile, Brazil, Mexico, Uruguay, Venezuela	Insufficient event number data provided
Portegeis et al 2015[125]	Netherlands	Insufficient event number data provided
Putcha et al 2014[126]	USA	Insufficient event number data provided

Raslan et al 2023[127]	United Kingdom	Insufficient event number data provided
Restrepo et al 2006[128]	USA	Insufficient event number data provided
Sankar et al 2023[129]	Canada	Insufficient event number data provided
Scrutinio et al 2018[130]	Italy	Insufficient event number data provided
Soriano et al 2005[131]	United Kingdom	Insufficient event number data provided
Stefan et al 2012[132]	USA	Insufficient event number data provided
van Gester et al 2009[133]	Netherlands	Insufficient event number data provided
Vogelmeier et al 2023[134]	Germany	Insufficient event number data provided
Yang et al 2021[135]	China	Insufficient event number data provided

Supplementary Table 5: Characteristics of Included Studies

Author, year	Location	Control (n)	COPD (n)	Mean Control Age (y)	Mean COPD Age (y)	Males (Control) (%)	Males (COPD) (%)	Follow up duration
Agarwal et al 2017[1]	USA	1840517	279488	64·5	69·0	65·0	60·4	NR
Andell et al 2017[2]	Sweden	13045	1164	72·0	75·0	72·3	61·6	NR
Angouras et al 2010[3]	USA	3210	550	64·0	65·2	68·8	70·7	0·1
Arnaudis et al 2012[136]	France	216	132	56·5	65·2	75·0	78·8	4·6
Behar et al 1992[4]	Israel	5433	406	62·7	66·8	73·5	79·3	5·5
Bucci et al 2024[5]	Multinational	3982	112	68·3	75·8	64·7	87·5	1·0
Buklioska-Ilievska et al 2019[6]	Macedonia	30	60	65·2	66·2	76·7	86·7	NR
Buzea et al 2016[7]	Romania	64	47	61·7	64·4	48·4	68·1	NR
Campo et al 2013[8]	Italy	9086	2032	65·0	70·0	74·0	66·0	2·0
Canepa et al 2017[9]	Italy	13673	2656	68·6	70·8	61·5	69·0	NR
Carter et al· 2019[10]	UK	158230	31646	70·0	70·3	51·0	51·0	5·2
Chen et al 2016[11]	Taiwan	15478	7739	71·7	71·7	67·5	67·4	NR
Chiang et al 2022[12]	Taiwan	23704	23704	NR	NR	73·9	73·9	12·0
Chiu et al 2021[137]	Taiwan	2767	1219	65·4	73·1	55·4	80·1	NR
Chopard et al· 2014[13]	France, Monaco	3033	895	83·2	81·4	49·0	56·0	1·0
Christensen et al 2008[138]	Denmark	436	3630	NR	NR	45·3	41·7	0·1
Cobb et al 2024[14]	USA	31560	2504	56·2	62·4	49·0	43·2	20·0
Crothers et al 2023[15]	USA	11101	488	43·0	53·0	83·0	67·0	4·2
Curkendal et al 2006[16]	Saskatchewan	11493	22986	NR	NR	NR	NR	NR
Dai et al 2020[139]	China	20507	1575	NR	NR	58·4	55·4	NR
De Blois et al 2010[140]	Norway	3433	699	70·2	71·2	71·0	70·0	1·1
de Lucas-Ramos et al 2012[17]	Spain	278	970	59·0	64·1	73·7	70·4	NR
de Luise et al 2007[141]	Denmark	11214	771	80·2	77·4	27·9	38·5	1·8
de Miguel-Diez et al 2019[142]	Spain	1550	591	81·1	79·8	53·1	44·3	NR
de Miguel-Diez et al 2017[18]	Spain	601423	299713	75·4	75·9	53·3	75·7	0·1
de Miguel-Diez et al 2021[143]	Spain	157306	14949	74·7	76·5	52·4	67·6	0·1
Dokollari et al 2024[19]	USA	754	754	71·4	71·6	68·0	70·7	15·0
Durheim et al 2018[20]	Norway	8144	1605	75·0	76·0	57·0	59·6	NR
Efird et al 2016[21]	USA	3817	984	67·7	64·5	71·3	72·4	4·4
Ferreira et al 2023[22]	Brazil	44	73	58·0	62·0	50·7	59·1	6·0
Finkelstein et al 2009[23]	USA	17384	958	NR	60·3	NR	45·5	NR
Fisher et al 2015[144]	USA	6248	3500	76·5	75·6	43·6	44·6	NR
FL Pavarino 2024[24]	United States	206	181	72·0	72·0	77·0	65·0	1·8

Gaisl et al 2015[25]	Switzerland	81	81	64·3	64·3	77·8	77·8	1·8
Galizia et al 2010[145]	Italy	799	489	73·7	74·9	35·8	54·8	12·0
Grymonprez et al· 2019[26]	Netherlands	9574	1369	62·2	69·6	40·4	55·3	6·0
Gulea et al 2019[27]	USA	170207	54953	77·0	76·0	49·0	53·0	0·3
Guo et al 2021[146]	Taiwan	207199	14720	42·4	47·5	50·6	78·4	3·0
Gupta et al 2022[147]	USA	9986	360	50·2	66·5	39·2	30·0	0·1
Hadi et al 2010[28]	Abu Dhabi	7733	434	55·0	64·0	NR	NR	0·1
Hawkins et al 2009[29]	Multinational	13445	1258	64·5	68·1	68·7	71·1	NR
He et al 2020[148]	China	308	28	62·0	71·0	57·5	85·7	0·3
Hochhausen et al 2024[149]	Germany	3172662	291797	70·0	72·0	64·7	64·3	NR
Houben-Wilke et al· 2016[30]	Germany	1708	2088	65·3	65·0	61·1	65·3	NR
Huang et al 2020[150]	China	26167	3332	62·9	71·7	57·2	54·5	0·1
Huang et al 2014[31]	China	1748	227	67·5	75·7	44·6	50·2	1·0
Huang et al 2019[32]	USA	675	135	67·3	67·4	70·8	71·1	0·1
Ingebrigtsen et al 2019[33]	Denmark	42058	7968	58·0	66·3	39·0	57·0	5·7
Jacob et al 2016[151]	Spain	6030	2069	79·9	79·7	36·9	63·3	NR
Joo et al 2012[34]	South Korea	673	238	54·4	64·6	36·9	67·2	NR
Kasemsap et al 2022[35]	Thailand	12650	12650	75·0	75·0	78·8	79·3	5·0
Keller et al 2018[36]	Germany	4850816	761011	NR	NR	63·7	71·8	0·1
Kim et al 2017[37]	South Korea	2499	528	NR	NR	43·5	76·7	NR
Konecny et al 2018[38]	USA	3551	2800	61·0	71·0	43·2	63·8	NR
Kong et al· 2020[39]	China	45	151	63·1	68·8	62·2	72·7	NR
Kroenke et al 1993[40]	USA	52	78	61·0	64·3	65·0	52·8	0·1
Kurmi et al 2018[41]	China	456143	33239	50·9	54·0	40·7	44·3	9·0
Lacedonia et al 2021[152]	Italy	435	70	78·2	65·3	52·9	41·4	0·1
Lai et al 2018[153]	Taiwan	1820	1820	71·5	71·6	62·9	62·8	10·0
Lange et al 2010[42]	Denmark	4854	1036	57·2	67·4	41·8	47·9	13·0
Lange et al 2016[154]	Denmark	2199	320	56	65	32	66	22
Lash et al 2011[43]	Denmark	322495	64499	NR	NR	46·0	46·0	5·0
Laurisden et al 2023[44]	Denmark	6403	644	NR	NR	55·8	54·3	1·0
Li et al 2024[45]	USA	3581	701	NR	NR	67·8	57·2	0·1
Li et al 2024[46]	USA	440	92	NR	NR	55·2	53·5	0·1
Liao et al 2019[47]	Taiwan	51869	51869	64·3	64·3	55·2	56·3	8·0
Liapikou et al 2012[155]	Spain	1167	212	69·4	73·4	54·4	90·1	0·1
Lim et al 2020[156]	South Korea	194	83	61·8	72·0	48·5	71·1	5·0
Lindberg et al 2012[157]	Sweden	993	993	NR	NR	54·5	54·7	4·0
Londono etal 2017[158]	Spain	773	212	78·4	78·4	38·7	69·3	1·0
Manen et al 2001[48]	Netherlands	421	290	65·9	65·8	41·1	64·1	NR
Manganas et al 2007[49]	Canada	101	221	67·5	68·1	76·2	77·8	0·1
Marott et al 2020[159]	Denmark	1026	144	33·0	33·0	50·0	54·2	18·0

Méndez-Bailón et al 2017[160]	Spain	35897	35897	NR	NR	NR	NR	10·0
Michalopoulos et al 2001[50]	Greece	136	136	62·9	63·5	93·0	93·0	NR
Miniati et al 2014[51]	Italy	201	200	65·0	66·0	86·0	89·0	NR
Myagmardorj et al 2023[161]	Netherlands	272	128	79·0	76·6	52·0	69·5	10·0
Nagorni-Obradovic et al 2014[52]	Serbia	9360	653	59·3	62·8	54·4	46·6	NR
Nogimura et al 2022[162]	Japan	5231	1431	NR	NR	100·0	100·0	3·0
Noh et al 2024[53]	Korea	4931472	170338	NR	NR	47·9	47·0	5·0
Opina et al 2019[163]	USA	300	296	77·1	77·5	58·7	67·2	13·0
Osman et al 2020[54]	USA	2455	2495	76·2	75·8	53·2	51·7	NR
Parissis et al 2014[164]	Multinational	3473	1143	69·0	74·0	59·5	65·7	NR
Parker et al 2014[55]	USA	2107	1100	50·9	62·2	47·9	57·0	NR
Perticone et al 2021[56]	Italy	1204	524	59·0	68·0	56·3	70·2	5·6
Ponomarev et al 2018[165]	Finland	423	31	63·0	NR	82·2	NR	1·0
Poulsen et al 2015[57]	Denmark	94	37	82·0	79·6	50·0	66·0	1·5
Proietti et al 2016[58]	Europe	2747	339	68·4	72·5	59·0	64·6	1·0
Purdue et al 2006[166]	Sweden	162466	6849	NR	NR	NR	NR	NR
Qadeer et al 2021[59]	USA	431	162	66·3	69·4	64·7	52·5	NR
Quan et al 2021[167]	USA	11957	467	NR	NR	26·1	12·8	0·1
Raparelli et al 2018[60]	Italy	1842	185	73·1	76·2	54·0	62·0	3·0
Rello et al 2006[168]	Spain	252	176	57·1	67·1	63·9	85·2	0·2
Rezaei et al 2018[61]	Austria	18648	16275	NR	NR	63·0	61·6	NR
Rivinius et al 2018[62]	Germany	196	63	51·2	53·6	74·5	77·8	5·0
Rodriguez et al 2011[63]	Spain	175	60	53·4	68·5	73·1	85·0	0·3
Rodriguez et al 2010[64]	United Kingdom	15238	1627	NR	NR	NR	NR	5·0
Rodríguez-Mañero et al 2019[169]	China	7053	937	76·5	82·0	46·8	67·9	1·9
Romiti et al 2024[170]	multinational	24291	1569	NR	NR	NR	NR	3·0
Rothnie et al 2015[171]	United Kingdom	266142	34019	NR	NR	67·1	61·9	0·5
Rusnak et al 2024[65]	Germany	2556	257	68·0	72·0	70·0	75·0	NR
Saleh et al 2012[66]	UK	9417	265	65·3	69·2	80·5	80·7	NR
Salisbury et al 2007[172]	Sweden	2094	387	60·0	64·5	68·5	61·8	NR
Schneider et al 2010[67]	United Kingdom	35722	35722	NR	NR	51·3	51·3	NR
Serban et al 2017[68]	Romania	387	31	61·8	66·7	69·5	77·4	NR
Selvaraj et al 2005[173]	USA	9877	1117	64·1	67·6	71·1	62·0	2·8
Semenzato et al 2021[174]	Spain	209	302	52·0	62·0	70·0	91·0	6·0
Shen et al 2022[69]	Taiwan	24170	24173	NR	NR	59·1	59·1	NR
Shibata et al 2013[70]	China	2915	338	NR	NR	NR	NR	7·0
Sidney et al 2005[71]	USA	45966	45966	NR	NR	55·4	55·4	3·0

Skajaa et al 2023[72]	Denmark	428917	142973	NR	NR	49·7	49·7	3·0
Sode et al· 2011[73]	Denmark	7105833	313958	26·0	55·0	50·0	55·0	27·0
Solderholm et al 2015[74]	Sweden	103419	103419	69·9	69·9	54·0	54·0	NR
Song et al 2017[75]	South Korea	483650	11771	47·4	64·1	49·6	47·5	4·0
Soumagne et al 2020[76]	France	1555	266	58·4	56·7	56·0	70·7	NR
Ställberg et al 2018[77]	Sweden	84514	17479	NR	NR	NR	NR	NR
Stone et al 2012[175]	USA	2238	1217	72·0	72·3	81·0	72·9	5·0
Su et al 2017[176]	Taiwan	4849	1921	67·0	77·0	68·5	1306·0	NR
Sung et al 2012[78]	Taiwan	1430	124	60·9	68·5	81·2	85·5	NR
Szylinska et al 2020[79]	Poland	2980	198	65·7	68·6	71·1	65·2	NR
Szylińska et al 2022[80]	Poland	906	95	72·1	71·6	50·8	66·3	1·0
Takabayashi et al 2021[81]	Japan	943	159	77·0	79·2	49·6	61·6	1·6
Tavazzi et al 2013[82]	International	5775	730	60·4	65·1	76·0	82·0	1·9
Terzikhan et al 2018[83]	Netherlands	3038	80	65·1	65·7	42·6	52·9	16·5
Tomaniak et al 2020[84]	International	15136	832	64·4	67·9	76·8	75·5	2·0
Tomioka et al· 2019[85]	Japan	149	190	65·5	77·5	63·1	84·7	8·8
Vaes et al 2014[177]	Denmark	8734	1270	48·5	56·2	42·4	57·9	17·1
Wakabayashi et al 2010[86]	USA	2884	365	60·7	66·2	68·5	59·1	NR
Wang et al 2021[178]	China	1646	154	64·9	66·9	78·1	73·4	10·0
Wu et al[179]	Taiwan	51471	6664	na	na	33.1	63.07	1.0
Xiao et al 2020[180]	China	5937	2529	NR	NR	51·1	55·6	NR
Yakubek et al 2018[87]	USA	107193	3975	66·8	68·7	37·0	39·0	0·1
Yang et al 2020[88]	USA	11437	1800	45·1	58·5	48·3	63·7	NR
Yao et al 2022[89]	China	9614	229	58·3	65·4	77·2	74·7	NR
Yoshizawa et al 2015[90]	Japan	73	108	71·8	74·3	49·3	83·3	NR
Zafiraki et al 2021[91]	Russia	392	254	57·7	59·4	94·4	95·3	1·8
Zhang et al 2022[181]	China	51	10	52·8	76·0	41·2	100·0	4·2
Zhang et al 2021[182]	Taiwan	1546	773	58·4	58·8	0·0	0·0	10·0
Zhang et al 2021[183]	Taiwan	644	322	72·0	71·5	68·2	69·3	10·0
Zhang et al 2013[92]	China	4510	645	64·7	68·4	71·0	73·0	NR

COPD – chronic obstructive pulmonary disease; NR – not reported; y – years; USA – United States of America; UK – United Kingdom;

Supplementary Table 6: Sensitivity and subgroup analysis for Myocardial Infarction

**Strata**	**Number of Cohorts**	**RR (95% CI)**	**I^2 (%)**
**Age**			
<75	25	1·44 (1·17–1·76)	98·5
≥75	4	1·28 (0·97–1·70)	69·2
**Cohort type**			
Prospective	15	1·44 (1·20–1·72)	96·5
Retrospective	24	1·48 (1·22–1·81)	99·4
**Context**			
ACS	9	1·65 (1·18–2·31)	88·6
AF	2	1·48 (1·10–1·98)	0·0
General	17	1·57 (1·30–1·90)	99·8
Stroke	2	1·17 (0·93-1·46)	0·0
Surgery	8	1·15 (0·88–1·50)	47·0
TAVI	1	0·66 (0·34–1·30)	NA
**Continent**			
Asia	8	1·42 (1·01–2·00)	
Europe	15	1·35 (1·12–1·64)	99·7
Multiple	1	1·16 (0·81–1·65)	NA
North America	14	1·83 (1·54–2·17)	88·0
South America	1	0·25 (0·09–0·67)	NA
**Follow Up**			
<6 months	7	1·23 (0·83–1·83)	86·6
≥ 6 months	20	1·44 (1·22–1·70)	99·3
**Bias**			
High risk of bias removed	21	1·51 (1·24–1·83)	99·7
**Publication Year**			
Before 2010	4	1·83 (1·70–1·96)	0·0
After 2010	35	1·42 (1·22–1·66)	99·6
**Small sample sizes removed**			
Lower tertile removed	28	1·41 (1·23–1·62)	99·5
**Estimate type**			
Adjusted	20	1·38 (1·19-1·61)	99·4
Crude	18	1·51 (1·17-1·96)	98·8
**COPD ascertainment**			
Spirometry	14	1·40 (1·02 - 1·92)	95·6
Code	13	1·40 (1·17 - 1·66)	99·7
Clinical	9	1·60 (1·18 - 2·17)	90·0
**Risk group**			
High	20	1·35 (1·08 - 1·68)	83.0
Low	19	1·57 (1·33 - 1·86)	99.7

Abbreviations: ACS – acute coronary syndrome; AF – atrial fibrillation; CABG – coronary artery bypass grafting; OPD – chronic obstructive pulmonary disease; HF – heart failure; ICU – intensive care unit; TAVI – transcatheter aortic valve implantation

Supplementary Table 7: Sensitivity and subgroup analysis for Heart Failure

Strata	Number of Cohorts	RR (95% CI)	I^2 (%)
**Age**			
<75	13	2·46 (1·62–3·74)	99·9
≥75	2	1·18 (1·17–1·19)	0·0
**Cohort type**			
Prospective	4	3·67 (1·24–10·83)	99·8
Retrospective	19	2·10 (1·69–2·61)	99·9
**Context**			
ACS	3	1·54 (1·34–1·78)	88·9
AF	3	2·69 (0·91–7·97)	87·2
General	13	2·74 (1·90–3·95)	100·0
Stroke	1	1·68 (0·90–3·15)	NA
Surgery	1	1·67 (0·75–3·70)	NA
TAVI	1	1·30 (1·18–1·43)	NA
**Continent**			
Asia	4	2·05 (1·19–3·55)	98·9
Europe	11	2·30 (1·45–3·65)	100·0
Multiple	1	3·24 (1·52–6·87)	NA
North America	6	2·44 (1·64–3·62)	99·8
**Follow Up**			
<6 months	3	1·41 (1·06–1·87)	94·3
≥ 6 months	12	2·43 (1·89–3·13)	99·8
**Bias**			
High risk of bias removed	12	2·14 (1·66–2·75)	99·9
**Publication Year**			
Before 2010	4	2·29 (1·37–3·82)	98·4
After 2010	18	2·35 (1·70–3·22)	100·0
**Small sample sizes removed**			
Lower tertile removed	17	2·08 (1·70–2·56)	99·9
**Estimate type**			
Adjusted	9	2·26 (1·80-·2·83)	99·5
Crude	11	2·61 (1·58-4·29)	100·0
**COPD ascertainment**			
Spirometry	8	2·82 (1·47 - 5·40)	99·7
Code	9	2·17 (1·74 - 2·71)	99·9
Clinical	1	4·20 (4·08 - 4·32)	NA
**Risk group**			
High	6	1·49 (1·32 - 1·68)	86·0
Low	16	2·72 (1·94 - 3·82)	100·0

Abbreviations: ACS – acute coronary syndrome; AF – atrial fibrillation; CABG – coronary artery bypass grafting; OPD – chronic obstructive pulmonary disease; HF – heart failure; ICU – intensive care unit; TAVI – transcatheter aortic valve implantation

Supplementary Table 8: Sensitivity and subgroup analysis for Heart Failure Hospitalisation

Strata	Number of Cohorts	RR (95% CI)	I^2 (%)
**Age**			
<75	7	1·80 (1·18-2·74)	99·4
≥75	2	1·75 (1·21–2·52)	88·3
**Cohort type**			
Prospective	6	1·31 (1·20–1·44)	62·2
Retrospective	3	3·25 (2·07–5·10)	99·7
**Context**			
ACS	3	1·95 (0·89–4·29)	99·4
General	1	3·87 (3·77-3·98)	NA
Heart Failure	5	1·45 (1·18–1·78)	92·2
**Continent**			
Asia	2	2·49 (0·83–7·42)	98·2
Europe	4	1·74 (1·02–2·97)	99·6
Multiple	2	1·22 (1·10–1·34)	1·4
North America	1	2·07 (1·99-2·14)	NA
**Follow Up**			
<6 months	1	2·07 (1·99–2·14)	NA
≥ 6 months	4	1·81 (1·09–3·01)	99·5
**Bias**			
High risk of bias removed	9	1·78 (1·28–2·47)	99·5
**Publication Year**			
Before 2010	1	1·17 (1·04–1·32)	NA
After 2010	8	1·88 (1·31–2·67)	99·6
**Small sample sizes removed**			
Lower tertile removed	7	1·62 (1·17–2·24)	99·5
**Estimate type**			
Adjusted	6	1·39 (1·16-1·68)	92·8
Crude	3	2·91 (1·49 - 5·69)	99·7
**COPD ascertainment**			
Spirometry	3	2·90 (1·46 - 5·76)	98·8
Code	2	1·75 (1·25 - 2·43)	98·9
Clinical	4	1·23 (1·15 - 1·33)	0·0
**Risk group**			
High	8	1·61 (1·19 - 2·18)	98·8
Low	1	3·87 (3·77 - 3·98)	NA

Abbreviations: ACS – acute coronary syndrome; AF – atrial fibrillation; CABG – coronary artery bypass grafting; OPD – chronic obstructive pulmonary disease; HF – heart failure; ICU – intensive care unit; TAVI – transcatheter aortic valve implantation

Supplementary Table 9: Sensitivity and subgroup analysis for Atrial Fibrillation

Strata	Number of Cohorts	RR (95% CI)	I^2 (%)
**Age**			
<75	15	1·30 (1·12–1·51)	94·3
**Cohort type**			
Prospective	3	1·41 (1·20–1·80)	2·0
Retrospective	14	1·38 (1·16–1·64)	99·1
**Context**			
ACS	2	1·62 (1·43–1·84)	0·0
CABG	1	1·21 (0·95–1·53)	NA
General	8	1·41 (1·13–1·75)	99·4
Stroke	1	4·52 (1·33–15·35)	NA
Surgery	5	1·28 (0·95–1·72)	88·5
**Continent**			
Asia	2	1·23 (0·71–2·11)	98·4
Europe	11	1·43 (1·21–1·69)	98·5
North America	3	1·36 (0·86–2·15)	94·2
**Follow Up**			
<6 months	1	0·98 (0·70–1·37)	NA
≥ 6 months	10	1·53 (1·26–1·85)	99·3
**Bias**			
High risk of bias removed	10	1·43 (1·17–1·73)	99·3
**Publication Year**			
Before 2010	3	1·53 (1·00–2·33)	94·1
After 2010	14	1·134 (1·14–1·56)	98·6
**Small sample sizes removed**			
Lower tertile removed	11	1·36 (1·16–1·60)	99·0
**Estimate type**			
Adjusted	7	1·42 (1·09-1·85)	83·3
Crude	9	1·37, 1·10-1·71)	97·5
**COPD ascertainment**			
Spirometry	9	1·42 (1·12 - 1·79)	84·0
Code	6	1·25 (1·03 - 1·53)	99·3
**Risk group**			
High	9	1·36 (1·10 - 1·69)	84·5
Low	8	1·41 (1·13 - 1·75)	99·4

Abbreviations: ACS – acute coronary syndrome; AF – atrial fibrillation; CABG – coronary artery bypass grafting; OPD – chronic obstructive pulmonary disease; HF – heart failure; ICU – intensive care unit; TAVI – transcatheter aortic valve implantation

Supplementary Table 10: Sensitivity and subgroup analysis for Ventricular Tachycardia

Strata	Number of Cohorts	RR (95% CI)	I^2 (%)
**Age**			
<75	4	2·05 (1·27–3·31)	84·6
**Cohort type**			
Prospective	2	1·98 (1·39–2·81)	0·0
Retrospective	2	2·12 (0·76–5·96)	95·3
**Context**			
Surgery	1	0·83 (0·46–1·48)	NA
General	3	1·60 (1·12–2·27)	61·3
**Continent**			
Asia	1	2·27 (1·07–4·83)	NA
Europe	1	1·91 (1·28–2·83)	NA
North America	2	2·12 (0·76–5·96)	95·3
**Publication Year**			
Before 2010	1	3·67 (2·40–5·60)	NA
After 2010	3	1·60 (1·12–2·27)	61·3
**Estimate type**			
Adjusted	1	1·28 (1·10-1·48)	NA
Crude	2	2·63 (1·39-5·00)	79·5
**COPD ascertainment**			
Spirometry	4	2·05 (1·27 - 3·31)	84·6
**Risk group**			
High	1	3·67 (2·40 - 5·60)	NA
Low	3	1·60 (1·12 - 2·27)_	61·3

Abbreviations: ACS – acute coronary syndrome; AF – atrial fibrillation; CABG – coronary artery bypass grafting; OPD – chronic obstructive pulmonary disease; HF – heart failure; ICU – intensive care unit; TAVI – transcatheter aortic valve implantation

Supplementary Table 11: Sensitivity and subgroup analysis for Stroke

Strata	Number of Cohorts	RR (95% CI)	I^2 (%)
**Age**			
<75	29	1·41 (1·12–1·79)	98·0
≥75	4	0·99 (0·71-1·38)	0·0
**Cohort type**			
Prospective	19	1·38 (1·06–1·79)	90·5
Retrospective	24	1·38 (1·13–1·69)	99·3
**Context**			
ACS	8	1·19 (0·86–1·63)	33·3
AF	2	1·12 (0·72–1·75)	0·0
CABG	1	0·58 (0·28–1·22)	NA
General	19	1·58 (1·25–2·00)	99·6
Surgery	11	1·31 (0·93–1·85)	71·7
TAVI	2	0·89 (0·55–1·46)	0·0
**Continent**			
Asia	7	1·30 (0·87–1·93)	94·7
Europe	20	1·35 (1·02–1·78)	99·5
Multiple	1	2·07 (1·26-3·40)	NA
North America	15	1·45 (1·15-1·81)	94·1
**Follow Up**			
<6 months	7	1·10 (0·82–1·49)	48·6
≥ 6 months	18	1·49 (1·13–1·98)	99·0
**Bias**			
High risk of bias removed	25	1·29 (1·04–1·61)	99·2
**Publication Year**			
Before 2010	4	1·30 (1·14–1·47)	48·8
After 2010	36	1·39 (1·17–1·66)	99·1
**Small sample sizes removed**			
Lower tertile removed	28	1·32 (1·13–1·54)	99·0
**Estimate type**			
Adjusted	17	1·38 (1·09-1·74)	98·7
Crude	26	1·38 (1·11-1·73)	98·6
**COPD ascertainment**			
Spirometry	20	1·46 (1·07 - 1·99)	96·3
Code	12	1·21 (0·96 - 1·52)	99·4
Clinical	9	1·55 (1·19 - 2·03)	67·3
**Risk group**			
High	22	1·20 (0·95 - 1·51)	66·8
Low	21	1·54 (1·24 - 1·92)	99·5

Abbreviations: ACS – acute coronary syndrome; AF – atrial fibrillation; CABG – coronary artery bypass grafting; OPD – chronic obstructive pulmonary disease; HF – heart failure; ICU – intensive care unit; TAVI – transcatheter aortic valve implantation

Supplementary Table 12: Sensitivity and subgroup analysis for Peripheral Arterial Disease

Strata	Number of Cohorts	RR (95% CI)	I^2 (%)
**Age**			
<75	7	2·26 (1·34–3·82)	99·5
>75	1	1·54 (1·52–1·56)	NA
**Cohort type**			
Prospective	5	2·89 (1·60–5·19)	97·1
Retrospective	8	1·59 (1·30–1·94)	99·7
**Context**			
General	11	2·14 (1·54–2·96)	99·9
Surgery	1	1·12 (1·03–1·23)	NA
TAVI	1	1·68 (1·51-1·87)	NA
**Continent**			
Europe	10	2·25 (1·62–3·13)	99·9
Asia	1	1·07 (1·03–1·12)	NA
North America	2	1·53 (0·82–2·85)	96·8
**Follow Up**			
<6 months	2	1·32 (0·97–1·79)	97·7
≥ 6 months	6	1·71 (1·37–2·14)	99·4
**Bias**			
High risk of bias removed	9	1·90 (1·30–2·78)	99·9
**Publication Year**			
Before 2010	1	2·12 (1·73–2·59)	NA
After 2010	12	1·98 (1·44–2·71)	99·9
**Small sample sizes removed**			
Lower tertile removed	11	1·91 (1·38–2·63)	99·9
**Estimate type**			
Adjusted	3	2·10 (2·04-2·16)	0·0
Crude	9	2·02 (1·32-3·09)	99·9
**COPD ascertainment**			
Spirometry	6	2·57 (1·48 - 4·46)	98·1
Code	6	1·58 (1·24 - 2·01)	99·8
Clinical	1	2·12 (1·73 - 2·59)	NA
**Risk group**			
High	2	1·37 (0·93 - 2·03)	96·7
Low	11	2·14 (1·54 - 2·96)	99·9

Abbreviations: ACS – acute coronary syndrome; AF – atrial fibrillation; CABG – coronary artery bypass grafting; OPD – chronic obstructive pulmonary disease; HF – heart failure; ICU – intensive care unit; TAVI – transcatheter aortic valve implantation

Supplementary Table 13: Sensitivity and subgroup analysis for Chronic Kidney Disease

Strata	Number of Cohorts	RR (95% CI)	I^2 (%)
**Age**			
<75	13	1·64 (1·17–2·29)	98·1
>75	1	1·01 (1·00–1·02)	NA
**Cohort type**			
Prospective	5	1·81 (0·88–3·75)	98·3
Retrospective	14	1·60 (1·20–2·15)	99·7
**Context**			
General	13	1·61 (1·20–2·16)	99·7
Stroke	1	4·77 (3·81–5·97)	NA
Surgery	4	1·16 (0·58–2·35)	73
TAVI	1	1·68 (1·45–1·93)	NA
**Continent**			
Asia	5	1·88 (0·96–3·68)	99·2
Europe	9	1·83 (1·29–2·60)	99·8
North America	5	1·16 (0·73–1·84)	75·8
**Follow Up**			
<6 months	4	1·05 (0·54–2·04)	80·1
≥ 6 months	7	1·74 (1·16–2·60)	99·7
**Bias**			
High risk of bias removed	11	1·51 (1·09–2·09)	99·8
**Publication Year**			
Before 2010	1	1·45 (0·09–23·12)	NA
After 2010	18	1·65 (1·25–2·17)	99·7
**Small sample sizes removed**			
Lower tertile removed	13	1·49 (1·14–1·96)	99·7
**Estimate type**			
Adjusted	7	1·42 (1·08-1·87)	98.1
Crude	11	1·89 (1·19-2·99)	99.7
**COPD ascertainment**			
Spirometry	9	1·67 (1·02 - 2·75)	98.0
Code	7	1·37 (1·03 - 1·83)	99·7
Clinical	2	1·83 (1·46 - 2·29)	0·0
**Risk group**			
High	6	1·65 (0·87 - 3·14)	94·5
Low	13	1·61 (1·20 - 2·16)	99·7

Abbreviations: ACS – acute coronary syndrome; AF – atrial fibrillation; CABG – coronary artery bypass grafting; OPD – chronic obstructive pulmonary disease; HF – heart failure; ICU – intensive care unit; TAVI – transcatheter aortic valve implantation

Supplementary Table 14: Sensitivity and subgroup analysis for Cardiovascular Mortality

Strata	Number of Cohorts	RR (95% CI)	I^2 (%)
**Age**			
<75	24	1·60 (1·33–1·92)	94·6
≥75	4	1·32 (1·10–1·50)	8·3
**Cohort type**			
Prospective	21	1·53 (1·27–1·84)	93·7
Retrospective	13	1·59 (1·18–2·14)	99·7
**Context**			
ACS	9	1·75 (1·26–2·42)	94·7
AF	6	1·94 (1·45–2·60)	50·9
CABG	1	0·95 (0·87–1·04)	NA
General	12	1·57 (1·18–2·10)	99·6
HF	3	1·10 (0·96–1·28)	32·2
TAVI	3	1·24 (1·07–1·43)	0·0
**Continent**			
Asia	11	1·53 (1·12-2·11)	99·8
Europe	13	1·47 (1·19–1·81)	94·0
Multiple	3	1·03 (0·93–1·15)	0·0
North America	7	2·03 (1·43–2·89)	89·2
**Follow Up**			
≥ 6 months	22	1·66 (1·34–2·06)	99·6
**Bias**			
High risk of bias removed	26	1·51 (1·25–1·82)	99·3
**Publication Year**			
Before 2010	2	1·47 (0·74–2·93)	98·8
After 2010	32	1·56 (1·32–1·84)	99·3
**Small sample sizes removed**			
Lower tertile removed	23	1·39 (1·17–1·65)	99·4
**Estimate type**			
Adjusted	16	1·31 (1·07-1·61)	98·7
Crude	16	1·92 (1·52-2·42)	96·2
**COPD ascertainment**			
Spirometry	15	1·65 (1·29 - 2·10)	95.1
Code	6	1·43 (0·93 - 2·21)	99.9
Clinical	11	1·50 (1·15 - 1·96)	91·7
**Risk group**			
High	16	1·40 (1·14 - 1·73)	95·9
Low	18	1·70 (1·35 - 2·13)	99·3

Abbreviations: ACS – acute coronary syndrome; AF – atrial fibrillation; CABG – coronary artery bypass grafting; OPD – chronic obstructive pulmonary disease; HF – heart failure; ICU – intensive care unit; TAVI – transcatheter aortic valve implantation

Supplementary Table 15: Sensitivity and subgroup analysis for Cardiovascular Mortality < 1 year

Strata	Number of Cohorts	RR (95% CI)	I^2 (%)
**Age**			
<75	2	3·83 (2·78–5·27)	0·0
≥75	2	1·25 (1·00–1·57)	0·0
**Cohort type**			
Prospective	3	2·32 (1·09–4·93)	90·7
Retrospective	2	1·07 (0·99–1·17)	0·0
**Context**			
ACS	2	3·83 (2·78–5·27)	0·0
General	1	1·08 (0·99–1·17)	NA
TAVI	2	1·25 (1·00–1·57)	0·0
**Continent**			
Asia	1	2·63 (0·99–7·01)	NA
Europe	3	1·12 (0·98–1·28)	24·4
North America	1	4·00 (2·85–5·61)	NA
**Follow Up**			
≥ 6 months	4	2·02 (0·97–4·21)	87·4
**Bias**			
High risk of bias removed	4	1·56 (0·74–3·29)	96·7
**Publication Year**			
After 2010	5	1·71 (0·92–3·18)	94·8
**Small sample sizes removed**			
Lowest tertile	2	1·13 (0·98–1·30)	42·4
**Estimate type**			
Crude	4	2·02 (0·97-4·21)	87·4
**COPD ascertainment**			
Spirometry	1	0·51 (0·06 - 4·20)	NA
Code	1	1·08 (0·99 - 1·17)	NA
Clinical	3	2·32 (1·09 - 4·93)	90.7
**Risk group**			
High	4	2·02 (0·97 - 4·21)	87·4
Low	1	1·08 (0·99 - 1·17)	NA

Abbreviations: ACS – acute coronary syndrome; AF – atrial fibrillation; CABG – coronary artery bypass grafting; OPD – chronic obstructive pulmonary disease; HF – heart failure; ICU – intensive care unit; TAVI – transcatheter aortic valve implantation

Supplementary Table 16: Sensitivity and subgroup analysis for Cardiovascular Mortality >1 year

Strata	Number of Cohorts	RR (95% CI)	I^2 (%)
**Age**			
<75	4	1·48 (0·93–2·35)	80·1
≥75	1	1·23 (1·02–1·48)	NA
**Cohort type**			
Prospective	4	1·56 (1·14–2·13)	53·1
Retrospective	3	1·98 –(0·96–4·09)	99·7
**Context**			
ACS	1	2·18 (1·35–3·52)	NA
AF	2	1·62 (1·10–2·38)	0·0
General	2	2·93 (2·90–2·97)	0·0
CABG	1	0·95 (0·87–1·04)	NA
TAVI	1	1·23 (1·02–1·48)	NA
**Continent**			
Asia	3	2·25 (1·52–3·31)	78·6
Europe	3	1·49 (0·78–2·83)	98·8
Multiple	1	2·64 (0·60–11·64)	NA
**Follow Up**			
≥ 6 months	6	2·08 (1·49–2·91)	96·1
**Bias**			
High risk of bias removed	7	1·82 (1·26–2·63)	98·4
**Publication Year**			
After 2010	7	1·82 (1·26–2·63)	98·4
**Small sample sizes removed**			
Lowest tertile	5	1·82 (1·09–3·05)	99·2
**Estimate type**			
Adjusted	2	1·62 (1·10-2·38)	0·0
Crude	5	1·83 (1·16-2·90)	99·1
**COPD ascertainment**			
Spirometry	1	1·56 (1·05 - 2·33)	NA
Code	3	1·98 (0·96 - 4·09)	99.7
Clinical	2	1·57 (0·90 - 2·73)	79.0
**Risk group**			
High	3	1·30 (0·84 - 2·01)	93·1
Low	4	2·48 (1·82 - 3·40)	93·8

Abbreviations: ACS – acute coronary syndrome; AF – atrial fibrillation; CABG – coronary artery bypass grafting; OPD – chronic obstructive pulmonary disease; HF – heart failure; ICU – intensive care unit; TAVI – transcatheter aortic valve implantation

Supplementary Table 17: Sensitivity and subgroup analysis for All-cause Mortality

Strata	Number of Cohorts	RR (95% CI)	I^2 (%)
**Age**			
<75	79	1·65 (1·47–1·86)	99·0
≥75	23	1·21 (1·04–1·41)	99·0
**Cohort type**			
Retrospective	77	1·50 (1·32–1·70)	99·9
Prospective	48	1·65 (1·47–1·85)	98·9
**Context**			
ACS	19	1·59 (1·34–1·89)	99·2
AF	9	1·76 (1·53–2·01)	66·4
CABG	2	1·53 (1·27–1·83)	0·0
Cancer	4	1·34 (0·95–1·88)	93·8
General	33	1·81 (1·43–2·28)	100·0
HF	14	1·05 (0·96–1·14)	82·3
ICU	5	1·24 (1·14–1·48)	53·6
PCI	1	1·34 (1·21–1·48)	NA
Stroke	5	1·18 (1·14–1·23)	39·5
Surgery	26	1·73 (1·42–2·09)	93·3
TAVI	6	1·22 (1·07–1·40)	29·3
**Continent**			
Asia	29	1·60 (1·26–2·04)	99·7
Europe	60	1·52 (1·35–1·70)	99·7
Multiple	7	1·57 (1·11–2·22)	96·7
North America	28	1·60 (1·34–1·91)	98·8
**Follow Up**			
≥ 6 months	21	1·45 (1·13–1·85)	99·7
< 6 months	66	1·73 (1·52–1·98)	99·7
**Bias**			
High risk of bias removed	82	1·49 (1·36–1·63)	99·8
**Publication Year**			
Before 2010	12	1·80 (1·36–2·39)	98·0
After 2010	112	1·53 (1·39–1·68)	99·8
**Small sample sizes removed**			
Lowest tertile	78	1·41 (1·30–1·54)	99·8
**Estimate type**			
Adjusted	62	1·38 (1·29-1·49)	98·0
Crude	52	1·70 (1·42-2·05)	99·9
**COPD ascertainment**			
Spirometry	46	1·87 (1·55 - 2·25)	99·0
Code	48	1·27 (1·15 - 1·40)	99·8
Clinical	21	1·57 (1·33 - 1·85)	96·8
**Risk group**			
High	78	1·41 (1·30 - 1·54)	98·3
Low	47	1·79 (1·50 - 2·14)	99·9

Abbreviations: ACS – acute coronary syndrome; AF – atrial fibrillation; CABG – coronary artery bypass grafting; OPD – chronic obstructive pulmonary disease; HF – heart failure; ICU – intensive care unit; TAVI – transcatheter aortic valve implantation

Supplementary Table 18: Sensitivity and subgroup analysis for All-cause Mortality <1 year

Strata	Number of Cohorts	RR (95% CI)	I^2 (%)
**Age**			
<75	25	1·42 (1·17–1·73)	98·9
≥75	10	1·14 (0·83–1·55)	99·8
**Cohort type**			
Retrospective	33	1·26 (1·08–1·46)	99·7
Prospective	11	1·61 (1·20–2·15)	98·7
**Context**			
ACS	7	1·56 (1·09–2·23)	99·8
AF	1	1·83 (1·64–2·05)	NA
CABG	1	1·78 (1·17–2·72)	NA
General	9	1·26 (0·83–1·91)	99·9
HF	5	0·92 (0·83–1·02)	79·3
ICU	3	1·20 (1·11–1·30)	54·5
Stroke	3	1·15 (1·11–1·19)	0·0
Surgery	15	1·66 (1·24–2·24)	93·2
TAVI	1	1·24 (0·97–1·58)	NA
**Continent**			
Asia	6	1·21 (1·15–1·27)	2·7
Europe	21	1·30 (1·07–1·59)	99·8
Multiple	2	1·31 (0·67–2·57)	96·7
North America	18	1·39 (1·05–1·85)	99·7
**Follow Up**			
≥ 6 months	13	1·61 (1·23–2·12)	98·0
< 6 months	15	1·36 (1·02–1·80)	99·8
**Bias**			
High risk of bias removed	33	1·37 (1·17–1·61)	99·8
**Publication Year**			
Before 2010	4	1·53 (1·24–1·88)	64·5
After 2010	40	1·34 (1·15–1·55)	99·8
**Small sample sizes removed**			
Lowest tertile	26	1·26 (1·10–1·45)	99·7
**Estimate type**			
Adjusted	20	1·26 (1·12 - 1·43)	99·3
Crude	20	1·35 (1·04 – 1·75)	99·7
**COPD ascertainment**			
Spirometry	10	1·46 (1·20-1·77)	50·3
Code	24	1·11 (0·99-1·24)	99·5
Clinical	7	1·89 (1·29 - 2·78)	99·0
**Risk group**			
High	34	1·35 (1·17 - 1·56)	99·4
Low	10	1·32 (0·90 - 1·92)	99·9

Abbreviations: ACS – acute coronary syndrome; AF – atrial fibrillation; CABG – coronary artery bypass grafting; OPD – chronic obstructive pulmonary disease; HF – heart failure; ICU – intensive care unit; TAVI – transcatheter aortic valve implantation

Supplementary Table 19: Sensitivity and subgroup analysis for All-cause Mortality over 1 year

Strata	Number of Cohorts	RR (95% CI)	I^2 (%)
**Age**			
<75	16	1·99 (1·38–2·87)	98·8
≥75	7	1·22 (1·08–1·37)	94·9
**Cohort type**			
Retrospective	22	1·90 (1·42–2·52)	99·9
Prospective	8	1·47 (1·24–1·74)	94·3
**Context**			
ACS	4	1·48 (1·07–2·05)	77·4
AF	3	1·86 (1·16–3·00)	87·4
CABG	1	1·47 (1·21–1·80)	NA
Cancer	2	1·58 (0·83–33·01)	96·0
General	5	3·48 (1·37–8·86)	100·0
HF	4	1·15 (0·99–1·33)	89·1
PCI	1	1·34 (1·21–1·48)	NA
Stroke	2	1·32 (0·95–1·84)	43·4
Surgery	6	1·92 (1·44–2·56)	92·8
TAVI	2	1·33 (1·01–1·75)	72·0
**Continent**			
Asia	11	1·9 (1·14 - ·3·20)	99·9
Europe	14	1·73 (1·41–2·12)	96·5
Multiple	2	2·21 (1·01–4·84)	86·7
North America	3	1·32 (1·09–1·58)	95·8
**Follow Up**			
≥ 6 months	20	2·11 (1·59–2·81)	99·9
**Bias**			
High risk of bias removed	21	1·67 (1·42–1·97)	99·7
**Publication Year**			
Before 2010	3	1·51 (1·18–1·92)	83·5
After 2010	27	1·84 (1·44–2·33)	99·9
**Small sample sizes removed**			
Lowest tertile	17	1·52 (1·28–1·80)	99·8
**Estimate type**			
Adjusted	15	1·46 (1·28-1·67)	93·6
Crude	11	2·10 (1.24 – 3·55)	99·9
**COPD ascertainment**			
Spirometry	8	2·75 (1·40 - 5·38)	98·7
Code	13	1·57 (1·27 - 1·93)	99·8
Clinical	5	1·36 (1·14 - 1·62)	92·8
**Risk group**			
High	19	1·43 (1·27 - 1·61)	95·5
Low	11	2·38 (1·46 - 3·87)	100·0

Abbreviations: ACS – acute coronary syndrome; AF – atrial fibrillation; CABG – coronary artery bypass grafting; OPD – chronic obstructive pulmonary disease; HF – heart failure; ICU – intensive care unit; TAVI – transcatheter aortic valve implantation

Supplementary Table 20: COPD ascertainment for each study

doi	Author	COPD Definition	Definition Group
10.1016/j.amjcard.2017.02.024	Agarwal et al	ICD9	code
10.1093/ejcts/ezx219	Andell et al	COPD status was also ascertained from the National Drug Dispensary registry, where dispensed prescriptions of certain medications for COPD [Anatomical Therapeutic Chemical codes: R03AK (Adrenergics in combination with corticosteroids or other drugs, excluding anticholinergics), R03BB01 (Ipratropium bromide) or R03BB04 (Tiotropium bromide)] in the previous 6 months before the event indicated COPD	code
10.1016/j.athoracsur.2010.01.009	Angouras et al	Patients who required chronic (>3 months) bronchodilator therapy to avoid disability from obstructive airway disease, had a forced expiratory volume in 1 second (FEV1) less than 75% of the predicted value or less than 1.25 L, or had room air partial pressure of oxygen less than 60 mm Hg or partial pressure of carbon dioxide greater than 50 mm Hg were considered COPD patients	spirometry
10.1007/s00392-012-0450-4	Arnaudis et al	COPD was defined by a post-bronchodilatator FEV1/FVC ratio <0.7. When FEV1/FVC ratio was between 0.68 and 0.72 spirometry was analysed by an expert pneumologist (RE) which categorized the patient as COPD or no COPD.	spirometry
10.1016/0002-9343(92)90196-i	Behar et al	na	na
10.1161/JAHA.123.032785	Bucci et al	na	na
10.3889/oamjms.2019.576	Buklioska-Ilievska et al	GOLD criteria	spirometry
10.1097/MJT.0000000000000450	Buzea et al	GOLD criteria	spirometry
10.1378/chest.12-2313	Campo et al	A patient was considered to have COPD combining three different sources of data: (1) COPD marked as comorbidity in the REAL dataset, (2) documented history of hospital admission for COPD, and (3) treatment with pharmacologic therapies specific for COPD (eg, inhaled steroids, inhaled anticholinergics, inhaled b -agonists, or theophylline)	code
10.1002/ejhf.964	Canepa et al	clinical judgment of each investigator, taking into account the patient's medical history, treatment and/or spirometric data	clinical
10.1016/j.jacc.2018.11.063	Carter et al.	ICD10	code
10.1038/srep25855.	Chen et al	ICD9	code
10.1097/JCMA.0000000000000835.	Chiang et al	ICD9	code
10.3390/cancers13133231	Chiu et al	ICD9	code

j.amjcard.2014.01.432	Chopard et al.	clinical definition as the presence of lung symptoms (e.g., progressive dyspnea, cough, wheezing, or sputum production) requiring long-term use of bronchodilators or steroids	clinical
10.1378/chest.07-2543	Christensen et al	ICD10	code
10.1111/resp.14799	Cobb et al	self reported diagnosis by healthcare professional	clinical
10.1097/QAD.0000000000003465	Crothers et al	OPD International Classification of Disease (ICD) diagnosis codes and ≥90-day continuous supply of COPD short-acting (short-acting beta-agonist or muscarinic antagonists) or long-acting controller inhaler medications (long-acting beta-agonist, long-acting muscarinic antagonist, or inhaled corticosteroid).	code
10.1016/j.annepidem.2005.04.008	Curkendal l et al	ICD9	code
10.2147/COPD.S275573	Dai et al	ICD9	code
10.1016/j.cardfail.2009.12.002	De Blois et al	COPD status was adjudicated by reviewing the data collected from the patients' files regarding the presence of obstructive lung disease.	clinical
10.2147/COPD.S36222	de Lucas-Ramos et al	COPD defined by the presence of a postbronchodilator forced expiratory volume in 1 second/forced vital capacity ratio < 0.7.	spirometry
10.1007/s10654-007-9211-5	de Luise et al	ICD8	code
10.1002/ccd.28321	de Miguel-Diez et al	ICD9	code
10.1016/j.ejim.2017.06.008	de Miguel-Diez et al	ICD9	code
10.2147/COPD.S311826	de Miguel-Diez et al	ICD10	code
10.14503/THIJ-23-8199	Dokollari et al	Patients with COPD were defined as those with a documented medical history of COPD, regardless of the duration of disease or the need for treatment	code
10.1136/heartjnl-2017-312735	Durheim et al	na	na
10.4037/ajcc2016119	Efird et al	COPD was classified preoperatively on the basis of severity by using the following Society of Thoracic Surgeons (STS) database criteria; "Mild: forced expiratory volume in 1 second (FEV1) 60% to 75% of predicted, and/or on chronic inhaled or oral bronchodilator therapy; Moderate: FEV1 50% to 59% of predicted, and/or on chronic steroid therapy aimed at lung disease; and Severe: FEV1 50."	spirometry
10.2147/COPD.S410416	Ferreira et al	The diagnosis of COPD was established by spirometry, which was performed for all participants according to American Thoracic Society performance criteria.20 A post-bronchodilator ratio of forced expiratory volume in 1s (FEV1) to forced vital capacity (FVC) of less than 70% of the	spirometry

		predicted value confirmed the presence of persistent airflow limitation, which was compatible with COPD diagnosis	
10.2147/copd.s6400	Finkelstein et al	COPD status was ascertained based on the following questions: “Have you ever been told by a doctor or other health professional that you had emphysema?” and “During the past 12 months, have you been told by a doctor or other health professional that you had chronic bronchitis?” Subjects’ reports of what they were told by physicians may not necessarily correspond to the strict medical definition of COPD (eg, GOLD criteria). To take this into account we analyzed the data using three different definitions of “COPD” of increasing strictness: 1) Subjects report having “chronic bronchitis” and/or “emphysema”; 2) Same as #1 and are □40 years old; 3. Same as #2 and report smoking more than 100 cigarettes in their lifetime	clinical
10.1378/chest.14-0607	Fisher et al	COPD was considered to be present if a patient was described in his or her medical record as having clinical or radiographic evidence of COPD.	clinical
10.1007/s00270-024-03884-w	FL Pavarino	Patients with history of COPD were stratified in accordance with the Global Initiative for Chronic Obstructive Lung Disease (GOLD) criteria	spirometry
10.1371/journal.pone.0126613	Gaisl et al	COPD was staged according to Global Initiative for Chronic Obstructive Lung Disease [GOLD]-guidelines 2007	spirometry
10.1007/BF03351076	Galizia et al	The diagnosis of COPD was considered possible when: a) participants reported that they had been told by a physician that they had COPD, and/or b) they had received specific treatment with beta-agonists or steroids. The diagnosis was subsequently confirmed by means of a physical examination and a review of spirometry records. In particular, subjects were considered COPD if they presented values of forced expiratory volume in 1 second/forced vital capacity (FVE1/FVC)	spirometry
10.1016/j.ijcard.2018.09.056	Grymonprez et al.	COPD was diagnosed by an obstructive spirometry at the research center, or, in absence of an interpretable spirometry at the research center, by a physician based on clinical history, physical examination or spirometry performed outside the investigations of the research center	spirometry
10.1016/j.ijcard.2019.04.087	Gulea et al	ICD9	code
10.1016/j.rmed.2021.106471	Guo et al	The COPD classifications were defined according to the criteria developed by Global Initiative for Chronic Obstructive Lung Disease (GOLD) [21]: non-COPD (FEV1/FVC ≥ 0.7), GOLD Ⅰ (or mild; FEV1/FVC <0.7 and FEV1 ≥ 80% predicted), GOLD Ⅱ (or moderate; FEV1/FVC <0.7 and 50% ≤ FEV1 < 80% predicted), GOLD Ⅲ (or severe; FEV1/FVC <0.7 and 30% ≤ FEV1 < 50% predicted), and GOLD Ⅳ (or very severe; FEV1/FVC <0.7 and FEV1 < 30% predicted).	spirometry

10.1053/j.jfas.2022.03.015	Gupta et al	For a patient to have a diagnosis of COPD, the medical record must document that there is a historical or current diagnosis of COPD and at least one of the following: functional disability from the COPD, chronic bronchodilator therapy, hospitalization for COPD, or a forced expiratory volume in the first second (FEV1) less than 75% of predicted on a pulmonary function test.	spirometry
10.1002/clc.20751	Hadi et al	any patient with a documented history of obstructive pulmonary disease (i.e., COPD or asthma) or treatment with pharmacologic therapies specific for obstructive lung disease (inhaled steroids, inhaled anticholinergics, inhaled β-agonists, or theophylline) were considered to have COPD	code
10.1093/eurjhf/hfp001	Hawkins et al	The presence of clinically recognized chronic obstructive pulmonary disease was recorded using a yes/no check box by individual site investigators at study entry according to their clinical judgement.	clinical
10.12659/MSM.927212	He et al	Patients with a previous history of COPD were defined as those with a long history of smoking, a diagnosis of COPD at our hospital, and treatment with inhaled drugs.	clinical
10.1038/s41598-024-56255-3	Hochhausen et al	ICD10	code
10.1164/rccm.201602-0354OC	Houben-Wilke et al.	Patients were classified according to the spirometric criteria of GOLD and the GOLD group A to D criteria	spirometry
10.2147/COPD.S244020	Huang et al	ICD9	code
10.1016/j.jamda.2014.04.009	Huang et al	Patients with COPD were defined as those with an obstructive deficit at pulmonary function testing [forced expiratory volume in 1 s (FEV1)/forced vital capacity ratio <70%	spirometry
10.1093/ejcts/ezy438	Huang et al	COPD at baseline was defined as a prior diagnosis of COPD and ongoing treatment with bronchodilators and/or oral or inhalational steroids.	clinical
10. 1136/<br><br>bmjresp- 2019- 000470	Ingebrigtsen et al	COPD with FEV1 ≥ 50% of predicted value: FEV1/FVC<0.70 with >10 pack-years of tobacco smoking, no self-reported asthma and FEV1 ≥50% of predicted value. COPD with FEV1 < 50% of predicted value: FEV1/FVC<0.70 with >10 pack-years of tobacco smoking, no self-reported asthma and FEV1<50% of predicted value	spirometry
10.1016/j.ijcard.2016.11.013	Jacob et al	ICD	code
10.3346/jkms.2012.27.8.901	Joo et al	Airflow obstruction was defined as a ratio of forced expiratory volume in 1 second (FEV1) divided by forced vital capacity (FVC) < 0.7, with COPD defined as airflow obstruction in persons aged 40 yr and older.	spirometry
10.1159/000521767	Kasemsap et al	ICD10	code
10.1016/j.rmed.2018.12.010	Keller et al	ICD10	code

10.1007/s11255-017-1572-4	Kim et al	COPD was defined as FEV1/FVC Patients were grouped for the follow up depending on the presence of COPD as shown by spirometry.	spirometry
10.3346/jkms.2023.38.e344	Kim et al	ICD10	code
10.1016/j.hrthm.2017.09.042	Konecny et al	Severity of COPD was classified in the following manner: 1) mild to moderate COPD defined as FEV1/FVC <0.7 and FEV1 % predicted equal or greater than 0.5. 2) FEV1/FVC<0.7 and 0.3≤FEV1 percent predicted<0.5; 3) very severe COPD defined as FEV1/FVC<0.7 and FEV1 percent predicted<0.3.	spirometry
10.2147/COPD.S234470	Kong et al.	All the included patients qualified the diagnostic criteria of COPD as proposed by the Global initiative for chronic Obstructive Lung Disease (GOLD) in 2013.	spirometry
10.1378/chest.104.5.1445	Kroenke et al	Using proposed criteria as a guide, we defined airways obstruction as follows: severe- FEV, <50% of predicted and FEV/FVC of <70%; mild-moderate and FEV1/FVD equal or greater than 70	spirometry
10.2147/COPD.S153641	Kurmi et al	We decided to use the general term "AFO" instead of "COPD", as all the results are based on prebronchodilator spirometry.	spirometry
10.1038/s41598-021-98749-4	Lacedonia et al	The anamnesis, obtained both directly from the patients (if clinical conditions allowed) or from the relatives and/or clinical records, was aimed to ascertain smoking habits, the coexistence of respiratory diseases (such as asthma and COPD) and other comorbidities (diabetes mellitus, neurological diseases, kidney diseases, heart diseases, and cancer history). We used a semi-structured questionnaire in all the sites involved, investigating the same comorbidities	clinical
10.2147/COPD.S174215	Lai et al	diagnosis of COPD was validated based on one inpatient or three outpatient records of International Classification of Diseases (ICD), ninth revision, Clinical Modification (ICD-9-CM) codes 491, 492, and 496 and the prescription of at least one bronchodilator during the follow-up period	code
10.1016/ S2213-2600(16)00098-9	Lange et al	According to the examination in 1991–94, we assigned participants into one of the following six subgroups on the basis of the information obtained from the questionnaire and results of spirometry. e defined severe exacerbations of obstructive lung disease (ICD-8 codes 491–93 and ICD-10 codes J41–46)	spirometry
10.3109/15412550903499506	Lange et al	The participants were classified according to COPD stage using the international GOLD staging according to FEV1 in % of predicted	spirometry
10.1007/s00408-010-9274-z	Lash et al	ICD10	code
10.1016/j.ahj.2023.04.016	Laurisden et al	GOLD classification	spirometry
10.1097/CORR.0000000000000531	Lee et al	na	na

10.1016/j.carrev.2024.06.013	Li et al	ICD10	code
10.1177/17085381241298732	Li et al	na	na
10.2147/COPD.S202029	Liao et al	ICD9	code
10.1183/09031936.00067111	Liapikou et al	The classification of COPD severity was based on the Global Initiative for Chronic Obstructive Lung Disease (GOLD) criteria: GOLD stage I was defined as a forced expiratory volume in 1 s (FEV1) .80% predicted, GOLD stage II as FEV1 between 50 and 80% pred, GOLD stage III as FEV1 between 30 and 50% pred and GOLD stage IV as FEV1 ,30% pre	spirometry
10.2147/COPD.S274354	Lim et al	According to the Global Initiative for Chronic Obstructive Lung Disease (GOLD) guidelines, post-bronchodilator fixed criteria [forced expiratory volume in 1 s divided by forced vital capacity (FEV1/FVC)<0.7] was applied for definition of COPD	spirometry
10.1186/1471-2466-12-1	Lindberg et al	According to the Global Initiative for Chronic Obstructive Lung Disease (GOLD) guidelines, post-bronchodilator fixed criteria [forced expiratory volume in 1 s divided by forced vital capacity (FEV1/FVC)<0.7] was applied for definition of COPD	spirometry
10.1007/s11739-018-1820-3	Londono etal	ICD9	code
10.1016/S0895-4356(01)00346-8	Manen et al	Only subjects with a FEV1/VC-ratio below the reference ratio minus 1.64 3 standard deviation before and after inhalation of 400 mg salbutamol were included (n 5 290). This way only subjects with a chronic obstruction of the airways were selected.	spirometry
10.1155/2007/378963	Manganas et al	According to the 2004 American Thoracic Society/European Respiratory Society position paper on the diagnosis and treatment of COPD, patients with COPD were categorized into two groups based of the degree of airflow obstruction: mild to moderate COPD with an FEV1 50% or more and FEV1/FVC 0.7 or less, and severe COPD with an FEV1 less than 50% and FEV1/FVC 0.7 or less	spirometry
10.1164/rccm.201911-2115OC	Marott et al	COPD was defined as a FEV1/forced vital capacity (FVC) < the lower limit of normal (LLN) and FEV1	spirometry
10.1016/j.ijcard.2017.02.017	Méndez-Bailón et al	ICD9	code
10.1007/s00268-001-0161-X	Michalopoulos et al	Mild, moderate, and severe COPD was determined by FEV 1% predicted values of 70% to 80%, 50% to 69%, and less than 50%, respectively	spirometry
10.1097/MD.0000000000000076	Miniati et al	The criteria for case recruitment were: firm clinical diagnosis of stable COPD; airflow obstruction as indicated by a postbronchodilator ratio of forced expiratory volume in 1 second (FEV1) over forced vital capacity (FVC)	spirometry
10.1016/j.amjcard.2022.11.007	Myagmardorj et al	Patients were divided according to the results of FEV1 and the categories defined in the Society of Thoracic Surgeons (STS) Adult Cardiac Surgery Database: normal pulmonary function was defined by an FEV1 >75% of predicted, mild COPD if FEV1	spirometry

		was 60% to 75% of predicted, moderate COPD if FEV1 was 50% to 59% of predicted, and severe COPD when FEV1 <50% of predicted.	
10.1038/npjpcrm.2014.8	Nagorni-Obradovic et al	The classification variable to identify respondents with COPD was a selfreported history of chronic bronchitis and emphysema as measured by the following question: 'Has a doctor ever diagnosed you to have chronic bronchitis or emphysema?' Participants who answered positively were considered to have COPD.	clinical
10.1016/j.archger.2022.104741	Nogimura et al	To identify those with COPD, we used a screening questionnaire named the International Primary Care Airway Group (IPAG) questionnaire, which includes questions about age, smoking, amounts (pack-years), Body Mass Index, allergies, and four questions about COPD symptoms (cough, phlegm, and wheeze)	clinical
10.2147/COPD.S458779	Noh et al	ICD10	code
10.1111/jgs.16052	Opina et al	FEV1:FVC ratio lower than .70 per the modified GOLD criteria	spirometry
10.1002/ccd.28913	Osman et al	ICD9/10	code
10.1007/s00392-014-0708-0	Parissis et al	COPD diagnosis was based on medical history and past or current medications related to COPD. All clinical diagnoses, patient-related variables and outcomes used standardized definitions.	code
10.1186/1471-2466-14-195	Parker et al	Diagnosis of COPD was based on spirometry. We used an FEV1/FVC ratio (forced expiratory volume in one second/forced vital capacity) of <0.70 to define airflow obstruction.	spirometry
10.1186/1471-2466-14-196	Parker et al	Diagnosis of COPD was based on spirometry. We used an FEV1/FVC ratio (forced expiratory volume in one second/forced vital capacity) of <0.70 to define airflow obstruction.	spirometry
10.1038/s41598-021-86963-z	Perticone et al	Diagnosis of COPD was carried out according to medical history and by a standard spirometry performed by and trained and experienced personnel. Particularly, the established criterion for COPD were a post-bronchodilator forced expiratory volume in 1 s (FEV1)/forced vital capacity (FVC) ratio <70%, and/or use of inhaled drugs, including long acting β2 adrenoceptor agonists (LABA) or/and muscarinic receptor antagonists (LAMA).	spirometry
10.1053/j.jvca.2017.12.016	Ponomarev. D et al	Obstructive pattern, also referred to as obstructive lung disease, was defined as FEV1/FVC <0.70; restrictive respiratory pattern was defined as a combination of FEV1/FVC ≥0.70 and FVC <80% of predicted; and mixed pattern was defined as a combination of FEV1/FVC <0.70, FEV1 <80% of predicted, and FVC <80% of predicted.	spirometry
10.3109/14017431.2015.1006671	Poulsen et al	Patients with COPD were diagnosed by either their general physician or pulmonologist prior to referral for cardiac evaluation using the standard criteria for COPD in Denmark (chronic, slowly progressive disorder characterized by airflow obstruction (FEV1 < 80% of predicted and FEV1/FVC ratio	spirometry

10.1016/j.ahj.2016.08.011	Proietti et al	The presence COPD was defined according to documented medical history, as collected by physicians at study site-level. This assessment was performed by any physician during the clinical interview with the patient and/or by searching through medical records, if available.	clinical
10.1136/thx.2006.064196	Purdue et al	Following the method of Mannino et al, 5 we classified individuals into five categories of lung function according to FEV1/VC and %FEV1: normal lung function (FEV1/ VC >70%, %FEV1 >80%), mild COPD (FEV1/VC ,70%, %FEV1 >80%), moderate COPD (FEV1/VC ,70%, %FEV1 50–79%), severe COPD (FEV1/VC ,70%, %FEV ,50%) and RLD (FEV1/VC>70%, %FEV1 ,80%).	spirometry
10.1371/journal.pone.0259698	Qadeer et al	Demographic and medical history recorded were all obtained from patient's electronic medical record	code
10.1007/s00590-021-03067-0	Quan et al	na	na
10.1007/s11739-018-1835-9	Raparelli et al	The diagnosis of COPD was reported by each investigator in the case report form at the time of enrollment. As for other risk factors, Investigators were asked to follow international guidelines to define COPD (GOLD).	spirometry
10.1183/09031936.06.00139305	Rello et al	The diagnosis of COPD was reported by each investigator in the case report form at the time of enrollment. As for other risk factors, Investigators were asked to follow international guidelines to define COPD (GOLD).	spirometry
10.1111/crj.12968	Rezaei et al	Among these patients, the co-diagnosis of COPD was identified by ICD-10 code J44 including sub-codes.	code
10.2147/CLEP.S171929	Rivinius et al	A Tiffeneau index (FEV1/FVC)	spirometry
10.1378/chest.10-2439	Rodriguez et al	COPD was defined as a disease state characterized by the presence of airflow limitation due to chronic bronchitis or emphysema. The airflow obstruction may be accompanied by airway hyperreactivity and may be partially reversible. The diagnosis of COPD was confirmed with premorbid pulmonary function testing when available. In the absence of documented airfl ow obstruction, we used clinical criteria, medical records with compatible physical findings, and evidence of hyperinflation on chest radiograph.	spirometry
10.1016/j.rmed.2010.04.018	Rodriguez et al	detection of suspected COPD using OXMIS and Read codes for obstructive lung diseases, chronic bronchitis and emphysema; Questionnaires were also sent to the patients'<br>PCPs to confirm the validity of the COPD diagnosis.	code
10.2147/COPD.S174443	Rodríguez-Mañero et al	All patients coded with COPD were successively verified by one investigator (ELP). COPD was defined by a FEV1 /FVC ratio ,0.70 after 400 µg of inhaled albuterol.	spirometry
10.1093/europace/euae021	Romiti et al	At baseline, investigators recorded data regarding demographics, comorbidities, and treatment prescribed for patients enrolled in the study, using	clinical

		standardized electronic case report forms. Amongst co-morbidities, investigators were able to report whether patients had respiratory diseases and specifically if patients had COPD; no additional information on the definition or severity of COPD was available.	
10.1136/heartjnl-2014-307251	Rothnie et al	In this subset of data, patients with COPD were identified using a combination of MINAP-recorded obstructive airway disease and a smoking history (ex or current smoker).	code
10.1016/j.rmed.2018.10.019	Rusnak et al	For the present analysis risk-stratification was performed according to the presence of COPD according to latest GOLD guidelines.	spirometry
10.1093/ejcts/ezr271	Saleh et al	Depending on the degree of airflow limitation, the study population was divided into three groups: Group 1 (including patients with normal spirometry and those with mild COPD: FEV1/FVC ratio < 70%, FEV1 ≥ 80% predicted), Group 2 (moderate COPD: FEV1/FVC ratio < 70%, 50% ≤ FEV1 < 80% predicted) and Group 3 (severe COPD: FEV1/ FVC ratio < 70%, FEV1 < 50% predicted	spirometry
10.1016/j.amjcard.2006.09.112	Salisbury et al	Instead, any patient with a documented history of obstructive pulmonary disease (i.e., COPD or asthma) or treatment with pharmacologic therapies specific for obstructive lung disease (e.g., inhaled steroids, inhaled anticholinergics, inhaled agonists or theophylline) were considered to have COPD.	code
10.1007/s10654-010-9435-7	Schneider et al	na	na
10.1016/j.ijcard.2017.05.044	Seban et al	patients were considered to have COPD if COPD diagnosis was reported in their medical records, or if they were treated with COPD-specific pharmacologic therapies (e.g., inhaled steroids, anticholinergics, theophylline agonists)	code
10.1016/j.amjcard.2005.05.016.	Selvaraj et al	The diagnosis of COPD was based on the clinical history or obtained from chart review and recorded as a co-morbidity in the database.	code
10.1159/000515180	Semenzato et al	COPD was defined by FEV1/FVC	spirometry
10.1038/s41533-021-00267-3	Shen et al	ICD9	code
10.1371/journal.pone.0083725	Shibata et al	An airflow obstruction was defined as FEV1 /FVC < 0.70, instead of FEV1 /VC < 5th percentile of the predicted value [20], because FEV1 /FVC < 0.7 is more common	spirometry
10.1378/chest.128.4.2068	Sidney et al	One hundred twenty records of COPD cohort members were randomly selected for medical record review (96 outpatient records; 24 hospitalization records). A medical record abstractor obtained and abstracted the Kaiser Permanente medical records for a 12-month period of time prior to and subsequent to the date of COPD diagnosis. We defined spirometrically determined categories of airflow as follows: normal; mild airflow obstruction (FEV1/FVC ratio,	spirometry

		70% predicted; FEV1, 80% predicted); or airway obstruction (FEV1/FVC ratio, 70% predicted; FEV1, 80% predicted) according to the Global Initiative for Chronic Obstructive Lung Disease criteria.13 Tobacco smoking, chronic cough, exertional dyspnea, asthma, chronic bronchitis, emphysema, and COPD medications were considered to be present if noted in the medical record during this time period. Medication was recorded if noted in the medical record, including inhaled anticholinergic agents, inhaled -adrenergic steroids, a combination of inhaled anticholinergic agents and -adrenergic agonists, and methylxanthine agents.	
10.1136/bmjresp-2023-001798	Skajaa et al	ICD9	code
10.1093/eurheartj/ehr338	Sode et al.	ICD10	code
10.1007/s10654-015-0113-7	Solderholm et al	ICD10	code
10.1016/j.amjcard.2017.07.032	Song et al	Patients with COPD were defined as subjects who met the following 2 criteria: (1) subjects with a diagnosis of ICD-10 codes for COPD or emphysema (J42, J43 [except for J43.0], and J44) and (2) subjects with a prescription of more than 1 of the following COPD drugs at least twice per year: long-acting muscarinic antagonists, long-acting beta-2 agonists, inhaled corticosteroids, short-acting muscarinic antagonists, short-acting beta-2 agonists, or methylxanthine (>1 month).	code
10.2147/COPD.S253417	Sougmagne et al	The criteria for retaining a diagnosis of mild-tomoderate COPD were (i) a history of exposure to risk factors (tobacco smoking and/or daily exposure to organic dusts) and (ii) evidence of persistent airflow limitation defined as a post-bronchodilator forced expiratory volume in 1 second (FEV1) on forced vital capacity (FVC) ratio 50% of predicted value according to the GLI-2012 equations.	spirometry
10.1038/s41533-018-0101-y	Ställberg et al	ICD10	code
10.1016/j.jvs.2012.11.132	Stone et al	na	na
10.1161/JAHA.116.004525	Su et al	ICD9	code
10.4103/2319-4170.113373	Sung et al	(1) Information on COPD status was obtained by reviewing chart record of the need for pharmacologic therapy using bronchodilator agent; (2) Past history of a 1-second forced expiratory volume < 70% of the predicted value (by pulmonary function test);[1] (3) Physical examination (by auscultation) showed expiratory wheezing and further confirmed by blood gas and chest radiograph (i.e., emphysematous change); or (4) Current use of bronchodilators prior to acute myocardial infarction (AMI).	spirometry
10.1371/journal.pone.0265682	Svendson et al.	The diagnosis of COPD was based on the Global Initiative for Chronic Obstructive Lung Disease (GOLD) guidelines and required both	spirometry

		spirometrically confirmed airflow obstruction, and a physician's assessment of the clinical history.	
10.1097/MD.0000000000019675	Szylinska et al	The first group included patients with a clinical diagnosis of COPD according to the GOLD criteria stages I to IV.	spirometry
10.3390/brainsci12010106	Szylińska et al	na	na
10.1007/s00380-020-01675-0	Takabayashi et al	COPD was defined in this study if it had been previously diagnosed by a physician, the data of the previous medical record, or the results of spirometry at each site.	spirometry
10.1016/j.ijcard.2013.10.068	Tavazzi et al	A diagnosis of COPD was recorded by the investigators at study enrolment, based on clinical evidence and past medical history of COPD.	clinical
10.1183/23120541.00086-2018	Terzikhan et al	We validated whether participants had COPD by reviewing the medical charts, including outpatient clinic reports and hospital discharge letters, of all those who used medication for obstructive lung disease for at least 6 months (anatomical therapeutic chemical (ATC) classification code R03) or who stated they had COPD in a questionnaire. COPD cases were defined as having a medical COPD diagnosis supported by clinical presentation and/or obstructive lung function	spirometry
10.1093/ehjcvp/pvz052	Tomaniak et al	COPD was defined as a chronic lung disease requiring a long-term use of bronchodilators or steroids, as defined in the EuroSCORE. It was diagnosed by investigators based on clinical examination and prior medical history of COPD.	clinical
10.1016/j.ihj.2018.11.009	Tomioka et al.	COPD was diagnosed when the ratio of forced expiratory volume in 1 s (FEV1.0) to forced vital capacity (FV), FEV1.0/FV, was less than 70% by spirometry, in accordance with the Global Initiative for Chronic Obstructive Lung Disease.	spirometry
10.1111/crj.12968	Vaes et al	ICD10	code
10.1016/j.amjcard.2010.03.026	Wakabayashi et al	In this registry, COPD was defined as the reported need for pharmacologic therapy for COPD and/or a 1-second forced expiratory volume 70% of the predicted value.	spirometry
10.1007/s00392-021-01833-y	Wang et al	COPD at baseline was defined as long-term use of bronchodilators or steroids for lung disease according to the definition in EuroSCORE	clinical
10.1002/clc.23475	Xiao et al	ICD9	code
10.1016/j.arth.2018.03.011	Yakubek et al	In the ACS NSQIP database, a patient is listed as having COPD if the medical record documents this diagnosis and one of the following within 30 days of surgery: a functional disability due to COPD (ie, dyspnea), chronic bronchodilator therapy, an exacerbation requiring hospitalization, or a forced expiratory volume in 1 second less than 75% of expected.	clinical
10.2147/CLEP.S270599	Yang et al	Due to the lack of post-bronchodilator spirometry test for most subjects, COPD was defined as pre-bronchodilator FEV1/FVC < 0.7, which was different	spirometry

		from the Global initiative for the management of chronic Obstructive Lung Disease definition.	
10.11909/j.issn.1671-5411.2022.06.005	Yao et al	COPD diagnosis was based on the standards listed in the guidelines of the American Thoracic Society/European Respiratory Society.	spirometry
10.2147/COPD.S80673	Yoshizawa et al	COPD was diagnosed based on spirometry when the FEV1 /FVC was less than 70% after inhalation of a bronchodilator, and the severity of airflow obstruction was judged according to the Global Initiative for Chronic Obstructive Lung Disease (GOLD) criteria	spirometry
10.18087/cardio.2021.11.n1820	Zafiraki et al	Patients were grouped for the follow up depending on the presence of COPD as shown by spirometry.	spirometry
10.2147/JAA.S389985	Zhang et al	The diagnosis of COPD complied with the diagnostic criteria of the Guidelines for the Diagnosis and Treatment of COPD (2013 Revised Edition)	spirometry
10.3390/cancers13153654	Zhang et al	ICD10	code
10.3390/cancers13164221	Zhang et al	na	na
10.1177/0003319712458145	Zhang et al	Patient medical records were searched for the diagnosis of COPD. To define categories of COPD severity, we used pulmonary function tests (PFTs) performed at our institution and standard cutoff values from accepted guidelines.	spirometry
10.2147/COPD.S559900	Wu et al	diagnosis of COPD (ICD-10-CM: J40-J44)	code

Abbreviations: AFO – airflow obstruction; AMI – acute myocardial infarction; ATC – anatomical therapeutic chemical code; COPD – chronic obstructive pulmonary disease; FEV1 – forced expiratory volume in 1 second; FVC – forced vital capacity; GOLD – global initiative for obstructive lung disease; ICD – international classification of diseases; L- litre; LABA – long acting beta adrenoceptor agonist; LAMA – long acting muscarinic receptor antagonist; LLN – lower limit of normal; mmHg – millimetres of mercury; PFTs- pulmonary function tests; STS – society of thoracic surgeons;

Supplementary Table 21: Univariate meta-regression for significant associations between covariate and outcome

outcome	moderator	k_outcome	k_moderator	term	ratio_of_RR_95CI	p_value	I^2
all_mortality_alltime	mean_age_overall_y	121	98	mean_age_overall_y	0.98 (0.97-0.99)	0.00045	99.1
all_mortality_within_1yr	copd_definition_group	40	37	copd_definition_groupcode	0.61 (0.45-0.81)	0.00089	99.5
all_mortality_over_1yr	mean_age_overall_y	30	23	mean_age_overall_y	0.96 (0.93-0.98)	0.0013	99.1
hf_hospitalisation	copd_definition_group	9	9	copd_definition_groupspirometry	2.36 (1.38-4.02)	0.0016	98.1
all_mortality_alltime	risk_group	121	121	risk_grouplow	1.26 (1.05-1.50)	0.012	99.8
all_mortality_alltime	dm_perc	121	100	dm_perc	0.99 (0.99-1.00)	0.029	99.0
atrial_fibrillation	mean_age_overall_y	16	14	mean_age_overall_y	0.97 (0.95-1.00)	0.030	85.8
all_mortality_within_1yr	mean_age_overall_y	40	31	mean_age_overall_y	0.97 (0.95-1.00)	0.030	99.5
all_mortality_over_1yr	risk_group	30	30	risk_grouplow	1.58 (1.03-2.41)	0.036	99.8
heart_failure	risk_group	22	22	risk_grouplow	1.79 (1.00-3.18)	0.049	99.9
all_mortality_over_1yr	copd_definition_group	30	26	copd_definition_groupspirometry	1.97 (1.00-3.87)	0.049	99.8

Abbreviations: 95CI – 95% confidence interval; COPD – chronic obstructive pulmonary disease; dm – diabetes mellitus; I2 I squared; hf – heart failure; k – number of; perc – percentage; RR – risk ratio; y – year;

Supplementary Table 22: Multivariate meta-regression for significant associations between covariate and outcome

outcome	status	k_outcome	k_complete_case	term	ratio_of_RR_95CI	p_value	I^2
all_mortality_alltime	Model fitted. Usable moderators: mean age, male (%), smoker, htn (%), dm (%), risk_group, COPD definition group	121	47	htn_perc	1.01 (1.00-1.01)	0.012	88.9
mi	Model fitted. Usable moderators: mean age, male (%), smoker, htn (%), dm (%), risk_group, COPD definition group	33	17	dm_perc	1.03 (1.00-1.07)	0.033	74.1
all_mortality_1mo	Model fitted. Usable moderators: mean age, male (%), smoker, htn (%), dm (%), risk_group, COPD definition group	37	13	male_perc	1.02 (1.00-1.04)	0.035	31.6
all_mortality_within_1yr	Model fitted. Usable moderators: mean age, male (%), smoker, htn (%), dm (%), risk_group, COPD definition group	40	13	male_perc	1.02 (1.00-1.04)	0.035	31.6
all_mortality_alltime	Model fitted. Usable moderators: mean age, male (%), smoker, htn (%), dm (%), risk_group, COPD definition group	121	47	male_perc	1.01 (1.00-1.01)	0.047	88.9

Abbreviations: 95CI – 95% confidence interval; COPD – chronic obstructive pulmonary disease; dm – diabetes mellitus; I2 I squared; hf – heart failure; htn – hypertension; k – number of; mi – myocardial infarction; perc – percentage; RR – risk ratio; y – year;

Supplementary Figure 1: Risk of bias assessment for each domain in each study

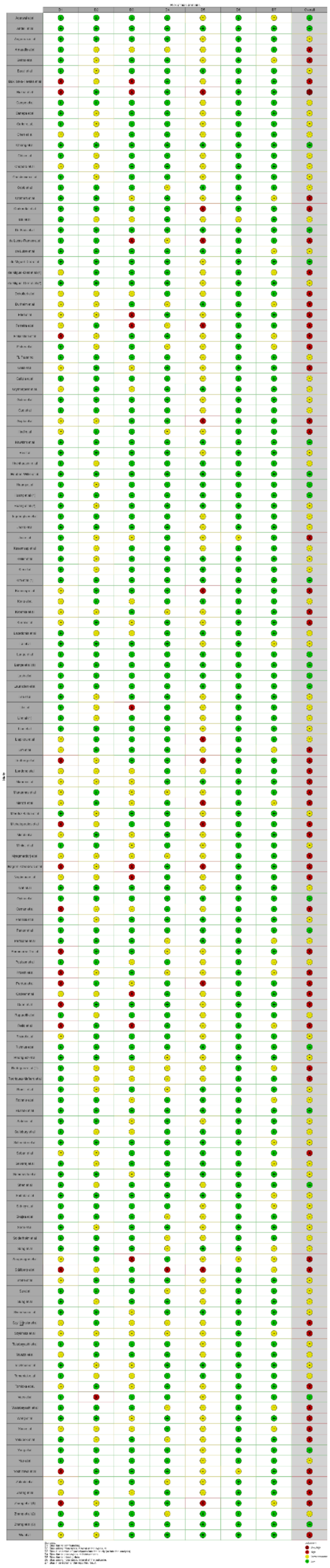

Supplementary Figure 2: Traffic light plot for risk of bias assessment

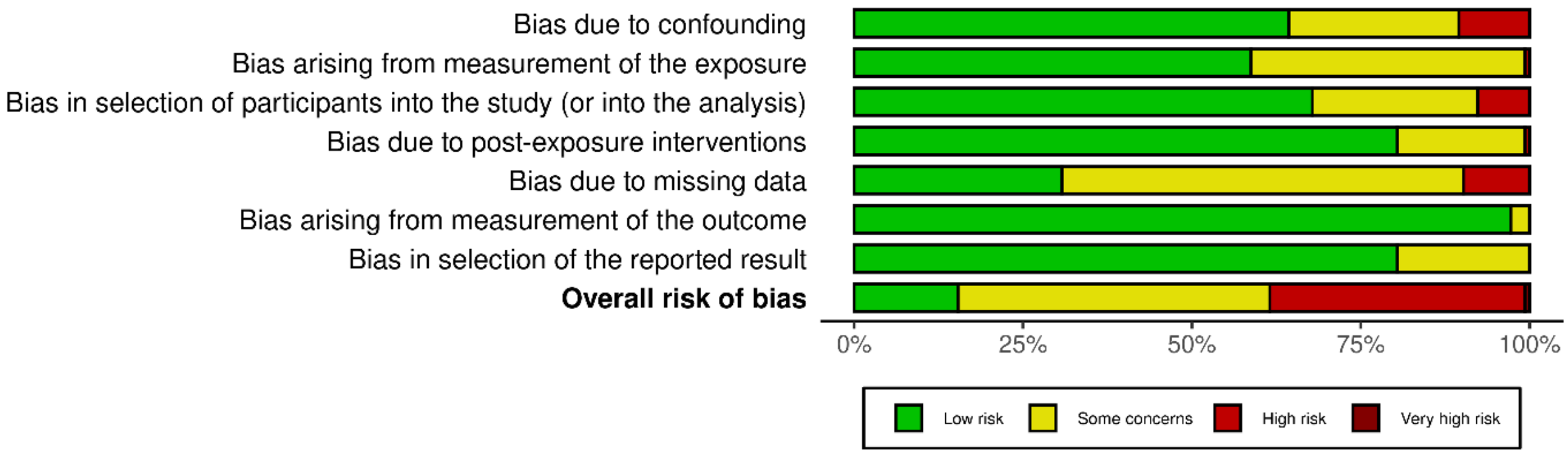

Supplementary Figure 3: Funnel plot for myocardial infarction

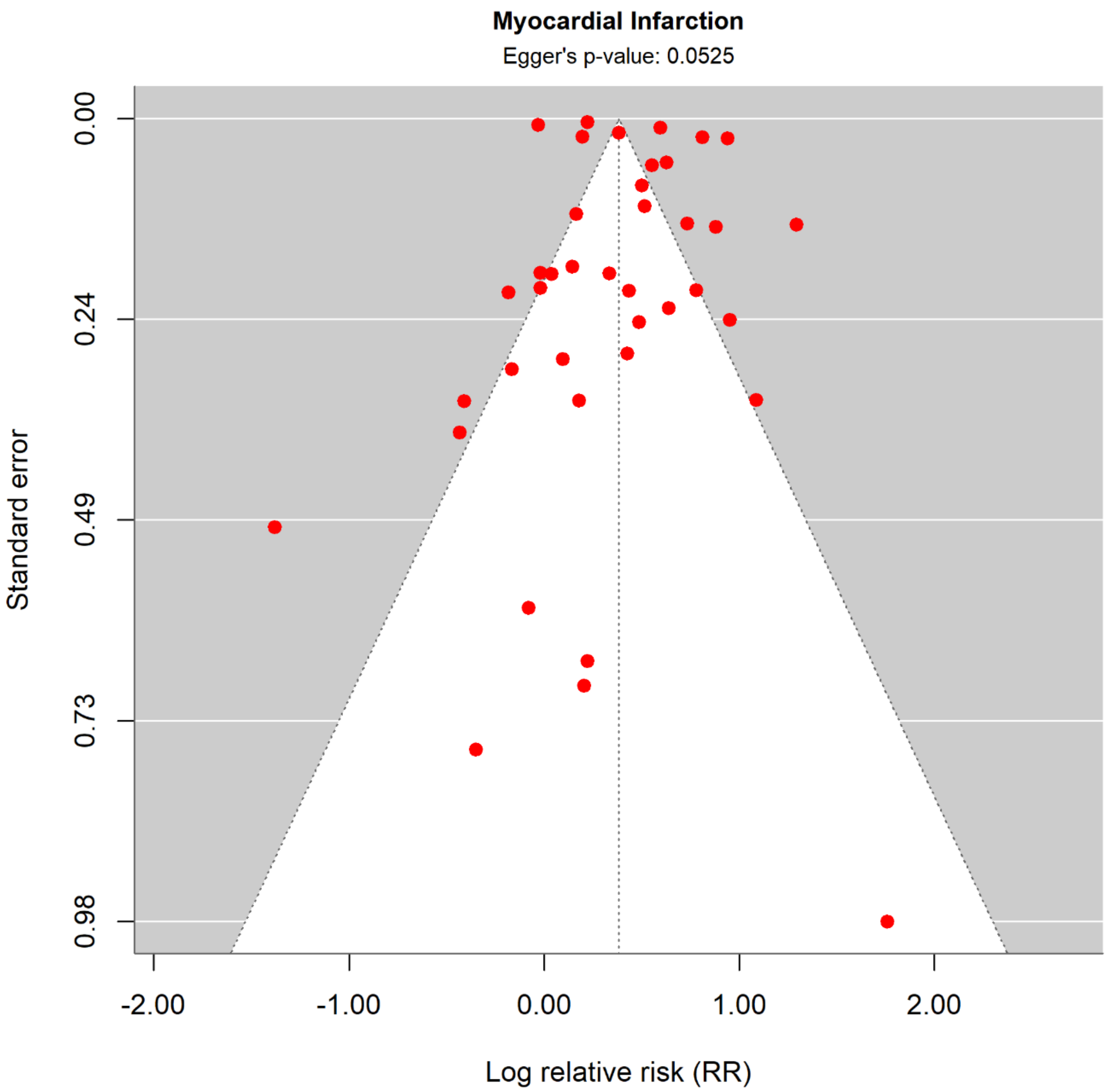

Supplementary Figure 4: Funnel plot for Heart Failure

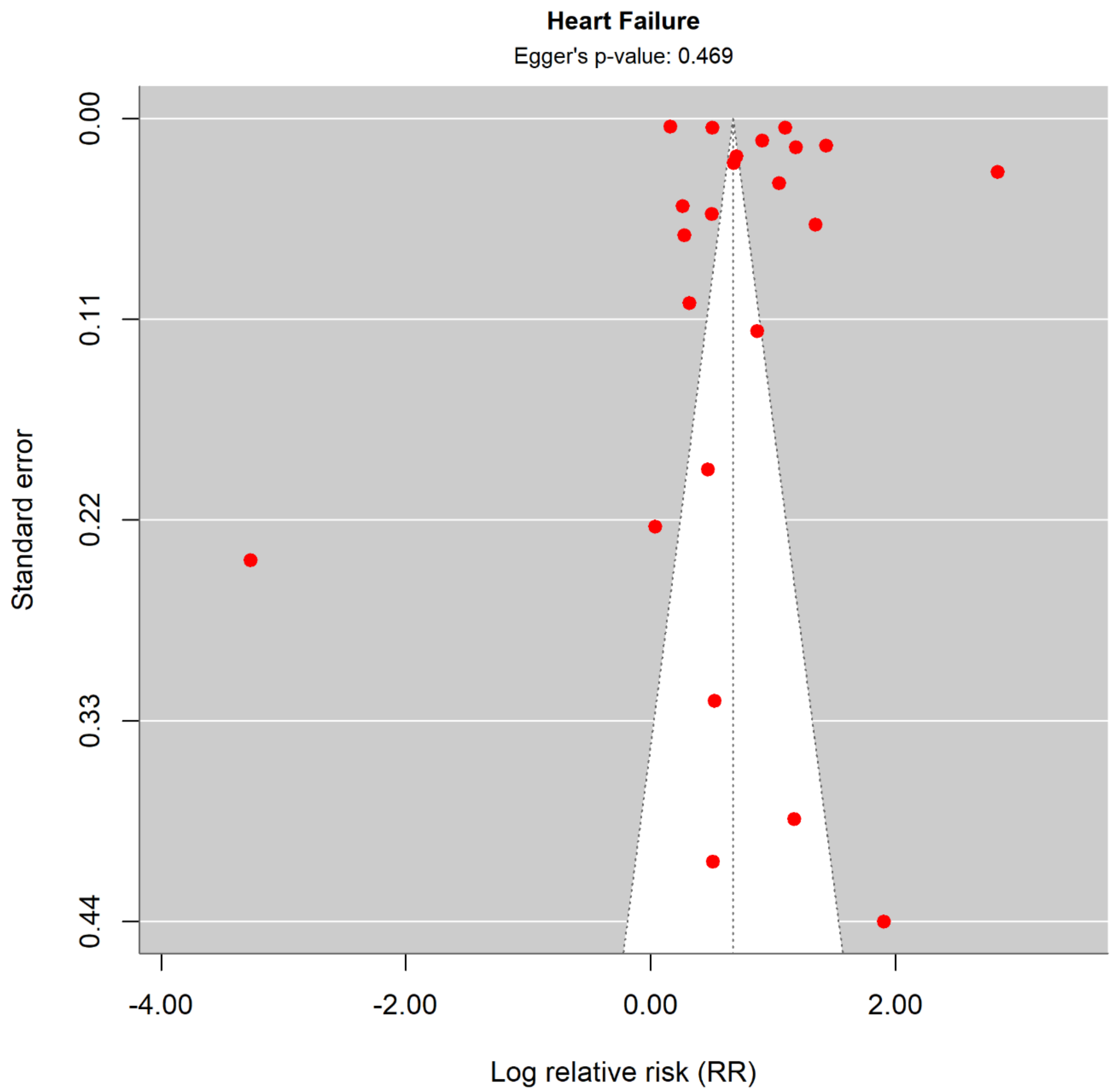

Supplementary Figure 5: Funnel plot for Heart Failure Hospitalisation

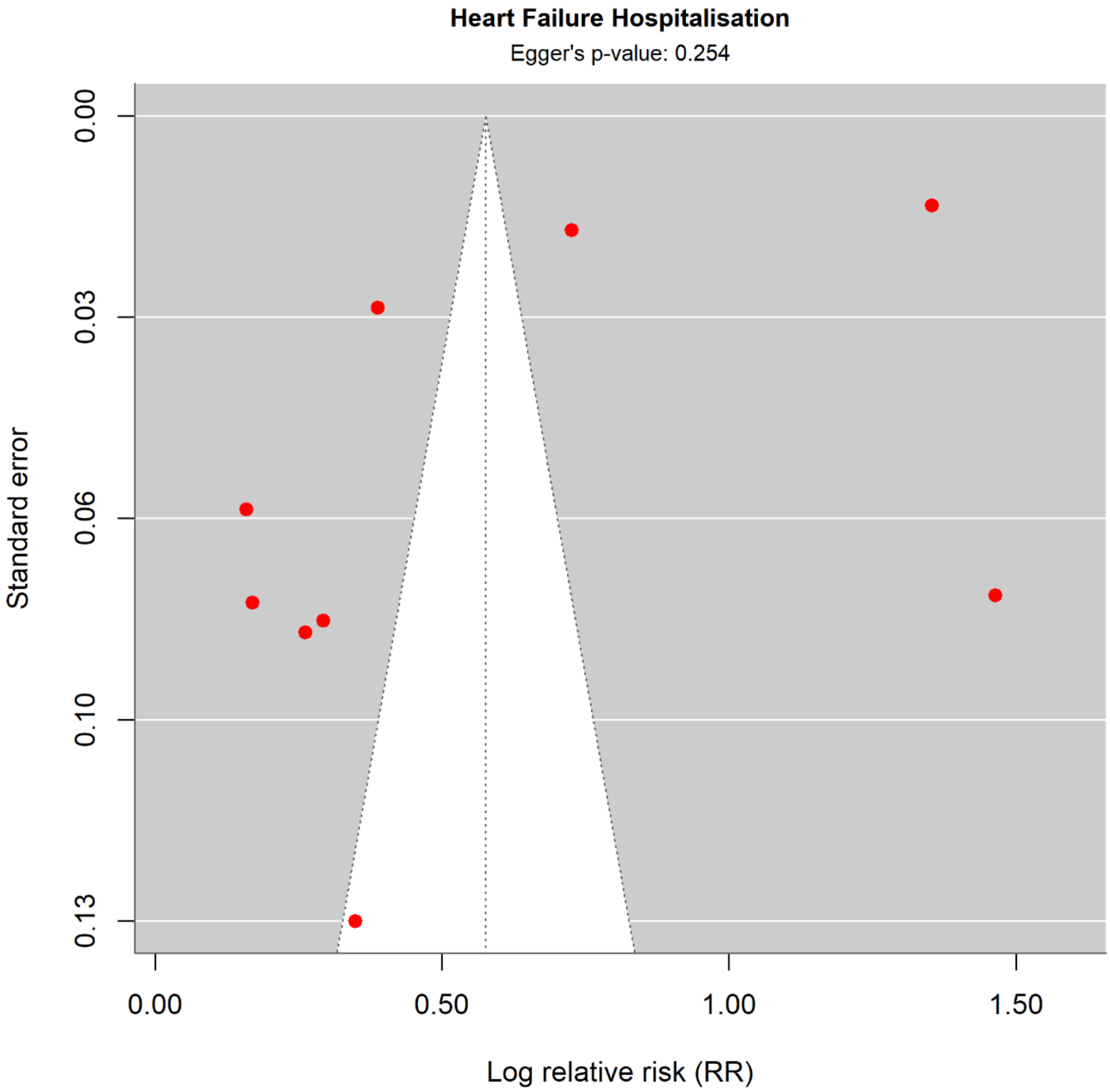

Supplementary Figure 6: Funnel plot for Atrial Fibrillation

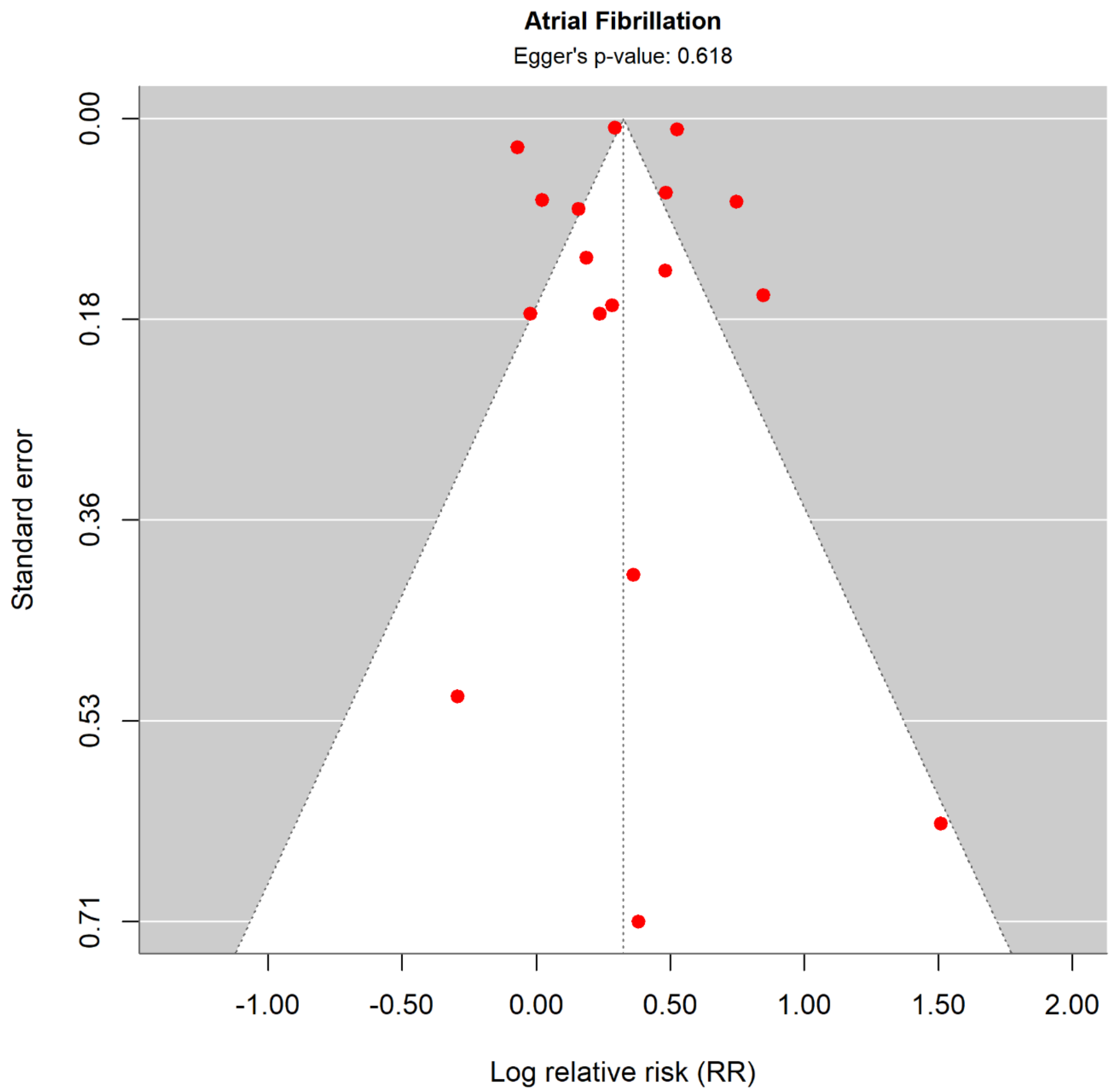

Supplementary Figure 7: Funnel plot for Stroke

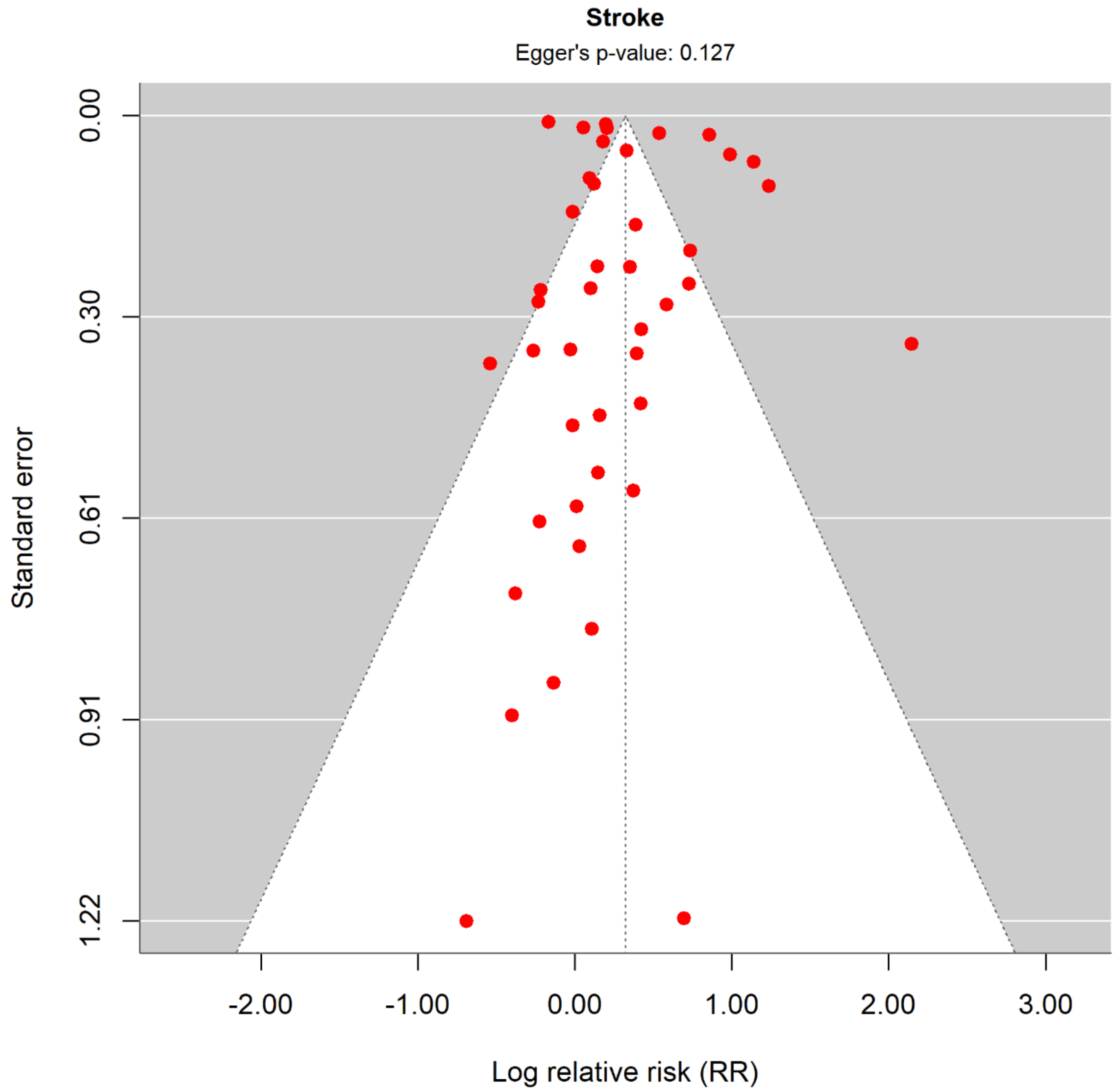

Supplementary Figure 8: Funnel plot for Peripheral Arterial Disease

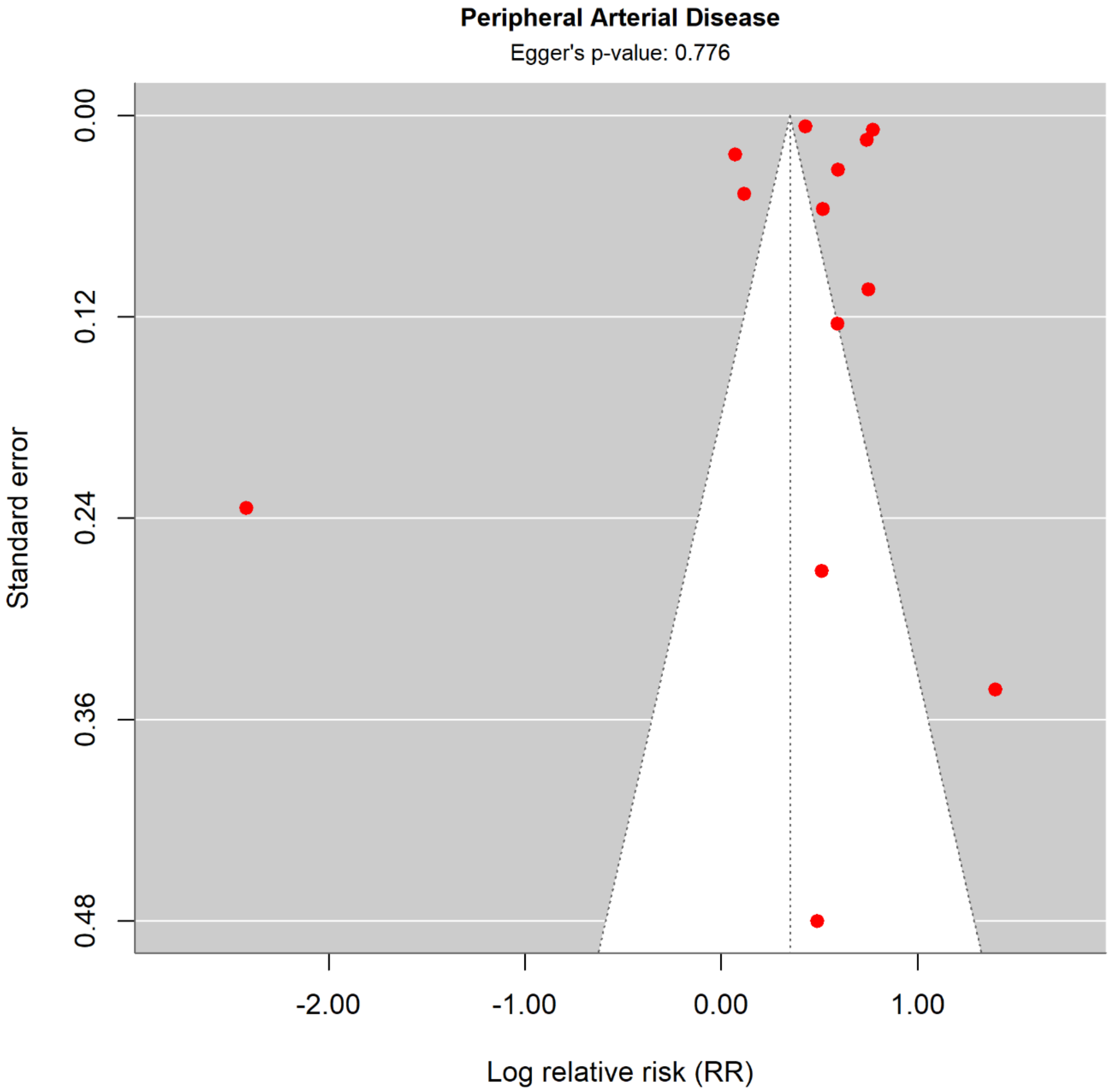

Supplementary Figure 9: Funnel plot for Chronic Kidney Disease

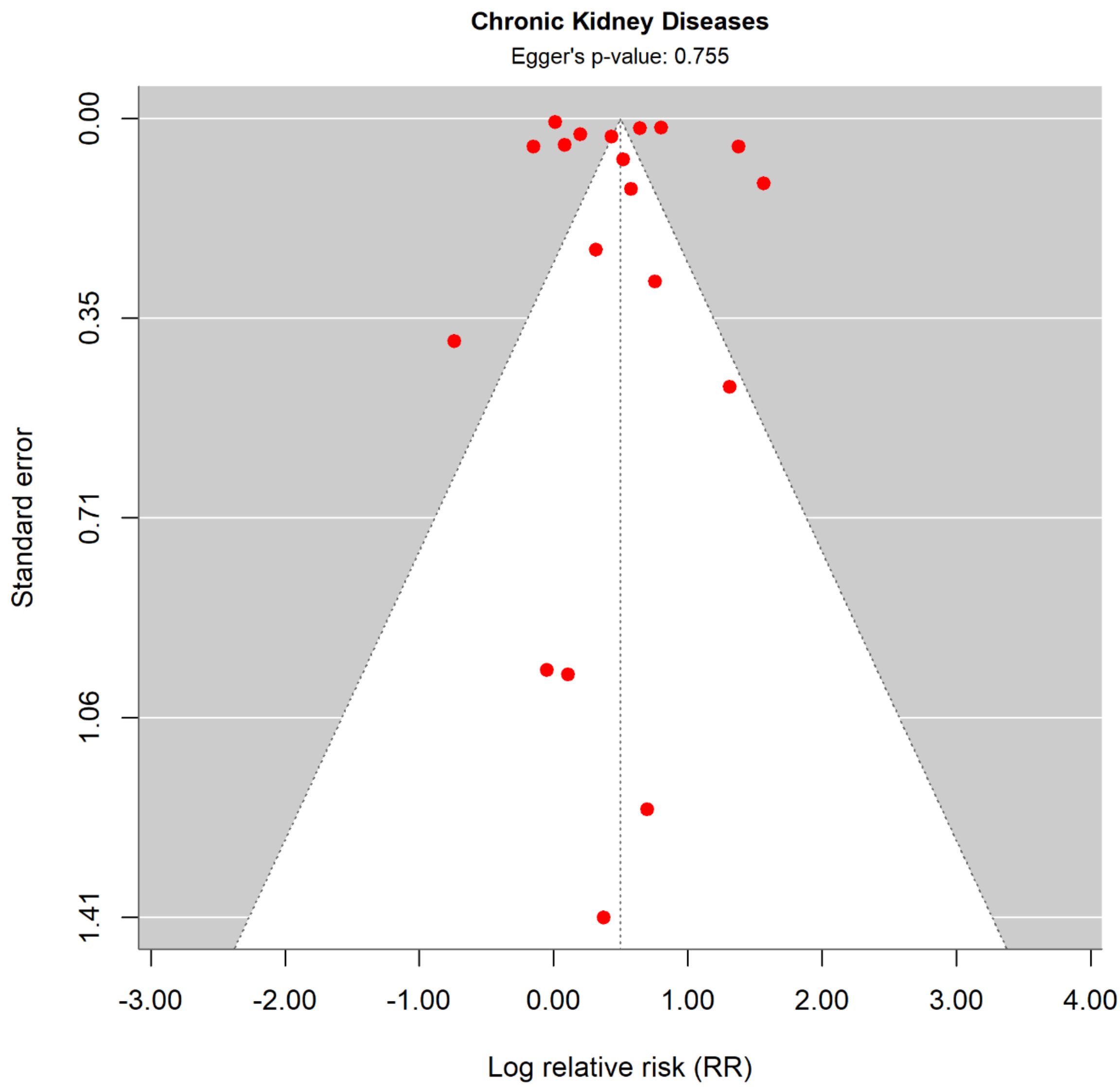

Supplementary Figure 10: Funnel plot for Cardiovascular Mortality

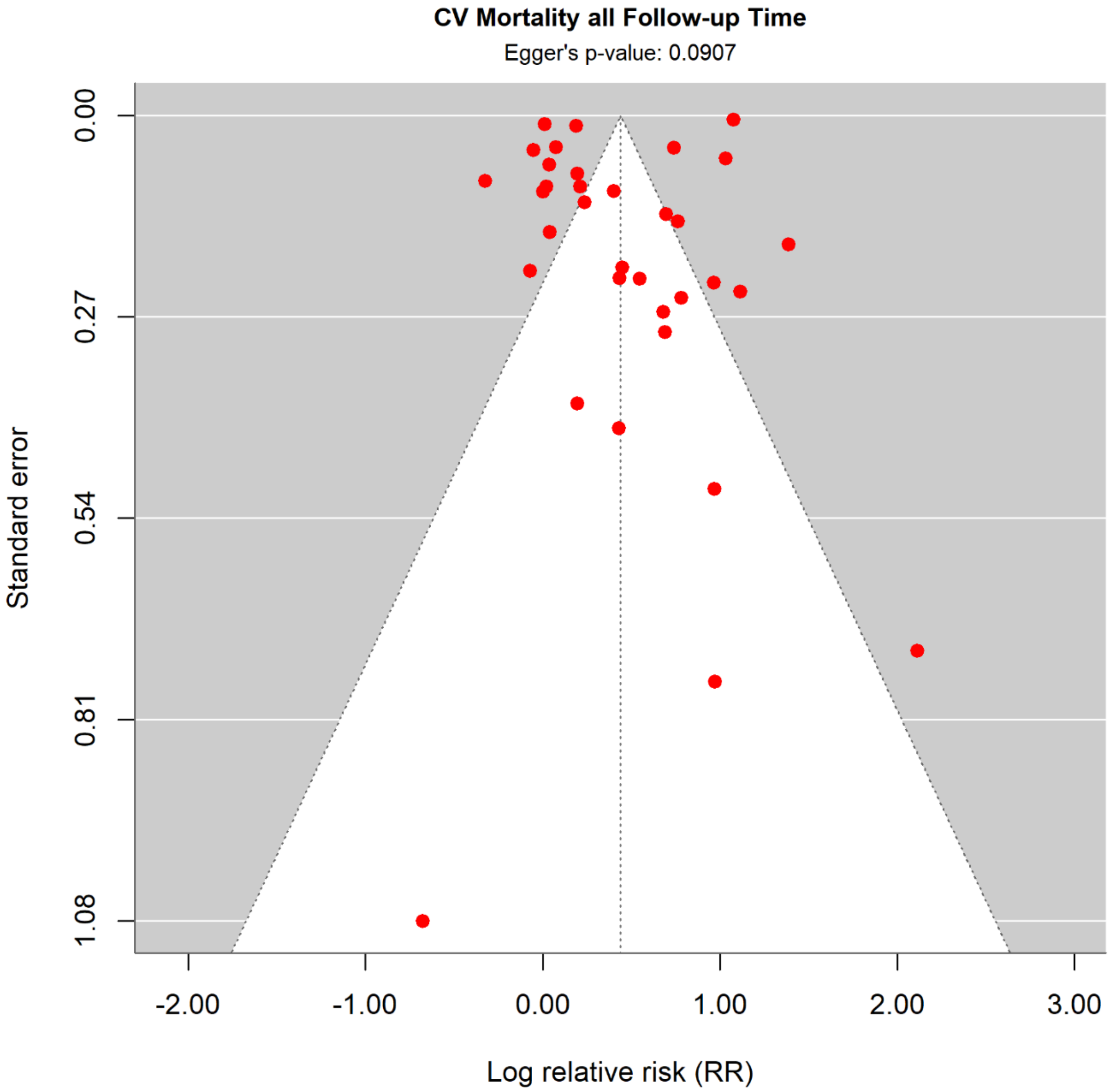

Supplementary Figure 11: Funnel plot for All-cause Mortality

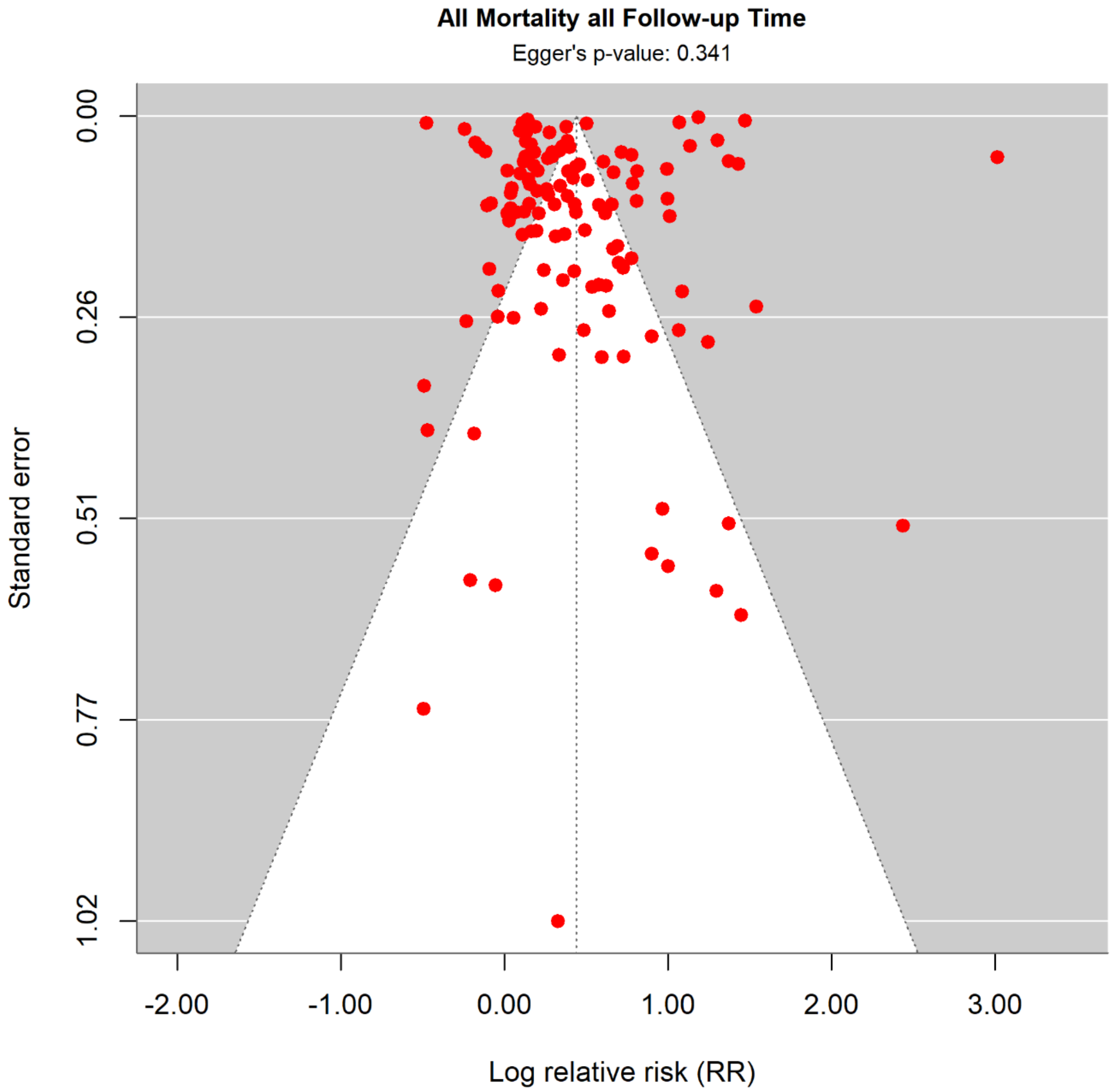

Supplementary Figure 12: Funnel plot for All-cause Mortality <1 year

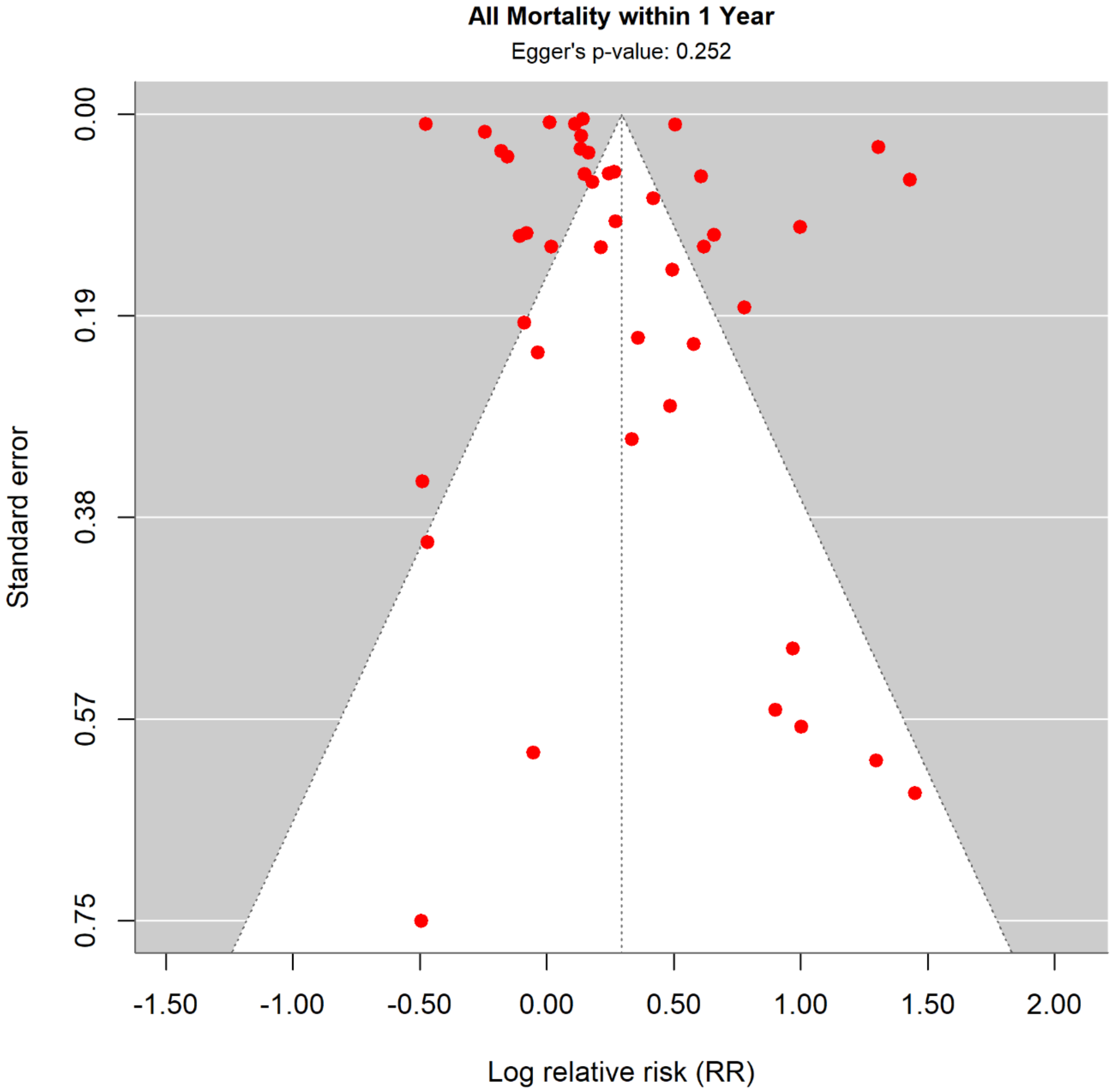

Supplementary Figure 13: Funnel plot for All-cause Mortality over 1 year

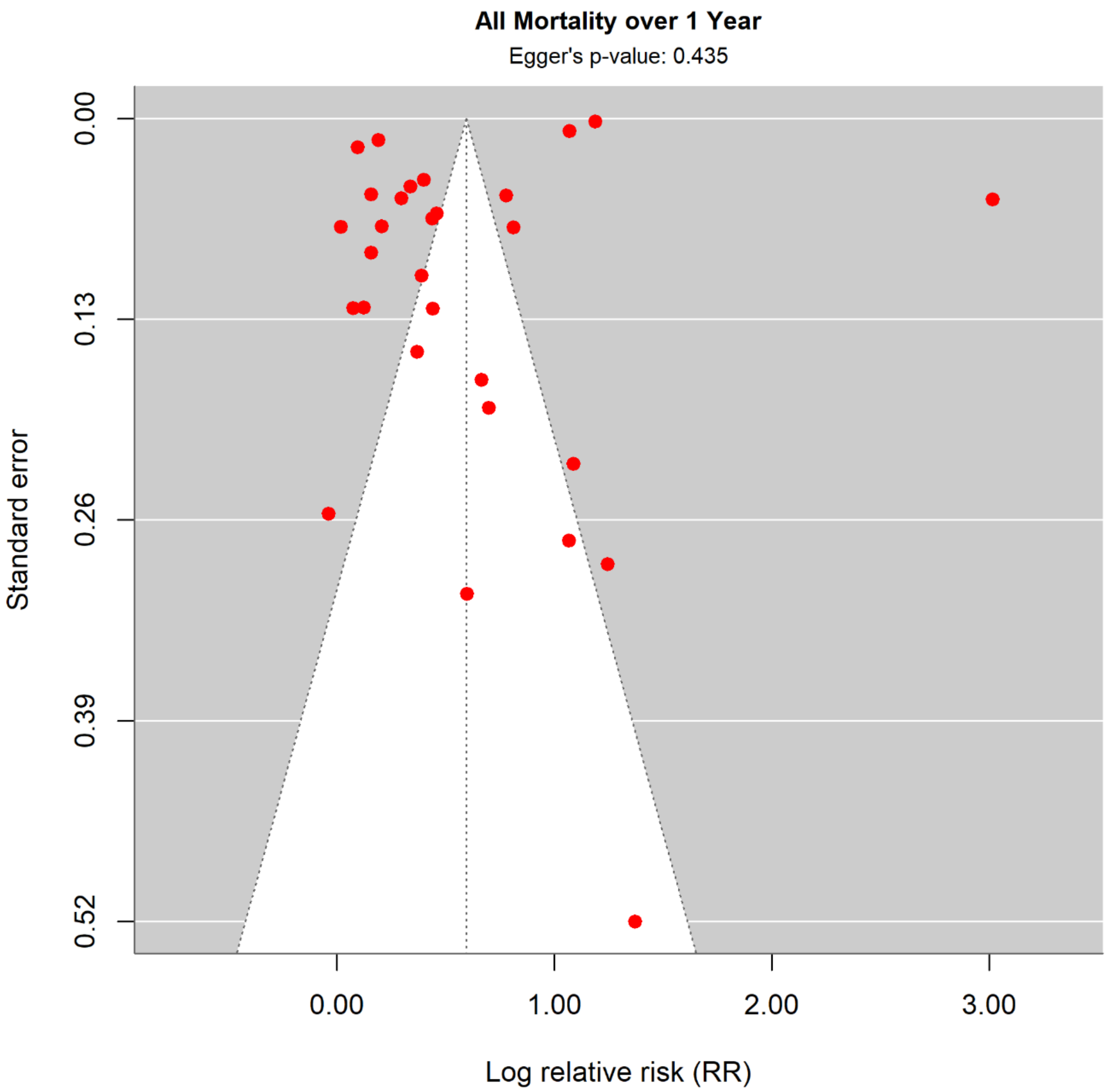

Supplementary Figure 14: Leave one out analyses for myocardial infarction

Myocardial Infarction

Study removed	Risk Ratio [95% CI]	P
FULL MODEL	1.57 [1.38, 1.79]	<0.001
REMOVE: ANGOURAS ET AL 2010	1.58 [1.38, 1.80]	<0.001
REMOVE: COBB ET AL 2024	1.54 [1.36, 1.75]	<0.001
REMOVE: CROTHERS ET AL 2023	1.56 [1.37, 1.78]	<0.001
REMOVE: CURKENDAL ET AL 2006	1.57 [1.37, 1.79]	<0.001
REMOVE: DOKOLLARI ET AL 2024	1.58 [1.39, 1.80]	<0.001
REMOVE: DURHEIM ET AL 2018	1.58 [1.38, 1.80]	<0.001
REMOVE: FINKELSTEIN ET AL 2009	1.56 [1.36, 1.78]	<0.001
REMOVE: GUPTA ET AL 2022	1.57 [1.37, 1.78]	<0.001
REMOVE: HUANG ET AL 2019	1.55 [1.36, 1.77]	<0.001
REMOVE: HUANG ET AL 2019	1.55 [1.36, 1.77]	<0.001
REMOVE: KASEMSAP ET AL 2022	1.59 [1.39, 1.82]	<0.001
REMOVE: KELLER ET AL 2018	1.61 [1.41, 1.83]	<0.001
REMOVE: KURMI ET AL 2018	1.59 [1.39, 1.82]	<0.001
REMOVE: LASH ET AL 2011	1.56 [1.37, 1.79]	<0.001
REMOVE: LI ET AL 2024	1.60 [1.40, 1.82]	<0.001
REMOVE: NAGORNI-OBRADOVIC ET AL 2014	1.59 [1.40, 1.82]	<0.001
REMOVE: OSMAN ET AL 2020	1.57 [1.37, 1.79]	<0.001
REMOVE: PERTICONE ET AL 2021	1.57 [1.37, 1.79]	<0.001
REMOVE: RAPARELLI ET AL 2018	1.57 [1.38, 1.80]	<0.001
REMOVE: RODRIGUEZ ET AL 2010	1.60 [1.40, 1.82]	<0.001
REMOVE: SCHNEIDER ET AL 2010	1.55 [1.37, 1.77]	<0.001
REMOVE: SIDNEY ET AL 2005	1.56 [1.37, 1.79]	<0.001
REMOVE: SKAJAA ET AL 2023	1.58 [1.38, 1.80]	<0.001
REMOVE: SODE ET AL. 2011	1.59 [1.39, 1.81]	<0.001
REMOVE: STÄLLBERG ET AL 2018	1.55 [1.36, 1.77]	<0.001
REMOVE: SUNG ET AL 2012	1.53 [1.36, 1.72]	<0.001
REMOVE: TOMANIAK ET AL 2020	1.59 [1.39, 1.81]	<0.001
REMOVE: WANG ET AL 2021	1.57 [1.38, 1.80]	<0.001
REMOVE: YAKUBEK ET AL 2018	1.59 [1.39, 1.81]	<0.001
REMOVE: YAO ET AL 2022	1.60 [1.40, 1.82]	<0.001
REMOVE: ZAFIRAKI ET AL 2021	1.57 [1.38, 1.80]	<0.001
REMOVE: ZHANG ET AL 2013	1.57 [1.37, 1.80]	<0.001
REMOVE: SALEH ET AL 2012	1.60 [1.41, 1.82]	<0.001

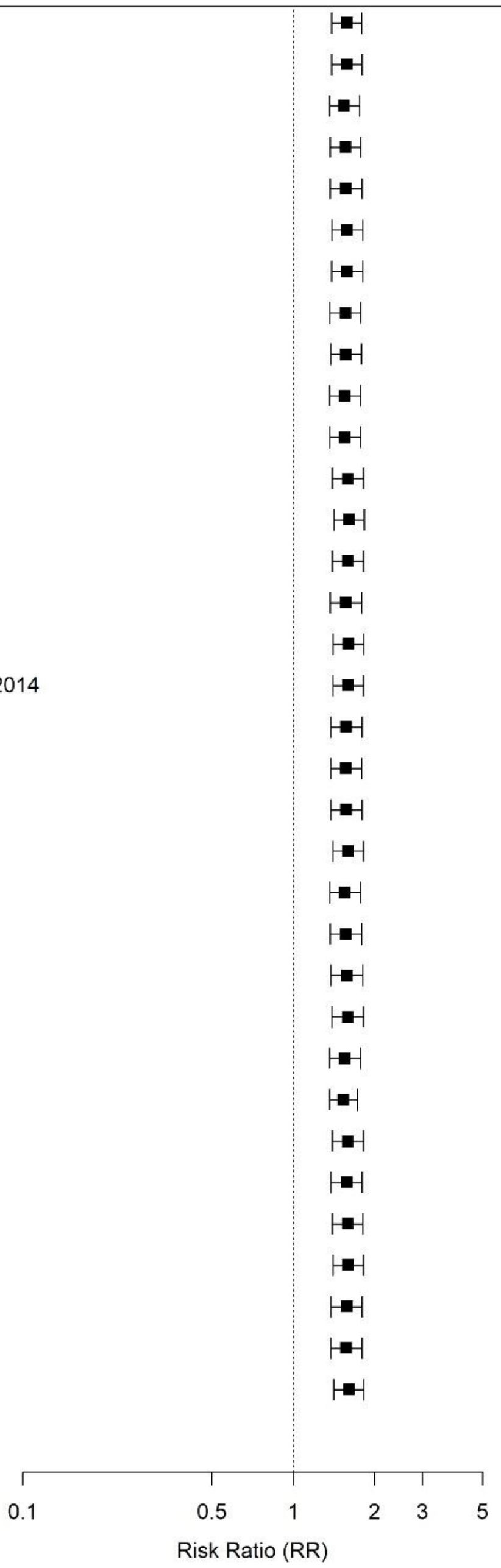

CV – cardiovascular; CI – confidence interval; I2 denotes the heterogeneity statistic.; RR – risk ratio

Supplementary Figure 15: Leave one out analyses for heart failure

Heart Failure

Study removed	Risk Ratio [95% CI]	P
FULL MODEL	2.33 [1.78, 3.06]	<0.001
REMOVE: AGARWAL ET AL 2017	2.37 [1.79, 3.15]	<0.001
REMOVE: BEHAR ET AL 1992	2.40 [1.81, 3.17]	<0.001
REMOVE: BUCCI ET AL 2024	2.30 [1.74, 3.05]	<0.001
REMOVE: CARTER ET AL. 2019	2.35 [1.77, 3.12]	<0.001
REMOVE: COBB ET AL 2024	2.26 [1.71, 2.99]	<0.001
REMOVE: CURKENDAL ET AL 2006	2.31 [1.74, 3.07]	<0.001
REMOVE: DE LUCAS-RAMOS ET AL 2012	2.09 [1.73, 2.53]	<0.001
REMOVE: DE MIGUEL-DIEZ ET AL 2017	2.41 [1.83, 3.18]	<0.001
REMOVE: DURHEIM ET AL 2018	2.42 [1.84, 3.18]	<0.001
REMOVE: HADI ET AL 2010	2.37 [1.79, 3.15]	<0.001
REMOVE: KROENKE ET AL 1993	2.36 [1.78, 3.12]	<0.001
REMOVE: KURMI ET AL 2018	2.35 [1.77, 3.13]	<0.001
REMOVE: LASH ET AL 2011	2.30 [1.73, 3.06]	<0.001
REMOVE: LAURISDEN ET AL 2023	2.40 [1.82, 3.17]	<0.001
REMOVE: MINIATI ET AL 2014	2.37 [1.79, 3.14]	<0.001
REMOVE: RODRIGUEZ ET AL 2010	2.33 [1.75, 3.10]	<0.001
REMOVE: SIDNEY ET AL 2005	2.27 [1.72, 3.01]	<0.001
REMOVE: SKAJAA ET AL 2023	2.32 [1.75, 3.09]	<0.001
REMOVE: STÄLLBERG ET AL 2018	2.29 [1.73, 3.04]	<0.001
REMOVE: SZYLIŃSKA ET AL 2022	2.36 [1.78, 3.13]	<0.001
REMOVE: TOMIOKA ET AL. 2019	2.25 [1.71, 2.95]	<0.001
REMOVE: YANG ET AL 2020	2.39 [1.81, 3.17]	<0.001

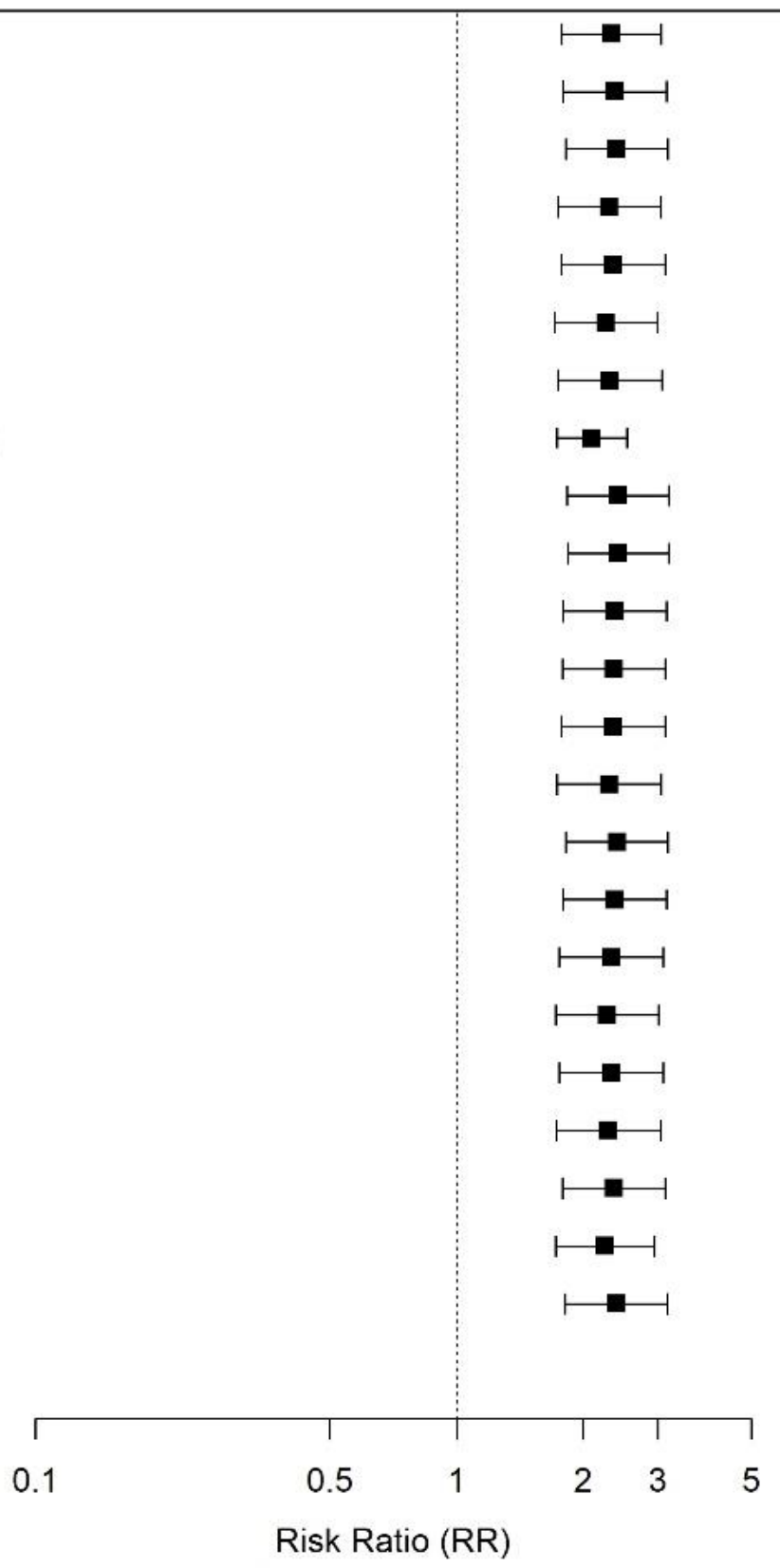

CV – cardiovascular; CI – confidence interval; I2 denotes the heterogeneity statistic.; RR – risk ratio

Supplementary Figure 16: Leave one out analyses for heart failure hospitalisation

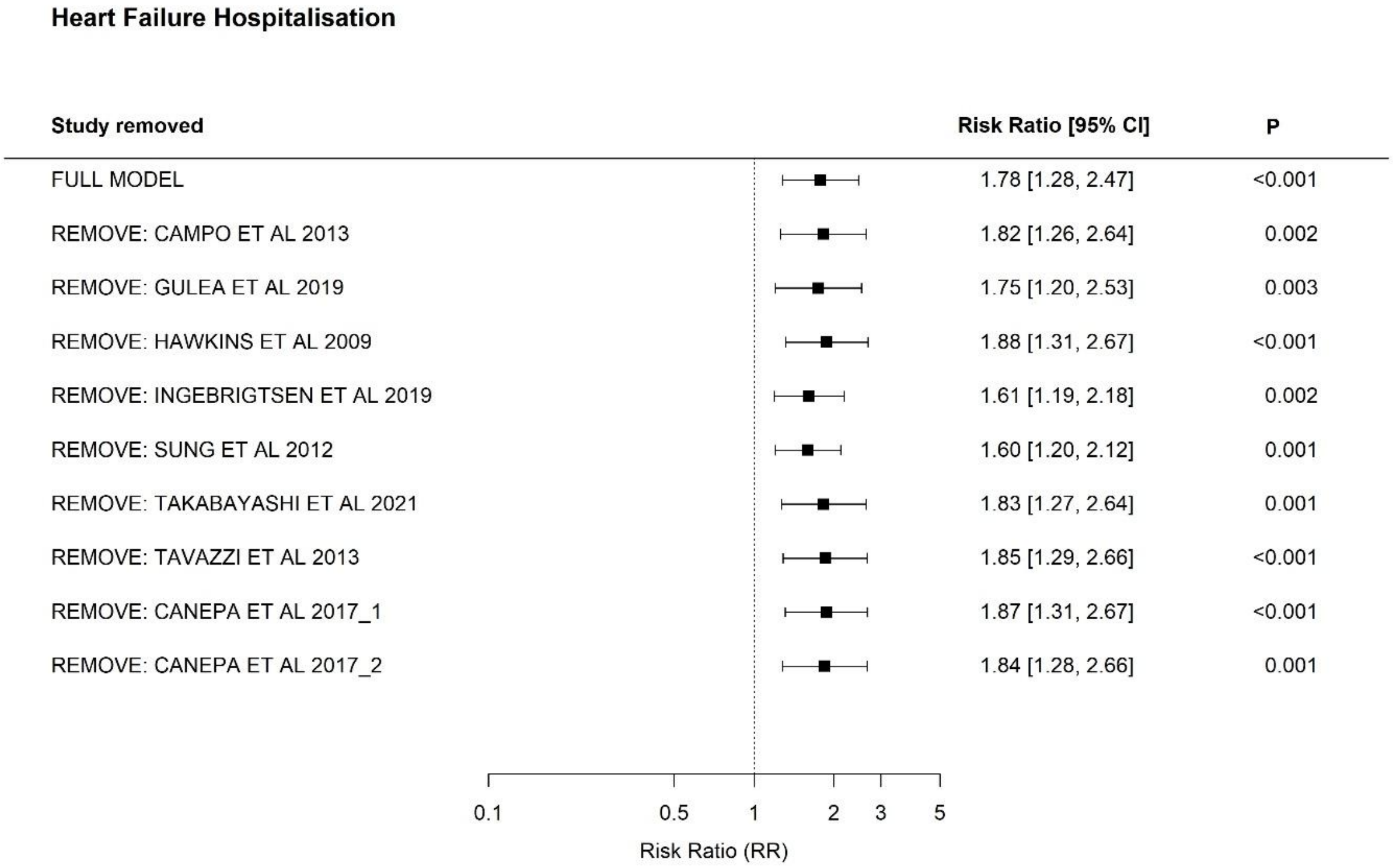

CV – cardiovascular; CI – confidence interval; I2 denotes the heterogeneity statistic.; RR – risk ratio

Supplementary Figure 17: Leave one out analyses for atrial fibrillation

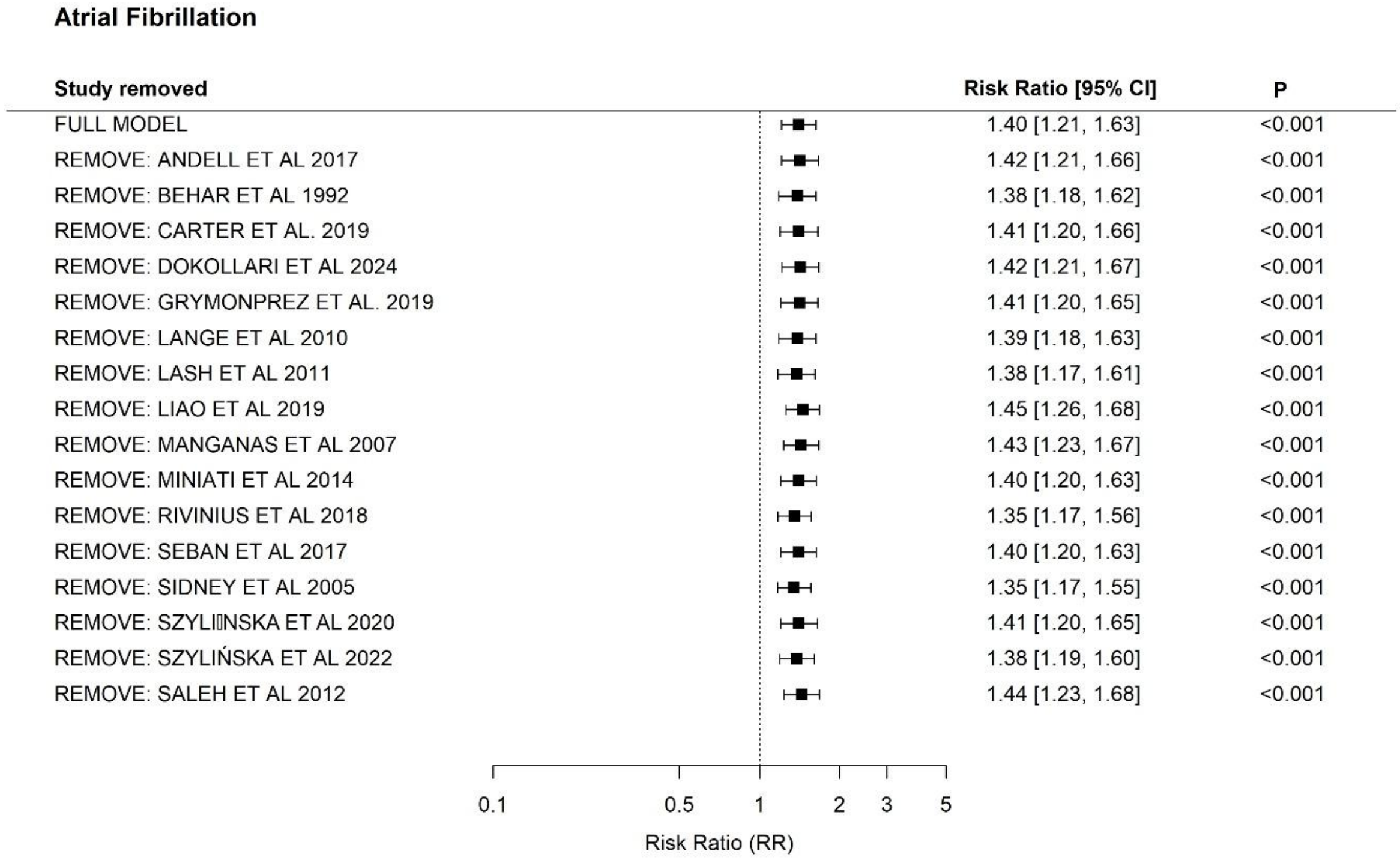

CV – cardiovascular; CI – confidence interval; I2 denotes the heterogeneity statistic.; RR – risk ratio

Supplementary Figure 18: Leave one out analyses for stroke

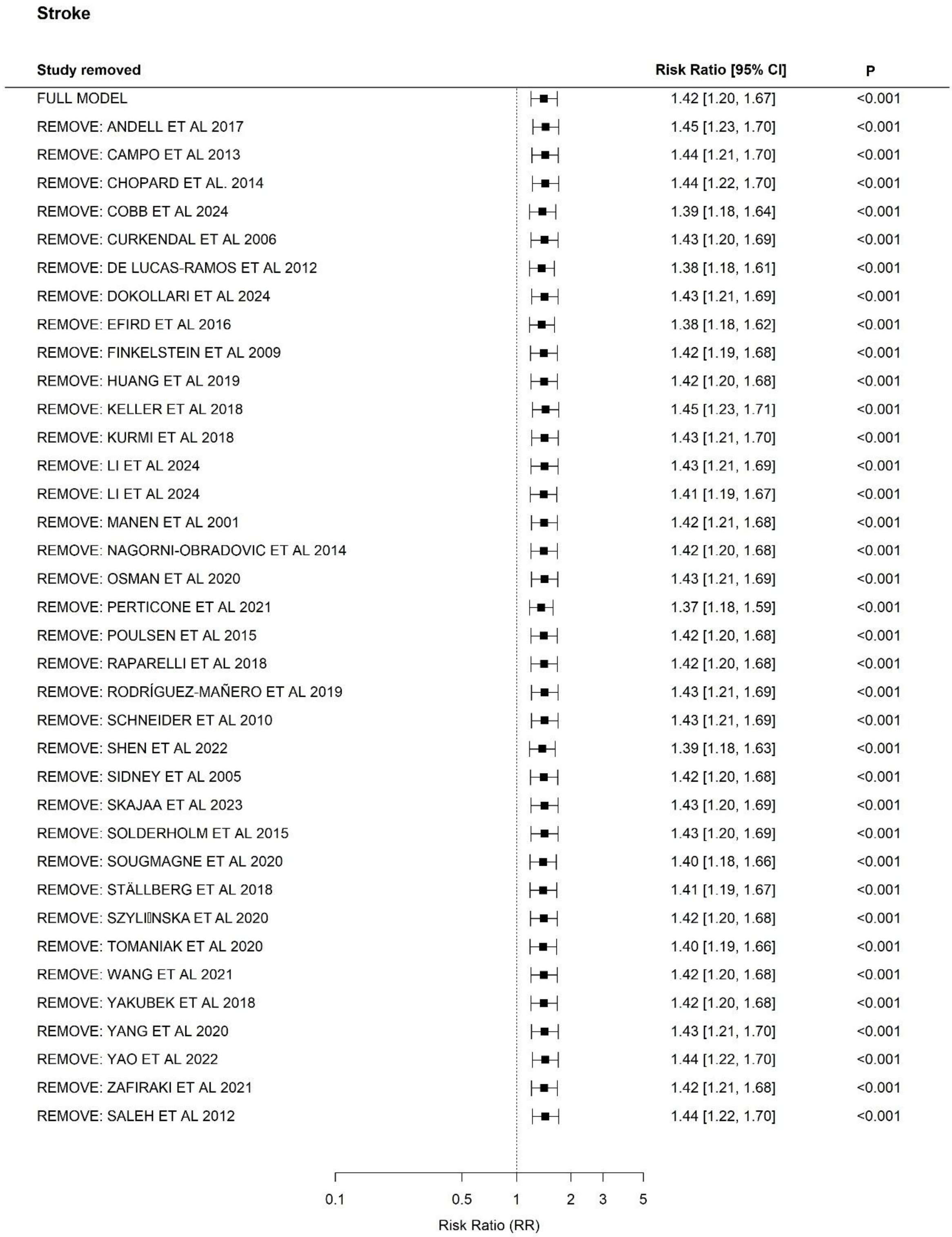

CV – cardiovascular; CI – confidence interval; I2 denotes the heterogeneity statistic.; RR – risk ratio

Supplementary Figure 19: Leave one out analyses for peripheral arterial diseases

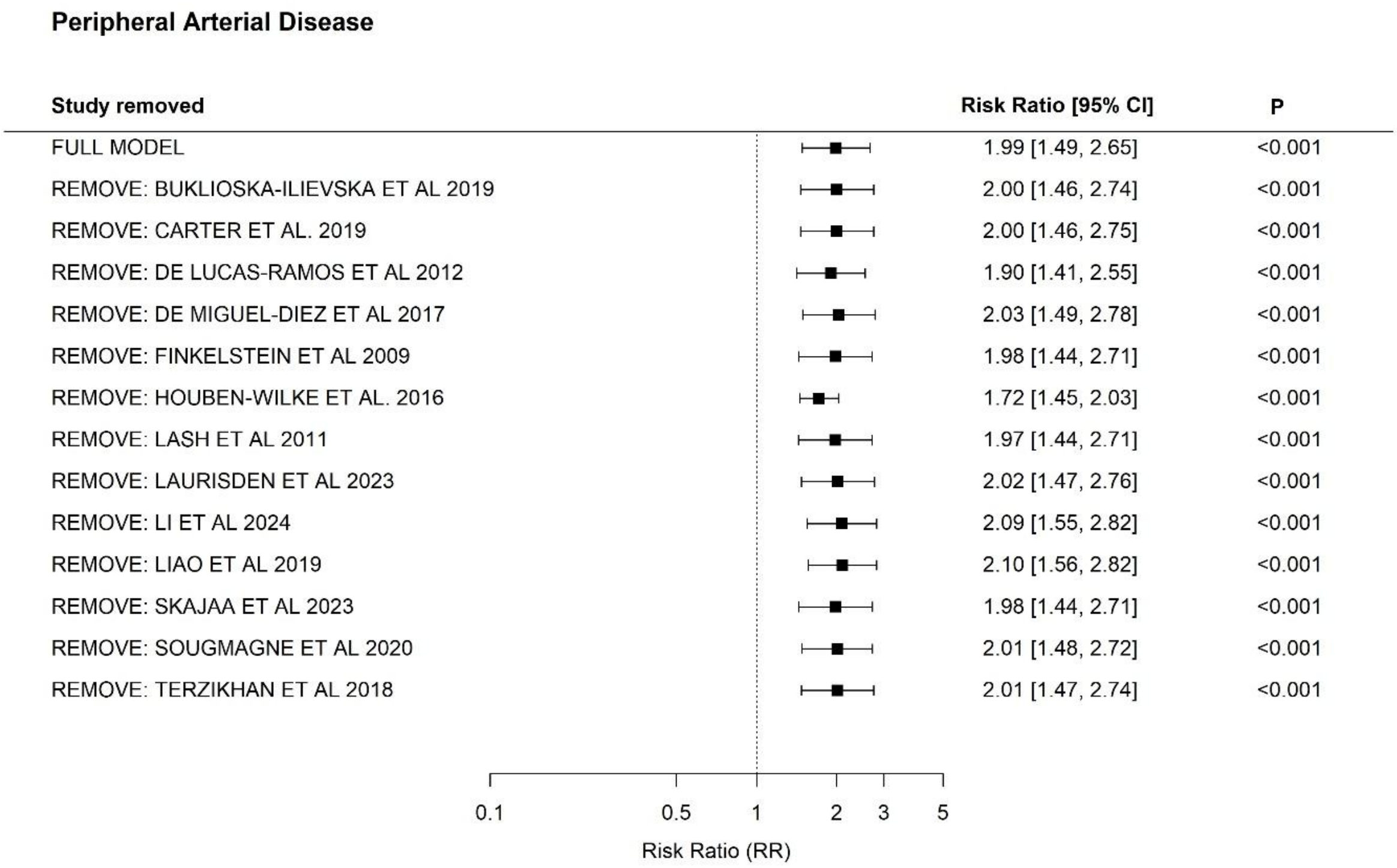

CV – cardiovascular; CI – confidence interval; I2 denotes the heterogeneity statistic.; RR – risk ratio

Supplementary Figure 20: Leave one out analyses for cardiovascular mortality

CV Mortality all Follow-up Time

Study removed	Risk Ratio [95% CI]	P
FULL MODEL	1.56 [1.33, 1.83]	<0.001
REMOVE: REZAEI ET AL 2018	1.58 [1.35, 1.86]	<0.001
REMOVE: CHIANG ET AL 2022	1.59 [1.35, 1.86]	<0.001
REMOVE: DURHEIM ET AL 2018	1.57 [1.33, 1.84]	<0.001
REMOVE: HAWKINS ET AL 2009	1.59 [1.35, 1.86]	<0.001
REMOVE: KURMI ET AL 2018	1.58 [1.34, 1.86]	<0.001
REMOVE: MINATI ET AL 2014	1.56 [1.33, 1.84]	<0.001
REMOVE: MINIATI ET AL 2014	1.54 [1.31, 1.81]	<0.001
REMOVE: PARKER ET AL 2014	1.59 [1.35, 1.86]	<0.001
REMOVE: PARKER ET AL 2014	1.56 [1.33, 1.83]	<0.001
REMOVE: PARKER ET AL 2014	1.53 [1.31, 1.79]	<0.001
REMOVE: PROIETTI ET AL 2016	1.55 [1.32, 1.82]	<0.001
REMOVE: RAPARELLI ET AL 2018	1.54 [1.31, 1.80]	<0.001
REMOVE: SHIBATA ET AL 2013	1.60 [1.37, 1.87]	<0.001
REMOVE: SIDNEY ET AL 2005	1.54 [1.31, 1.82]	<0.001
REMOVE: SONG ET AL 2017	1.59 [1.35, 1.86]	<0.001
REMOVE: TAVAZZI ET AL 2013	1.59 [1.35, 1.86]	<0.001
REMOVE: TOMIOKA ET AL. 2019	1.53 [1.31, 1.79]	<0.001
REMOVE: WAKABAYASHI ET AL 2010	1.55 [1.32, 1.82]	<0.001
REMOVE: YAO ET AL 2022	1.55 [1.32, 1.82]	<0.001
REMOVE: ZAFIRAKI ET AL 2021	1.57 [1.34, 1.84]	<0.001
REMOVE: BUCCI ET AL 2024	1.55 [1.33, 1.82]	<0.001
REMOVE: HUANG ET AL 2014	1.56 [1.33, 1.84]	<0.001
REMOVE: RODRIGUEZ ET AL 2010	1.52 [1.30, 1.78]	<0.001
REMOVE: ANDELL ET AL 2017	1.59 [1.36, 1.86]	<0.001
REMOVE: NOH ET AL 2024	1.52 [1.30, 1.77]	<0.001
REMOVE: HUANG ET AL 2019	1.51 [1.30, 1.75]	<0.001
REMOVE: CHOPARD ET AL. 2014	1.58 [1.34, 1.85]	<0.001
REMOVE: CHOPARD ET AL. 2014_1	1.57 [1.34, 1.85]	<0.001
REMOVE: WANG ET AL 2021_1	1.55 [1.32, 1.81]	<0.001
REMOVE: CANEPA ET AL 2017_1	1.58 [1.34, 1.85]	<0.001
REMOVE: CANEPA ET AL 2017_2	1.58 [1.35, 1.86]	<0.001
REMOVE: HUANG ET AL 2019_1	1.56 [1.32, 1.83]	<0.001
REMOVE: WANG ET AL 2021_2	1.55 [1.32, 1.82]	<0.001

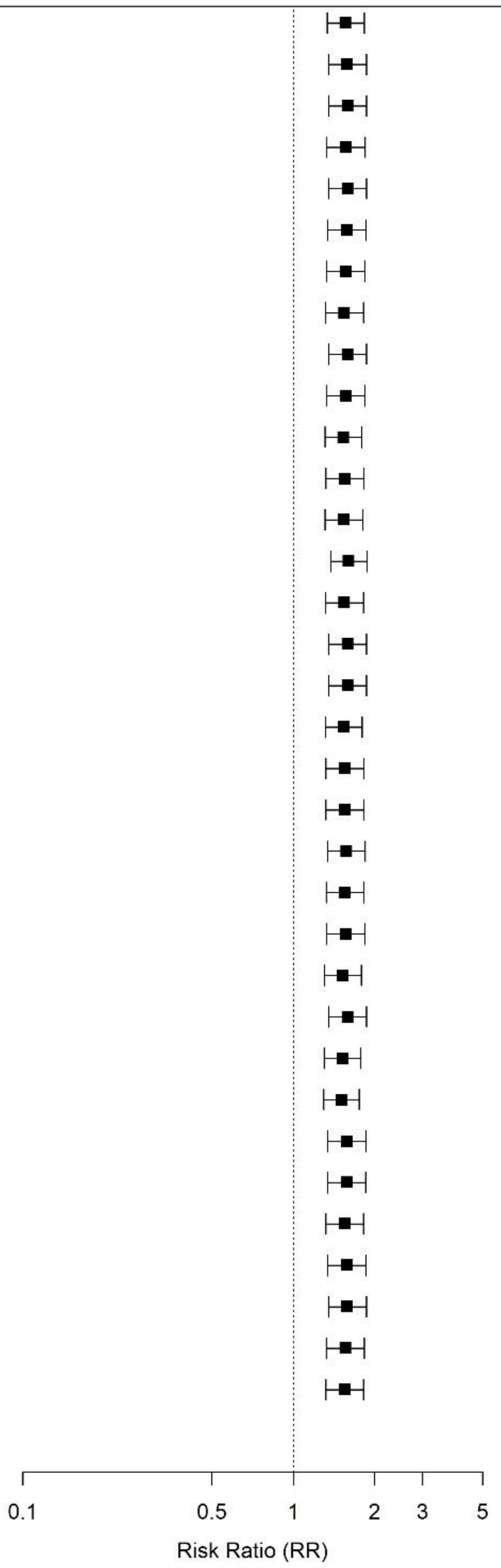

CV – cardiovascular; CI – confidence interval; I2 denotes the heterogeneity statistic.; RR – risk ratio

Supplementary Figure 21: Leave one out analyses for cardiovascular mortality within 1 year

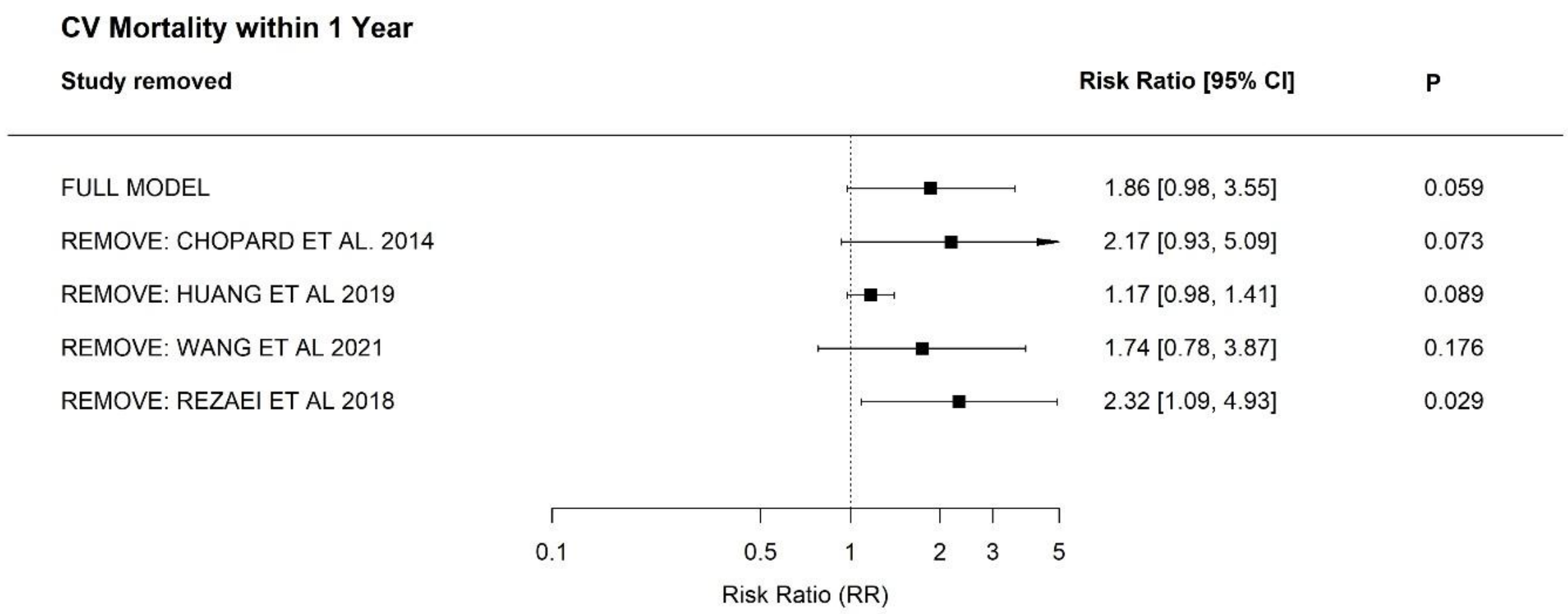

CV – cardiovascular; CI – confidence interval; I2 denotes the heterogeneity statistic.; RR – risk ratio

Supplementary Figure 22: Leave one out analyses for cardiovascular mortality over 1 year

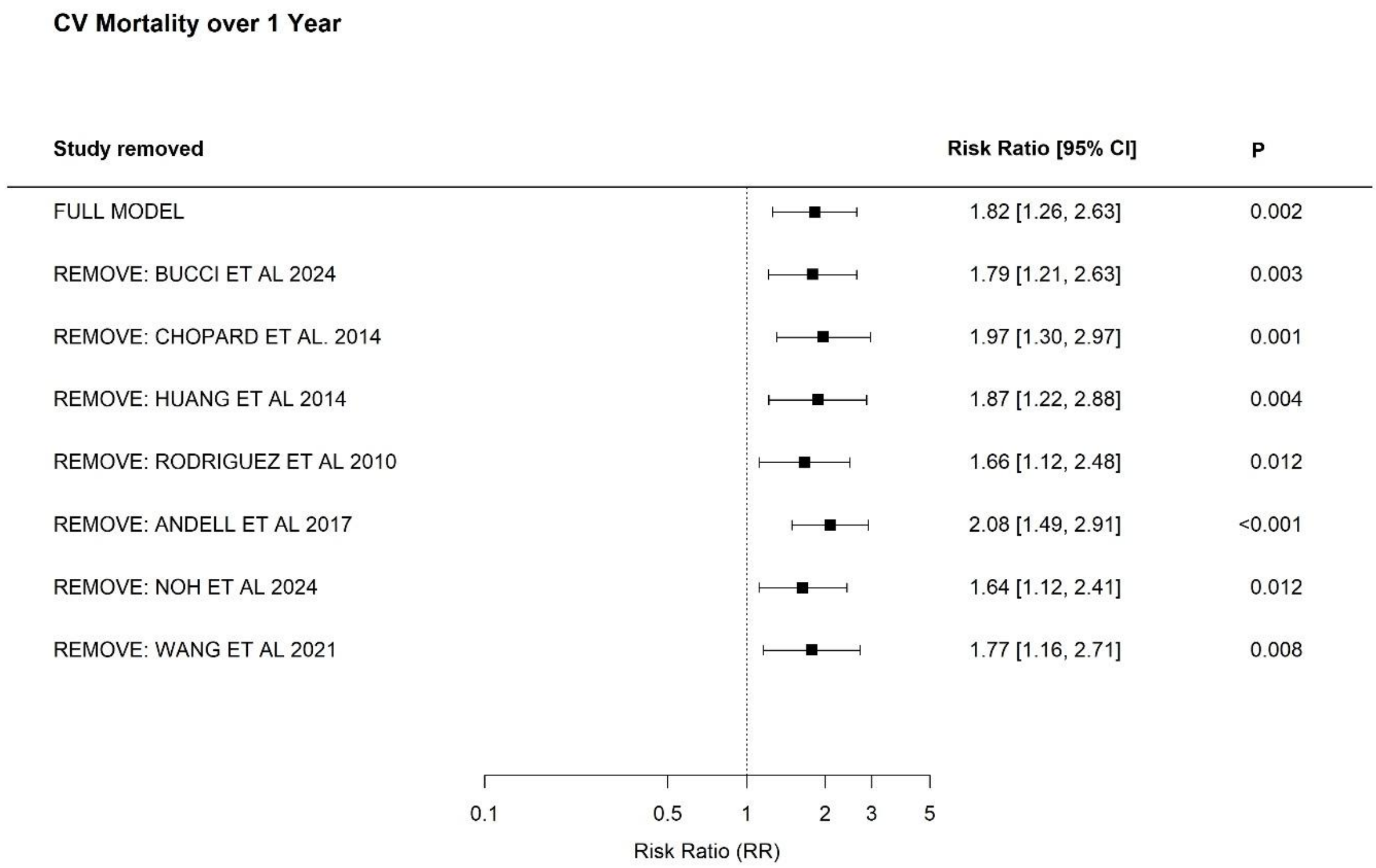

CV – cardiovascular; CI – confidence interval; I2 denotes the heterogeneity statistic.; RR – risk ratio

Supplementary Figure 23: Leave one out analyses for all-cause mortality

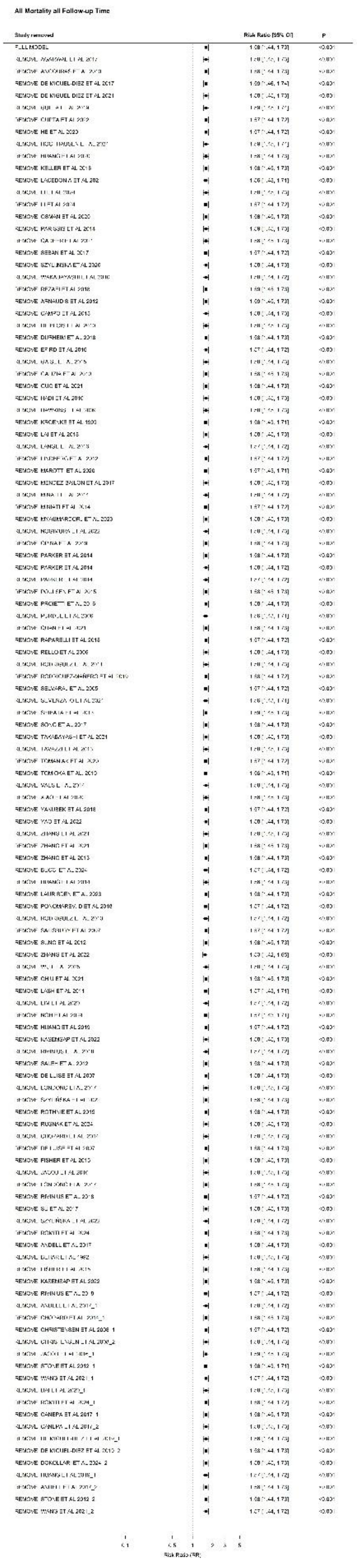

CV – cardiovascular; CI – confidence interval; I2 denotes the heterogeneity statistic.; RR – risk ratio

Supplementary Figure 24: Leave one out analyses for all-cause mortality within one year

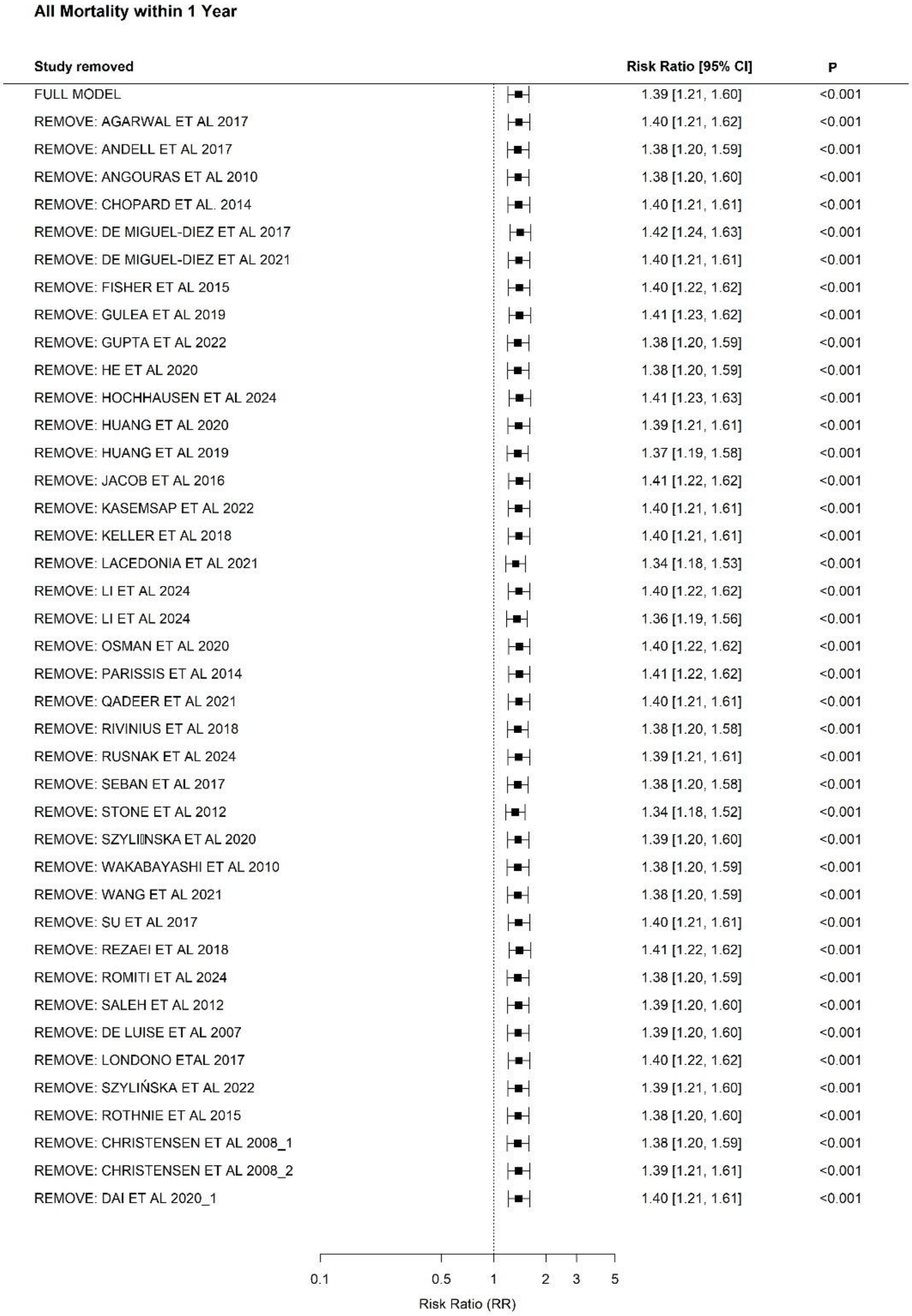

CV – cardiovascular; CI – confidence interval; I2 denotes the heterogeneity statistic.; RR – risk ratio

Supplementary Figure 25: Leave one out analyses for all-cause mortality over one year

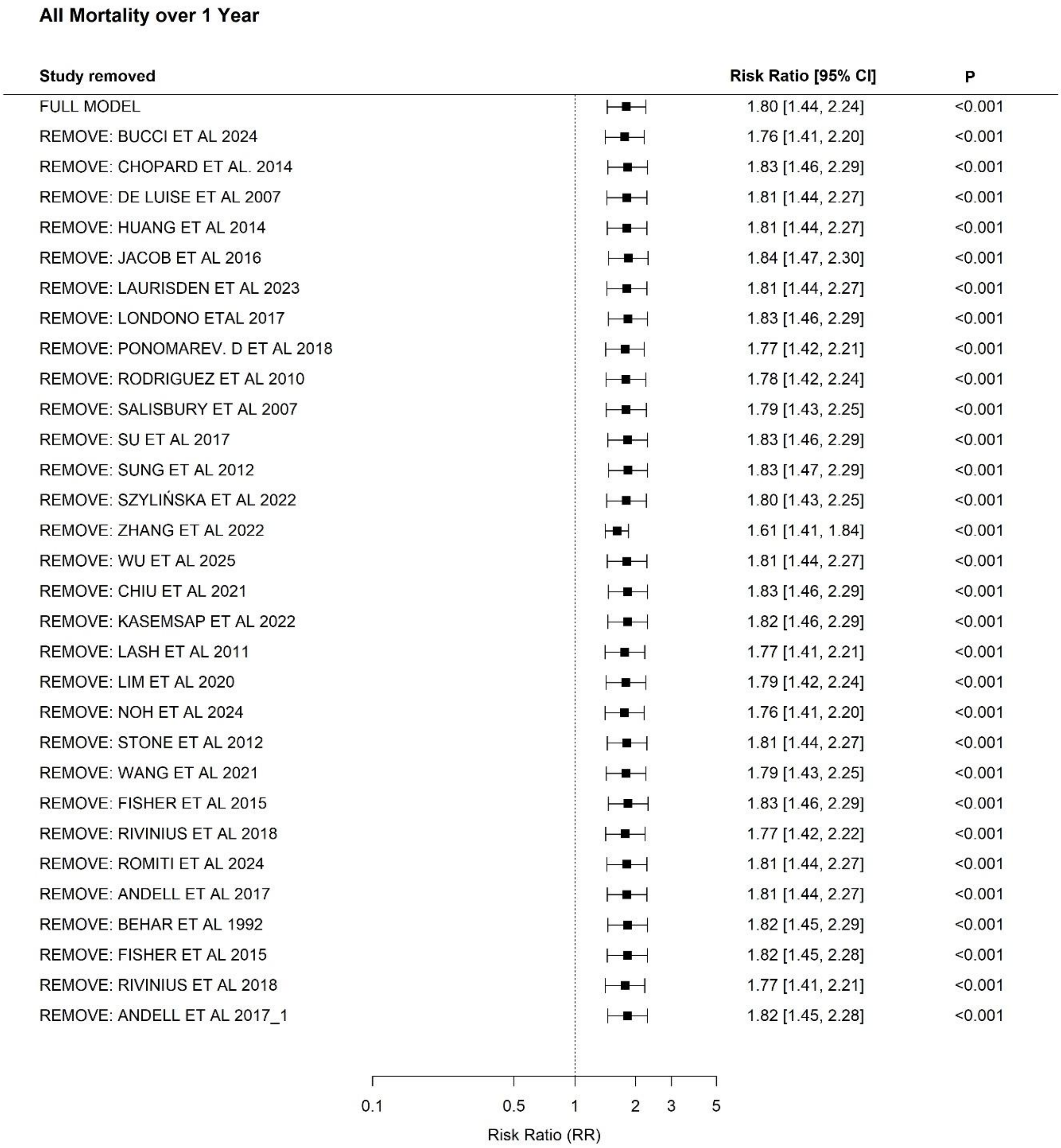

CV – cardiovascular; CI – confidence interval; I2 denotes the heterogeneity statistic.; RR – risk ratio

REFERENCES

1. Agarwal M, Agrawal S, Garg L, et al. Effect of Chronic Obstructive Pulmonary Disease on In-Hospital Mortality and Clinical Outcomes After ST-Segment Elevation Myocardial Infarction. *Am J Cardiol*. May 15 2017;119(10):1555-1559. doi:10.1016/j.amjcard.2017.02.024
2. Andell P, Sjogren J, Batra G, Szummer K, Koul S. Outcome of patients with chronic obstructive pulmonary disease and severe coronary artery disease who had a coronary artery bypass graft or a percutaneous coronary intervention. *Eur J Cardiothorac Surg*. Nov 1 2017;52(5):930-936. doi:10.1093/ejcts/ezx219
3. Angouras DC, Anagnostopoulos CE, Chamogeorgakis TP, et al. Postoperative and long-term outcome of patients with chronic obstructive pulmonary disease undergoing coronary artery bypass grafting. *Ann Thorac Surg*. Apr 2010;89(4):1112-8. doi:10.1016/j.athoracsur.2010.01.009
4. Behar S, Panosh A, Reicher-Reiss H, Zion M, Schlesinger Z, Goldbourt U. Prevalence and prognosis of chronic obstructive pulmonary disease among 5,839 consecutive patients with acute myocardial infarction. SPRINT Study Group. *Am J Med*. Dec 1992;93(6):637-41. doi:10.1016/0002-9343(92)90196-i
5. Bucci T, Romiti GF, Shantsila A, et al. Risk of Death and Cardiovascular Events in Asian Patients With Atrial Fibrillation and Chronic Obstructive Pulmonary Disease: A Report From the Prospective APHRS Registry. *J Am Heart Assoc*. Apr 2 2024;13(7):e032785. doi:10.1161/JAHA.123.032785
6. Buklioska-Ilievska D, Minov J, Kochovska-Kamchevska N, Gigovska I, Doneva A, Baloski M. Carotid Artery Disease and Lower Extremities Artery Disease in Patients with Chronic Obstructive Pulmonary Disease. *Open Access Maced J Med Sci*. Jul 15 2019;7(13):2102-2107. doi:10.3889/oamjms.2019.576
7. Buzea CA, Dan GA, Dan AR, et al. Deceleration and Acceleration Capacities in Risk Stratification for Arrhythmias in Patients With Chronic Obstructive Pulmonary Disease. *Am J Ther*. Jan/Feb 2017;24(1):e44-e51. doi:10.1097/MJT.0000000000000450
8. Campo G, Guastaroba P, Marzocchi A, et al. Impact of COPD on long-term outcome after ST-segment elevation myocardial infarction receiving primary percutaneous coronary intervention. *Chest*. Sep 2013;144(3):750-757. doi:10.1378/chest.12-2313
9. Canepa M, Straburzynska-Migaj E, Drozdz J, et al. Characteristics, treatments and 1-year prognosis of hospitalized and ambulatory heart failure patients with chronic obstructive pulmonary disease in the European Society of Cardiology Heart Failure Long-Term Registry. *Eur J Heart Fail*. Jan 2018;20(1):100-110. doi:10.1002/ejhf.964
10. Carter P, Lagan J, Fortune C, et al. Association of Cardiovascular Disease With Respiratory Disease. *J Am Coll Cardiol*. May 7 2019;73(17):2166-2177. doi:10.1016/j.jacc.2018.11.063
11. Chen CY, Liao KM. Chronic Obstructive Pulmonary Disease is associated with risk of Chronic Kidney Disease: A Nationwide Case-Cohort Study. *Sci Rep*. May 11 2016;6:25855. doi:10.1038/srep25855
12. Chiang CH, Jiang YC, Hung WT, et al. Impact of medications on outcomes in patients with acute myocardial infarction and chronic obstructive pulmonary disease: A nationwide cohort study. *J Chin Med Assoc*. Feb 1 2023;86(2):183-190. doi:10.1097/JCMA.0000000000000835
13. Chopard R, Meneveau N, Chocron S, et al. Impact of chronic obstructive pulmonary disease on Valve Academic Research Consortium-defined outcomes after transcatheter aortic valve implantation (from the FRANCE 2 Registry). *Am J Cardiol*. May 1 2014;113(9):1543-9. doi:10.1016/j.amjcard.2014.01.432

14. Cobb K, Kenyon J, Lu J, et al. COPD is associated with increased cardiovascular disease risk independent of phenotype. *Respirology*. Dec 2024;29(12):1047-1057. doi:10.1111/resp.14799
15. Crothers K, Nance RM, Whitney BM, et al. Chronic obstructive pulmonary disease and the risk for myocardial infarction by type in people with HIV. *AIDS*. Apr 1 2023;37(5):745-752. doi:10.1097/QAD.0000000000003465
16. Curkendall SM, DeLuise C, Jones JK, et al. Cardiovascular disease in patients with chronic obstructive pulmonary disease, Saskatchewan Canada cardiovascular disease in COPD patients. *Ann Epidemiol*. Jan 2006;16(1):63-70. doi:10.1016/j.annepidem.2005.04.008
17. de Lucas-Ramos P, Izquierdo-Alonso JL, Rodriguez-Gonzalez Moro JM, et al. Chronic obstructive pulmonary disease as a cardiovascular risk factor. Results of a case-control study (CONSISTE study). *Int J Chron Obstruct Pulmon Dis*. 2012;7:679-86. doi:10.2147/COPD.S36222
18. de Miguel-Diez J, Lopez-de-Andres A, Hernandez-Barrera V, et al. Impact of COPD on outcomes in hospitalized patients with community-acquired pneumonia: Analysis of the Spanish national hospital discharge database (2004-2013). *Eur J Intern Med*. Sep 2017;43:69-76. doi:10.1016/j.ejim.2017.06.008
19. Dokollari A, Sicouri S, Hosseinian L, et al. Periprocedural Risk Predictors Affecting Long-Term Prognosis in Patients With Chronic Obstructive Pulmonary Disease Undergoing Coronary Artery Bypass Grafting. *Tex Heart Inst J*. Mar 18 2024;51(1)doi:10.14503/THIJ-23-8199
20. Durheim MT, Holmes DN, Blanco RG, et al. Characteristics and outcomes of adults with chronic obstructive pulmonary disease and atrial fibrillation. *Heart*. Nov 2018;104(22):1850-1858. doi:10.1136/heartjnl-2017-312735
21. Efird JT, Griffin W, O'Neal WT, et al. Long-Term Survival after Cardiac Surgery in Patients with Chronic Obstructive Pulmonary Disease. *Am J Crit Care*. May 2016;25(3):266-76. doi:10.4037/ajcc2016119
22. Ferreira EJP, Cardoso L, de Matos CJO, et al. Cardiovascular Prognosis of Subclinical Chronic Obstructive Pulmonary Disease in Patients with Suspected or Confirmed Coronary Artery Disease. *Int J Chron Obstruct Pulmon Dis*. 2023;18:1899-1908. doi:10.2147/COPD.S410416
23. Finkelstein J, Cha E, Scharf SM. Chronic obstructive pulmonary disease as an independent risk factor for cardiovascular morbidity. *Int J Chron Obstruct Pulmon Dis*. 2009;4:337-49. doi:10.2147/copd.s6400
24. Pavarino FL, Tanenbaum MT, Figueroa AV, et al. Effects of Chronic Obstructive Pulmonary Disease on the Outcomes of Fenestrated-Branched Endovascular Aortic Aneurysm Repair. *Cardiovasc Intervent Radiol*. Nov 2024;47(11):1452-1460. doi:10.1007/s00270-024-03884-w
25. Gaisl T, Schlatzer C, Schwarz EI, et al. Coronary artery calcification, epicardial fat burden, and cardiovascular events in chronic obstructive pulmonary disease. *PLoS One*. 2015;10(5):e0126613. doi:10.1371/journal.pone.0126613
26. Grymonprez M, Vakaet V, Kavousi M, et al. Chronic obstructive pulmonary disease and the development of atrial fibrillation. *Int J Cardiol*. Feb 1 2019;276:118-124. doi:10.1016/j.ijcard.2018.09.056
27. Gulea C, Zakeri R, Quint JK. Impact of chronic obstructive pulmonary disease on readmission after hospitalization for acute heart failure: A nationally representative US cohort study. *Int J Cardiol*. Sep 1 2019;290:113-118. doi:10.1016/j.ijcard.2019.04.087
28. Hadi HA, Zubaid M, Al Mahmeed W, et al. Prevalence and prognosis of chronic obstructive pulmonary disease among 8167 Middle Eastern patients with acute coronary syndrome. *Clin Cardiol*. Apr 2010;33(4):228-35. doi:10.1002/clc.20751
29. Hawkins NM, Huang Z, Pieper KS, et al. Chronic obstructive pulmonary disease is an independent predictor of death but not atherosclerotic events in patients with myocardial

infarction: analysis of the Valsartan in Acute Myocardial Infarction Trial (VALIANT). *Eur J Heart Fail*. Mar 2009;11(3):292-8. doi:10.1093/eurjhf/hfp001
30. Houben-Wilke S, Jorres RA, Bals R, et al. Peripheral Artery Disease and Its Clinical Relevance in Patients with Chronic Obstructive Pulmonary Disease in the COPD and Systemic Consequences-Comorbidities Network Study. *Am J Respir Crit Care Med*. Jan 15 2017;195(2):189-197. doi:10.1164/rccm.201602-0354OC
31. Huang B, Yang Y, Zhu J, et al. Clinical characteristics and prognostic significance of chronic obstructive pulmonary disease in patients with atrial fibrillation: results from a multicenter atrial fibrillation registry study. *J Am Med Dir Assoc*. Aug 2014;15(8):576-81. doi:10.1016/j.jamda.2014.04.009
32. Huang X, Redfors B, Chen S, et al. Impact of chronic obstructive pulmonary disease on prognosis after percutaneous coronary intervention and bypass surgery for left main coronary artery disease: an analysis from the EXCEL trial. *Eur J Cardiothorac Surg*. Jun 1 2019;55(6):1144-1151. doi:10.1093/ejcts/ezy438
33. Ingebrigtsen TS, Marott JL, Vestbo J, Nordestgaard BG, Lange P. Coronary heart disease and heart failure in asthma, COPD and asthma-COPD overlap. *BMJ Open Respir Res*. Feb 2020;7(1)doi:10.1136/bmjresp-2019-000470
34. Joo H, Park J, Lee SD, Oh YM. Comorbidities of chronic obstructive pulmonary disease in Koreans: a population-based study. *J Korean Med Sci*. Aug 2012;27(8):901-6. doi:10.3346/jkms.2012.27.8.901
35. Kasemsap N, Jeerasuwannakul W, Tiamkao S, et al. Propensity Score Analysis of the Association between Chronic Obstructive Lung Disease and Stroke Outcome: Thailand's National Database. *Cerebrovasc Dis*. 2022;51(5):577-584. doi:10.1159/000521767
36. Keller K, Hobohm L, Munzel T, Ostad MA, Espinola-Klein C. Impact of chronic obstructive pulmonary disease on the outcomes of patients with peripheral artery disease. *Respir Med*. Feb 2019;147:1-6. doi:10.1016/j.rmed.2018.12.010
37. Kim MY, Boo S, Yoo M, Lee J, Kang NR. Impact of chronic kidney disease among Korean adults with chronic obstructive pulmonary disease. *Int Urol Nephrol*. Jul 2017;49(7):1225-1232. doi:10.1007/s11255-017-1572-4
38. Konecny T, Somers KR, Park JY, et al. Chronic obstructive pulmonary disease as a risk factor for ventricular arrhythmias independent of left ventricular function. *Heart Rhythm*. Jun 2018;15(6):832-838. doi:10.1016/j.hrthm.2017.09.042
39. Kong ZB, Wang XD, Shen SR, et al. Risk Prediction for Arrhythmias by Heart Rate Deceleration Runs in Patients with Chronic Obstructive Pulmonary Disease. *Int J Chron Obstruct Pulmon Dis*. 2020;15:585-593. doi:10.2147/COPD.S234470
40. Kroenke K, Lawrence VA, Theroux JF, Tuley MR, Hilsenbeck S. Postoperative complications after thoracic and major abdominal surgery in patients with and without obstructive lung disease. *Chest*. Nov 1993;104(5):1445-51. doi:10.1378/chest.104.5.1445
41. Kurmi OP, Li L, Davis KJ, et al. Excess risk of major vascular diseases associated with airflow obstruction: a 9-year prospective study of 0.5 million Chinese adults. *Int J Chron Obstruct Pulmon Dis*. 2018;13:855-865. doi:10.2147/COPD.S153641
42. Lange P, Mogelvang R, Marott JL, Vestbo J, Jensen JS. Cardiovascular morbidity in COPD: A study of the general population. *COPD*. Feb 2010;7(1):5-10. doi:10.3109/15412550903499506
43. Lash TL, Johansen MB, Christensen S, et al. Hospitalization rates and survival associated with COPD: a nationwide Danish cohort study. *Lung*. Feb 2011;189(1):27-35. doi:10.1007/s00408-010-9274-z
44. Lauridsen MD, Valentin JB, Strange JE, et al. Mortality in patients with chronic obstructive pulmonary disorder undergoing transcatheter aortic valve replacement: The importance of chronic obstructive pulmonary disease exacerbation. *Am Heart J*. Aug 2023;262:100-109. doi:10.1016/j.ahj.2023.04.016

45. Li R, Huddleston SJ, Prastein DJ. In-hospital outcome of type A aortic dissection repair in patients with chronic obstructive pulmonary disease: A population study of National Inpatient Sample from 2015 to 2020. *Cardiovasc Revasc Med*. Dec 2024;69:19-23. doi:10.1016/j.carrev.2024.06.013
46. Li R, Sidawy A, Nguyen BN. Thirty-day outcomes of endovascular repair of Stanford type B aortic dissection in patients with chronic obstructive pulmonary disease. *Vascular*. Nov 5 2024:17085381241298732. doi:10.1177/17085381241298732
47. Liao KM, Kuo LT, Lu HY. Increased risk of peripheral arterial occlusive diseases in patients with chronic obstructive pulmonary disease: a nationwide study in Taiwan. *Int J Chron Obstruct Pulmon Dis*. 2019;14:1455-1464. doi:10.2147/COPD.S202029
48. van Manen JG, Bindels PJ, CJ IJ, van der Zee JS, Bottema BJ, Schade E. Prevalence of comorbidity in patients with a chronic airway obstruction and controls over the age of 40. *J Clin Epidemiol*. Mar 2001;54(3):287-93. doi:10.1016/s0895-4356(01)00346-8
49. Manganas H, Lacasse Y, Bourgeois S, Perron J, Dagenais F, Maltais F. Postoperative outcome after coronary artery bypass grafting in chronic obstructive pulmonary disease. *Can Respir J*. Jan-Feb 2007;14(1):19-24. doi:10.1155/2007/378963
50. Michalopoulos A, Geroulanos S, Papadimitriou L, et al. Mild or moderate chronic obstructive pulmonary disease risk in elective coronary artery bypass grafting surgery. *World J Surg*. Dec 2001;25(12):1507-11. doi:10.1007/s00268-001-0161-x
51. Miniati M, Monti S, Pavlickova I, Bottai M. Survival in COPD: impact of lung dysfunction and comorbidities. *Medicine (Baltimore)*. Sep 2014;93(12):e76. doi:10.1097/MD.0000000000000076
52. Nagorni-Obradovic LM, Vukovic DS. The prevalence of COPD co-morbidities in Serbia: results of a national survey. *NPJ Prim Care Respir Med*. Jun 12 2014;24:14008. doi:10.1038/npjpcrm.2014.8
53. Noh E, Jeong H, Cho IS, et al. Risk of Cardiovascular Events Associated with Chronic Obstructive Pulmonary Disease and/or Metabolic Syndrome: A Large-Scale Nationwide Population-Based Cohort Study. *Int J Chron Obstruct Pulmon Dis*. 2024;19:1447-1456. doi:10.2147/COPD.S458779
54. Osman M, Khan MZ, Farjo PD, et al. In-hospital outcomes of percutaneous mitral valve repair in patients with chronic obstructive pulmonary disease: insights from the national inpatient sample database. *Catheter Cardiovasc Interv*. Jan 1 2021;97(1):E104-E112. doi:10.1002/ccd.28913
55. Parker DR, Liu J, Roberts MB, Eaton CB. Is inflammatory chronic obstructive pulmonary disease a coronary heart disease risk equivalent? A longitudinal analysis of the third National Health and Nutrition Examination Survey (NHANES III), 1988-1994. *BMC Pulm Med*. Dec 5 2014;14:195. doi:10.1186/1471-2466-14-195
56. Perticone M, Maio R, Caroleo B, et al. COPD significantly increases cerebral and cardiovascular events in hypertensives. *Sci Rep*. Apr 12 2021;11(1):7884. doi:10.1038/s41598-021-86963-z
57. Poulsen MK, Dahl JS, Kjeldsen BJ, et al. Impact of chronic obstructive pulmonary disease on survival and symptoms of severe aortic valve stenosis. *Scand Cardiovasc J*. Feb 2015;49(1):49-55. doi:10.3109/14017431.2015.1006671
58. Proietti M, Laroche C, Drozd M, et al. Impact of chronic obstructive pulmonary disease on prognosis in atrial fibrillation: A report from the EURObservational Research Programme Pilot Survey on Atrial Fibrillation (EORP-AF) General Registry. *Am Heart J*. Nov 2016;181:83-91. doi:10.1016/j.ahj.2016.08.011
59. Qadeer A, Parikh PB, Ramkishun CA, Tai J, Patel JK. Impact of chronic obstructive pulmonary disease on survival and neurologic outcomes in adults with in-hospital cardiac arrest. *PLoS One*. 2021;16(11):e0259698. doi:10.1371/journal.pone.0259698

60. Raparelli V, Pastori D, Pignataro SF, et al. Major adverse cardiovascular events in non-valvular atrial fibrillation with chronic obstructive pulmonary disease: the ARAPACIS study. *Intern Emerg Med*. Aug 2018;13(5):651-660. doi:10.1007/s11739-018-1835-9
61. Rezaei SS, Rinner C, Ratajczak P, Grossmann W, Gall W, Wolzt M. Use of beta-blocker is associated with lower mortality in patients with coronary artery disease with or without COPD. *Clin Respir J*. Dec 2018;12(12):2627-2634. doi:10.1111/crj.12968
62. Rivinius R, Helmschrott M, Ruhparwar A, et al. COPD in patients after heart transplantation is associated with a prolonged hospital stay, early posttransplant atrial fibrillation, and impaired posttransplant survival. *Clin Epidemiol*. 2018;10:1359-1369. doi:10.2147/CLEP.S171929
63. Rodriguez A, Lisboa T, Sole-Violan J, et al. Impact of nonexacerbated COPD on mortality in critically ill patients. *Chest*. Jun 2011;139(6):1354-1360. doi:10.1378/chest.10-2439
64. Rodriguez LA, Wallander MA, Martin-Merino E, Johansson S. Heart failure, myocardial infarction, lung cancer and death in COPD patients: a UK primary care study. *Respir Med*. Nov 2010;104(11):1691-9. doi:10.1016/j.rmed.2010.04.018
65. Rusnak J, Behnes M, Schupp T, et al. COPD increases cardiac mortality in patients presenting with ventricular tachyarrhythmias and aborted cardiac arrest. *Respir Med*. Dec 2018;145:153-160. doi:10.1016/j.rmed.2018.10.019
66. Saleh HZ, Mohan K, Shaw M, et al. Impact of chronic obstructive pulmonary disease severity on surgical outcomes in patients undergoing non-emergent coronary artery bypass grafting. *Eur J Cardiothorac Surg*. Jul 2012;42(1):108-13; discussion 113. doi:10.1093/ejcts/ezr271
67. Schneider C, Bothner U, Jick SS, Meier CR. Chronic obstructive pulmonary disease and the risk of cardiovascular diseases. *Eur J Epidemiol*. Apr 2010;25(4):253-60. doi:10.1007/s10654-010-9435-7
68. Serban RC, Hadadi L, Sus I, Lakatos EK, Demjen Z, Scridon A. Impact of chronic obstructive pulmonary disease on in-hospital morbidity and mortality in patients with ST-segment elevation myocardial infarction treated by primary percutaneous coronary intervention. *Int J Cardiol*. Sep 15 2017;243:437-442. doi:10.1016/j.ijcard.2017.05.044
69. Shen AL, Lin HL, Lin HC, Chao JC, Hsu CY, Chen CY. The effects of medications for treating COPD and allied conditions on stroke: a population-based cohort study. *NPJ Prim Care Respir Med*. Jan 17 2022;32(1):4. doi:10.1038/s41533-021-00267-3
70. Shibata Y, Inoue S, Igarashi A, et al. A lower level of forced expiratory volume in 1 second is a risk factor for all-cause and cardiovascular mortality in a Japanese population: the Takahata study. *PLoS One*. 2013;8(12):e83725. doi:10.1371/journal.pone.0083725
71. Sidney S, Sorel M, Quesenberry CP, Jr., DeLuise C, Lanes S, Eisner MD. COPD and incident cardiovascular disease hospitalizations and mortality: Kaiser Permanente Medical Care Program. *Chest*. Oct 2005;128(4):2068-75. doi:10.1378/chest.128.4.2068
72. Skajaa N, Laugesen K, Horvath-Puho E, Sorensen HT. Comorbidities and mortality among patients with chronic obstructive pulmonary disease. *BMJ Open Respir Res*. Oct 2023;10(1)doi:10.1136/bmjresp-2023-001798
73. Sode BF, Dahl M, Nordestgaard BG. Myocardial infarction and other co-morbidities in patients with chronic obstructive pulmonary disease: a Danish nationwide study of 7.4 million individuals. *Eur Heart J*. Oct 2011;32(19):2365-75. doi:10.1093/eurheartj/ehr338
74. Soderholm M, Inghammar M, Hedblad B, Egesten A, Engstrom G. Incidence of stroke and stroke subtypes in chronic obstructive pulmonary disease. *Eur J Epidemiol*. Feb 2016;31(2):159-68. doi:10.1007/s10654-015-0113-7
75. Song S, Yang PS, Kim TH, et al. Relation of Chronic Obstructive Pulmonary Disease to Cardiovascular Disease in the General Population. *Am J Cardiol*. Oct 15 2017;120(8):1399-1404. doi:10.1016/j.amjcard.2017.07.032

76. Soumagne T, Guillien A, Roche N, et al. In Patients with Mild-to-Moderate COPD, Tobacco Smoking, and Not COPD, Is Associated with a Higher Risk of Cardiovascular Comorbidity. *Int J Chron Obstruct Pulmon Dis*. 2020;15:1545-1555. doi:10.2147/COPD.S253417
77. Stallberg B, Janson C, Larsson K, et al. Real-world retrospective cohort study ARCTIC shows burden of comorbidities in Swedish COPD versus non-COPD patients. *NPJ Prim Care Respir Med*. Sep 10 2018;28(1):33. doi:10.1038/s41533-018-0101-y
78. Sung PH, Chung SY, Sun CK, et al. Impact of chronic obstructive pulmonary disease on patient with acute myocardial infarction undergoing primary percutaneous coronary intervention. *Biomed J*. Nov-Dec 2013;36(6):274-81. doi:10.4103/2319-4170.113373
79. Szylinska A, Kotfis K, Listewnik M, Brykczynski M, Marra A, Rotter I. The burden of chronic obstructive pulmonary disease in open heart surgery-a retrospective cohort analysis of postoperative complications: STROBE compliant. *Medicine (Baltimore)*. Mar 2020;99(13):e19675. doi:10.1097/MD.0000000000019675
80. Szylinska A, Kotfis K, Bott-Olejnik M, Wankowicz P, Rotter I. Post-Stroke Outcomes of Patients with Chronic Obstructive Pulmonary Disease. *Brain Sci*. Jan 13 2022;12(1)doi:10.3390/brainsci12010106
81. Takabayashi K, Terasaki Y, Okuda M, et al. The clinical characteristics and outcomes of heart failure patient with chronic obstructive pulmonary disease from the Japanese community-based registry. *Heart Vessels*. Feb 2021;36(2):223-234. doi:10.1007/s00380-020-01675-0
82. Tavazzi L, Swedberg K, Komajda M, et al. Clinical profiles and outcomes in patients with chronic heart failure and chronic obstructive pulmonary disease: an efficacy and safety analysis of SHIFT study. *Int J Cardiol*. Dec 10 2013;170(2):182-8. doi:10.1016/j.ijcard.2013.10.068
83. Terzikhan N, Lahousse L, Verhamme KMC, et al. COPD is associated with an increased risk of peripheral artery disease and mortality. *ERJ Open Res*. Oct 2018;4(4)doi:10.1183/23120541.00086-2018
84. Tomaniak M, Chichareon P, Takahashi K, et al. Impact of chronic obstructive pulmonary disease and dyspnoea on clinical outcomes in ticagrelor treated patients undergoing percutaneous coronary intervention in the randomized GLOBAL LEADERS trial. *Eur Heart J Cardiovasc Pharmacother*. Jul 1 2020;6(4):222-230. doi:10.1093/ehjcvp/pvz052
85. Tomioka T, Fukui K, Tanaka S, et al. Influence of atrial fibrillation on cardiac prognosis in chronic obstructive pulmonary disease. *Indian Heart J*. Jan-Feb 2019;71(1):7-11. doi:10.1016/j.ihj.2018.11.009
86. Wakabayashi K, Gonzalez MA, Delhaye C, et al. Impact of chronic obstructive pulmonary disease on acute-phase outcome of myocardial infarction. *Am J Cardiol*. Aug 1 2010;106(3):305-9. doi:10.1016/j.amjcard.2010.03.026
87. Yakubek GA, Curtis GL, Khlopas A, et al. Chronic Obstructive Pulmonary Disease Is Associated With Short-Term Complications Following Total Knee Arthroplasty. *J Arthroplasty*. Aug 2018;33(8):2623-2626. doi:10.1016/j.arth.2018.03.011
88. Yang K, Wu Y, Chen D, Liu S, Chen R. The Impact of Lung Function on Extra-Pulmonary Diseases and All-Cause Mortality in US Adult Population with and without COPD. *Clin Epidemiol*. 2020;12:997-1005. doi:10.2147/CLEP.S270599
89. Yao Y, Zhu P, Xu N, et al. Effects of chronic obstructive pulmonary disease on long-term prognosis of patients with coronary heart disease post-percutaneous coronary intervention. *J Geriatr Cardiol*. Jun 28 2022;19(6):428-434. doi:10.11909/j.issn.1671-5411.2022.06.005
90. Yoshizawa T, Okada K, Furuichi S, et al. Prevalence of chronic kidney diseases in patients with chronic obstructive pulmonary disease: assessment based on glomerular filtration rate estimated from creatinine and cystatin C levels. *Int J Chron Obstruct Pulmon Dis*. 2015;10:1283-9. doi:10.2147/COPD.S80673
91. Zafiraki VK, Kosmacheva ED, Mirzaev SG, et al. Chronic obstructive pulmonary disease in patients with coronary heart disease worsens long-term prognosis after percutaneous

coronary interventions. *Kardiologiia*. Nov 30 2021;61(11):24-32. doi:10.18087/cardio.2021.11.n1820
92. Zhang JW, Zhou YJ, Yang Q, Yang SW, Nie B, Xu XH. Impact of chronic obstructive pulmonary diseases on outcomes and hospital days after percutaneous coronary intervention. *Angiology*. Aug 2013;64(6):430-4. doi:10.1177/0003319712458145
93. Al-Khatib SM, Granger CB, Huang Y, et al. Sustained ventricular arrhythmias among patients with acute coronary syndromes with no ST-segment elevation: incidence, predictors, and outcomes. *Circulation*. Jul 16 2002;106(3):309-12. doi:10.1161/01.cir.0000022692.49934.e3
94. Andell P, James SK, Cannon CP, et al. Ticagrelor Versus Clopidogrel in Patients With Acute Coronary Syndromes and Chronic Obstructive Pulmonary Disease: An Analysis From the Platelet Inhibition and Patient Outcomes (PLATO) Trial. *J Am Heart Assoc*. Oct 9 2015;4(10):e002490. doi:10.1161/JAHA.115.002490
95. Angeli F, Reboldi G, Trapasso M, Aita A, Ambrosio G, Verdecchia P. Detrimental Impact of Chronic Obstructive Pulmonary Disease in Atrial Fibrillation: New Insights from Umbria Atrial Fibrillation Registry. *Medicina (Kaunas)*. Jul 9 2019;55(7)doi:10.3390/medicina55070358
96. Baty F, Putora PM, Isenring B, Blum T, Brutsche M. Comorbidities and burden of COPD: a population based case-control study. *PLoS One*. 2013;8(5):e63285. doi:10.1371/journal.pone.0063285
97. Bursi F, Vassallo R, Weston SA, Killian JM, Roger VL. Chronic obstructive pulmonary disease after myocardial infarction in the community. *Am Heart J*. Jul 2010;160(1):95-101. doi:10.1016/j.ahj.2010.05.004
98. Castagna O, Boussuges A, Nussbaum E, Marqueste L, Brisswalter J. Peripheral arterial disease: an underestimated aetiology of exercise intolerance in chronic obstructive pulmonary disease patients. *Eur J Cardiovasc Prev Rehabil*. Jun 2008;15(3):270-7. doi:10.1097/HJR.0b013e3282f009a9
99. Cave A, Pham A, Lindeman C, Soos B, Williamson T, Drummond N. Chronic obstructive pulmonary disease as a risk factor in primary care: a Canadian retrospective cohort study. *NPJ Prim Care Respir Med*. Jun 24 2021;31(1):37. doi:10.1038/s41533-021-00249-5
100. Chen CC, Lin CH, Hao WR, et al. Association between chronic obstructive pulmonary disease and ventricular arrhythmia: a nationwide population-based cohort study. *NPJ Prim Care Respir Med*. Feb 12 2021;31(1):8. doi:10.1038/s41533-021-00221-3
101. Cohen A, Katz M, Katz R, Hauptman E, Schachner A. Chronic obstructive pulmonary disease in patients undergoing coronary artery bypass grafting. *J Thorac Cardiovasc Surg*. Mar 1995;109(3):574-81. doi:10.1016/S0022-5223(95)70291-1
102. Cuthbert JJ, Kearsley JW, Kazmi S, et al. The impact of heart failure and chronic obstructive pulmonary disease on mortality in patients presenting with breathlessness. *Clin Res Cardiol*. Feb 2019;108(2):185-193. doi:10.1007/s00392-018-1342-z
103. De Luca G, Nardin M, Algowhary M, et al. Impact of chronic obstructive pulmonary disease on short-term outcome in patients with ST-elevation myocardial infarction during COVID-19 pandemic: insights from the international multicenter ISACS-STEMI registry. *Respir Res*. Aug 15 2022;23(1):207. doi:10.1186/s12931-022-02128-0
104. de Miguel-Diez J, Lopez-de-Andres A, Hernandez-Barrera V, de Miguel-Yanes JM, Mendez-Bailon M, Jimenez-Garcia R. Trends and hospital outcomes of lung transplantation among patients with and without chronic obstructive pulmonary disease in Spain: a national population-based study (2001-2015). *Int J Chron Obstruct Pulmon Dis*. 2019;14:729-737. doi:10.2147/COPD.S189010
105. de-Miguel-Diez J, Lopez-de-Andres A, Hernandez-Barrera V, et al. Trends, characteristics, in-hospital outcomes and mortality in surgical mitral valve replacement among patients with and without COPD in Spain (2001-2015). *PLoS One*. 2019;14(8):e0221263. doi:10.1371/journal.pone.0221263

106. de-Miguel-Diez J, Albaladejo-Vicente R, Jimenez-Garcia R, et al. The effect of COPD on the incidence and mortality of hospitalized patients with pulmonary embolism: A nationwide population-based study (2016-2018). *Eur J Intern Med*. Feb 2021;84:18-23. doi:10.1016/j.ejim.2020.11.009
107. Distelmaier K, Niessner A, Haider D, et al. Long-term mortality in patients with chronic obstructive pulmonary disease following extracorporeal membrane oxygenation for cardiac assist after cardiovascular surgery. *Intensive Care Med*. Aug 2013;39(8):1444-51. doi:10.1007/s00134-013-2931-y
108. Eriksson B, Backman H, Ekerljung L, et al. Pattern of Cardiovascular Comorbidity in COPD in a Country with Low-smoking Prevalence: Results from Two-population-based Cohorts from Sweden. *COPD*. Oct 2018;15(5):454-463. doi:10.1080/15412555.2018.1535580
109. Feary JR, Rodrigues LC, Smith CJ, Hubbard RB, Gibson JE. Prevalence of major comorbidities in subjects with COPD and incidence of myocardial infarction and stroke: a comprehensive analysis using data from primary care. *Thorax*. Nov 2010;65(11):956-62. doi:10.1136/thx.2009.128082
110. Funk GC, Bauer P, Burghuber OC, et al. Prevalence and prognosis of COPD in critically ill patients between 1998 and 2008. *Eur Respir J*. Apr 2013;41(4):792-9. doi:10.1183/09031936.00226411
111. Garcia-Olmos L, Alberquilla A, Ayala V, et al. Comorbidity in patients with chronic obstructive pulmonary disease in family practice: a cross sectional study. *BMC Fam Pract*. Jan 16 2013;14:11. doi:10.1186/1471-2296-14-11
112. Groenewegen A, Zwartkruis VW, Smit LJ, et al. Sex-specific and age-specific incidence of ischaemic heart disease, atrial fibrillation and heart failure in community patients with chronic obstructive pulmonary disease. *BMJ Open Respir Res*. Dec 2022;9(1)doi:10.1136/bmjresp-2022-001307
113. Hong Y, Graham MM, Southern D, McMurtry MS. The Association between Chronic Obstructive Pulmonary Disease and Coronary Artery Disease in Patients Undergoing Coronary Angiography. *COPD*. Feb 2019;16(1):66-71. doi:10.1080/15412555.2019.1566894
114. Kang HR, Kim SJ, Nam JG, Park YS, Lee CH. Impact of Smoking and Chronic Obstructive Pulmonary Disease on All-Cause, Respiratory, and Cardio-Cerebrovascular Mortality. *Int J Chron Obstruct Pulmon Dis*. 2024;19:1261-1272. doi:10.2147/COPD.S458356
115. Lazzeri C, Valente S, Attana P, Chiostri M, Picariello C, Gensini GF. The prognostic role of chronic obstructive pulmonary disease in ST-elevation myocardial infarction after primary angioplasty. *Eur J Prev Cardiol*. Jun 2013;20(3):392-8. doi:10.1177/1741826711428243
116. Liao KM, Lu HY. A National Analysis of Complications Following Total Hip Replacement in Patients With Chronic Obstructive Pulmonary Disease. *Medicine (Baltimore)*. Mar 2016;95(12):e3182. doi:10.1097/MD.0000000000003182
117. Lim JU, Yeo CD, Rhee CK, et al. Overall survival of driver mutation-negative non-small cell lung cancer patients with COPD under chemotherapy compared to non-COPD non-small cell lung cancer patients. *Int J Chron Obstruct Pulmon Dis*. 2018;13:2139-2146. doi:10.2147/COPD.S167372
118. Makris D, Desrousseaux B, Zakynthinos E, Durocher A, Nseir S. The impact of COPD on ICU mortality in patients with ventilator-associated pneumonia. *Respir Med*. Jul 2011;105(7):1022-9. doi:10.1016/j.rmed.2011.03.001
119. Mapel DW, Dedrick D, Davis K. Trends and cardiovascular co-morbidities of COPD patients in the Veterans Administration Medical System, 1991-1999. *COPD*. Mar 2005;2(1):35-41. doi:10.1081/copd-200050671
120. Navaneethan SD, Schold JD, Huang H, et al. Mortality Outcomes of Patients with Chronic Kidney Disease and Chronic Obstructive Pulmonary Disease. *Am J Nephrol*. 2016;43(1):39-46. doi:10.1159/000444422

121. Nilsson U, Blomberg A, Johansson B, Backman H, Eriksson B, Lindberg A. Ischemic ECG abnormalities are associated with an increased risk for death among subjects with COPD, also among those without known heart disease. *Int J Chron Obstruct Pulmon Dis*. 2017;12:2507-2514. doi:10.2147/COPD.S136404
122. Nishiyama K, Morimoto T, Furukawa Y, et al. Chronic obstructive pulmonary disease--an independent risk factor for long-term cardiac and cardiovascular mortality in patients with ischemic heart disease. *Int J Cardiol*. Aug 20 2010;143(2):178-83. doi:10.1016/j.ijcard.2009.02.010
123. Omote N, Hashimoto N, Morise M, et al. Impact of mild to moderate COPD on feasibility and prognosis in non-small cell lung cancer patients who received chemotherapy. *Int J Chron Obstruct Pulmon Dis*. 2017;12:3541-3547. doi:10.2147/COPD.S149456
124. Perez-Padilla R, Wehrmeister FC, de Oca MM, et al. Outcomes for symptomatic non-obstructed individuals and individuals with mild (GOLD stage 1) COPD in a population based cohort. *Int J Chron Obstruct Pulmon Dis*. 2018;13:3549-3561. doi:10.2147/COPD.S175527
125. Portegies ML, Lahousse L, Joos GF, et al. Chronic Obstructive Pulmonary Disease and the Risk of Stroke. The Rotterdam Study. *Am J Respir Crit Care Med*. Feb 1 2016;193(3):251-8. doi:10.1164/rccm.201505-0962OC
126. Putcha N, Han MK, Martinez CH, et al. Comorbidities of COPD have a major impact on clinical outcomes, particularly in African Americans. *Chronic Obstr Pulm Dis*. 2014;1(1):105-114. doi:10.15326/jcopdf.1.1.2014.0112
127. Raslan AS, Quint JK, Cook S. All-Cause, Cardiovascular and Respiratory Mortality in People with Type 2 Diabetes and Chronic Obstructive Pulmonary Disease (COPD) in England: A Cohort Study Using the Clinical Practice Research Datalink (CPRD). *Int J Chron Obstruct Pulmon Dis*. 2023;18:1207-1218. doi:10.2147/COPD.S407085
128. Restrepo MI, Mortensen EM, Pugh JA, Anzueto A. COPD is associated with increased mortality in patients with community-acquired pneumonia. *Eur Respir J*. Aug 2006;28(2):346-51. doi:10.1183/09031936.06.00131905
129. Sankar A, Thorpe K, McIsaac DI, Luo J, Wijeysundera DN, Gershon AS. Survival and health care costs after inpatient elective surgery: comparison of patients with and without chronic obstructive pulmonary disease. *CMAJ*. Jan 17 2023;195(2):E62-E71. doi:10.1503/cmaj.220733
130. Scrutinio D, Guida P, Passantino A, et al. Acutely decompensated heart failure with chronic obstructive pulmonary disease: Clinical characteristics and long-term survival. *Eur J Intern Med*. Feb 2019;60:31-38. doi:10.1016/j.ejim.2018.11.002
131. Soriano JB, Visick GT, Muellerova H, Payvandi N, Hansell AL. Patterns of comorbidities in newly diagnosed COPD and asthma in primary care. *Chest*. Oct 2005;128(4):2099-107. doi:10.1378/chest.128.4.2099
132. Stefan MS, Bannuru RR, Lessard D, Gore JM, Lindenauer PK, Goldberg RJ. The impact of COPD on management and outcomes of patients hospitalized with acute myocardial infarction: a 10-year retrospective observational study. *Chest*. Jun 2012;141(6):1441-1448. doi:10.1378/chest.11-2032
133. van Gestel YR, Chonchol M, Hoeks SE, et al. Association between chronic obstructive pulmonary disease and chronic kidney disease in vascular surgery patients. *Nephrol Dial Transplant*. Sep 2009;24(9):2763-7. doi:10.1093/ndt/gfp171
134. Vogelmeier CF, Friedrich FW, Timpel P, et al. Impact of COPD on mortality: An 8-year observational retrospective healthcare claims database cohort study. *Respir Med*. Feb 2024;222:107506. doi:10.1016/j.rmed.2023.107506
135. Yang L, Zhang Y, Yao W, Fang F, Li W. Impact of Chronic Obstructive Pulmonary Disease on Infectious Complications and Mortality in Patients With Aneurysmal Subarachnoid Hemorrhage. *Front Neurol*. 2021;12:723115. doi:10.3389/fneur.2021.723115

136. Arnaudis B, Lairez O, Escamilla R, et al. Impact of chronic obstructive pulmonary disease severity on symptoms and prognosis in patients with systolic heart failure. *Clin Res Cardiol*. Sep 2012;101(9):717-26. doi:10.1007/s00392-012-0450-4
137. Chiu KC, Lin WC, Chang CL, Wu SY. Impact of Chronic Obstruction Pulmonary Disease on Survival in Patients with Advanced Stage Lung Squamous Cell Carcinoma Undergoing Concurrent Chemoradiotherapy. *Cancers (Basel)*. Jun 28 2021;13(13)doi:10.3390/cancers13133231
138. Christensen S, Thomsen RW, Torring ML, Riis A, Norgaard M, Sorensen HT. Impact of COPD on outcome among patients with complicated peptic ulcer. *Chest*. Jun 2008;133(6):1360-1366. doi:10.1378/chest.07-2543
139. Dai Y, Qin S, Pan H, Chen T, Bian D. Impacts of Comorbid Chronic Obstructive Pulmonary Disease and Congestive Heart Failure on Prognosis of Critically Ill Patients. *Int J Chron Obstruct Pulmon Dis*. 2020;15:2707-2714. doi:10.2147/COPD.S275573
140. De Blois J, Simard S, Atar D, Agewall S, Norwegian Heart Failure R. COPD predicts mortality in HF: the Norwegian Heart Failure Registry. *J Card Fail*. Mar 2010;16(3):225-9. doi:10.1016/j.cardfail.2009.12.002
141. de Luise C, Brimacombe M, Pedersen L, Sorensen HT. Chronic obstructive pulmonary disease and mortality following hip fracture: a population-based cohort study. *Eur J Epidemiol*. 2008;23(2):115-22. doi:10.1007/s10654-007-9211-5
142. de Miguel-Diez J, Lopez-de-Andres A, Hernandez-Barrera V, Mendez-Bailon M, de Miguel-Yanes JM, Jimenez-Garcia R. Impact of COPD on outcomes in hospitalized patients treated with transcatheter aortic valve implantation or surgical aortic valve replacement in Spain. *Catheter Cardiovasc Interv*. Feb 2020;95(2):339-347. doi:10.1002/ccd.28321
143. de Miguel-Diez J, Lopez-de-Andres A, Jimenez-Garcia R, et al. Sex Differences in the Incidence and Outcomes of COPD Patients Hospitalized with Ischemic Stroke in Spain: A Population-Based Observational Study. *Int J Chron Obstruct Pulmon Dis*. 2021;16:1851-1862. doi:10.2147/COPD.S311826
144. Fisher KA, Stefan MS, Darling C, Lessard D, Goldberg RJ. Impact of COPD on the mortality and treatment of patients hospitalized with acute decompensated heart failure: the Worcester Heart Failure Study. *Chest*. Mar 2015;147(3):637-645. doi:10.1378/chest.14-0607
145. Galizia G, Cacciatore F, Testa G, et al. Role of clinical frailty on long-term mortality of elderly subjects with and without chronic obstructive pulmonary disease. *Aging Clin Exp Res*. Apr 2011;23(2):118-25. doi:10.1007/BF03351076
146. Guo C, Yu T, Chang LY, et al. Mortality risk attributable to classification of chronic obstructive pulmonary disease and reduced lung function: A 21-year longitudinal cohort study. *Respir Med*. Aug 2021;184:106471. doi:10.1016/j.rmed.2021.106471
147. Gupta P, Quan T, Abdo MG, Manzi JE, Knapp B, Shaffer G. Thirty-Day Morbidity and Mortality in Patients With COPD Following Open Reduction and Internal Fixation for Rotational Ankle Fractures. *J Foot Ankle Surg*. Nov-Dec 2022;61(6):1275-1279. doi:10.1053/j.jfas.2022.03.015
148. He Y, Xie M, Zhao J, Liu X. Clinical Characteristics and Outcomes of Patients with Severe COVID-19 and Chronic Obstructive Pulmonary Disease (COPD). *Med Sci Monit*. Sep 4 2020;26:e927212. doi:10.12659/MSM.927212
149. Hochhausen N, Mechelinck M, Billig S, Rossaint R, Kork F. Association between chronic obstructive pulmonary disease and in-hospital mortality after percutaneous coronary intervention: a retrospective cohort study in Germany. *Sci Rep*. Mar 13 2024;14(1):6044. doi:10.1038/s41598-024-56255-3
150. Huang W, Xie R, Hong Y, Chen Q. Association Between Comorbid Chronic Obstructive Pulmonary Disease and Prognosis of Patients Admitted to the Intensive Care Unit for Non-COPD Reasons: A Retrospective Cohort Study. *Int J Chron Obstruct Pulmon Dis*. 2020;15:279-287. doi:10.2147/COPD.S244020

151. Jacob J, Tost J, Miro O, et al. Impact of chronic obstructive pulmonary disease on clinical course after an episode of acute heart failure. EAHFE-COPD study. *Int J Cardiol*. Jan 15 2017;227:450-456. doi:10.1016/j.ijcard.2016.11.013
152. Lacedonia D, Scioscia G, Santomasi C, et al. Impact of smoking, COPD and comorbidities on the mortality of COVID-19 patients. *Sci Rep*. Sep 28 2021;11(1):19251. doi:10.1038/s41598-021-98749-4
153. Lai CC, Wu CH, Wang YH, Wang CY, Wu VC, Chen L. The association between COPD and outcomes of patients with advanced chronic kidney disease. *Int J Chron Obstruct Pulmon Dis*. 2018;13:2899-2905. doi:10.2147/COPD.S174215
154. Lange P, Colak Y, Ingebrigtsen TS, Vestbo J, Marott JL. Long-term prognosis of asthma, chronic obstructive pulmonary disease, and asthma-chronic obstructive pulmonary disease overlap in the Copenhagen City Heart study: a prospective population-based analysis. *Lancet Respir Med*. Jun 2016;4(6):454-62. doi:10.1016/S2213-2600(16)00098-9
155. Liapikou A, Polverino E, Ewig S, et al. Severity and outcomes of hospitalised community-acquired pneumonia in COPD patients. *Eur Respir J*. Apr 2012;39(4):855-61. doi:10.1183/09031936.00067111
156. Lim JU, Kang HS, Yeo CD, et al. Impact of Combined Chronic Obstructive Pulmonary Disease Status and Systemic Inflammation on Outcome of Advanced NSCLC: Multicenter Retrospective Cohort Study. *Int J Chron Obstruct Pulmon Dis*. 2020;15:3323-3334. doi:10.2147/COPD.S274354
157. Lindberg A, Larsson LG, Muellerova H, Ronmark E, Lundback B. Up-to-date on mortality in COPD - report from the OLIN COPD study. *BMC Pulm Med*. Jan 9 2012;12:1. doi:10.1186/1471-2466-12-1
158. Londono KL, Formiga F, Chivite D, Moreno-Gonzalez R, Migone De Amicis M, Corbella X. Prognostic influence of prior chronic obstructive pulmonary disease in patients admitted for their first episode of acute heart failure. *Intern Emerg Med*. Apr 2018;13(3):351-357. doi:10.1007/s11739-018-1820-3
159. Marott JL, Ingebrigtsen TS, Colak Y, Vestbo J, Lange P. Lung Function Trajectories Leading to Chronic Obstructive Pulmonary Disease as Predictors of Exacerbations and Mortality. *Am J Respir Crit Care Med*. Jul 15 2020;202(2):210-218. doi:10.1164/rccm.201911-2115OC
160. Mendez-Bailon M, Lopez-de-Andres A, de Miguel-Diez J, et al. Chronic obstructive pulmonary disease predicts higher incidence and in hospital mortality for atrial fibrillation. An observational study using hospital discharge data in Spain (2004-2013). *Int J Cardiol*. Jun 1 2017;236:209-215. doi:10.1016/j.ijcard.2017.02.017
161. Myagmardorj R, Nabeta T, Hirasawa K, et al. Association Between Chronic Obstructive Pulmonary Disease and All-Cause Mortality After Aortic Valve Replacement for Aortic Stenosis. *Am J Cardiol*. Mar 1 2023;190:41-47. doi:10.1016/j.amjcard.2022.11.007
162. Nogimura A, Noguchi T, Otani T, et al. Chronic obstructive pulmonary disease and the mortality risk in male older adults: Role of socioeconomic factors. *Arch Gerontol Geriatr*. Sep-Oct 2022;102:104741. doi:10.1016/j.archger.2022.104741
163. Opina MTD, Nicklas BJ, Lovato JF, Files DC, Kritchevsky SB, Moore WC. Association of Symptoms of Obstructive Lung Disease and All-Cause Mortality in Older Adult Smokers. *J Am Geriatr Soc*. Oct 2019;67(10):2116-2122. doi:10.1111/jgs.16052
164. Parissis JT, Andreoli C, Kadoglou N, et al. Differences in clinical characteristics, management and short-term outcome between acute heart failure patients chronic obstructive pulmonary disease and those without this co-morbidity. *Clin Res Cardiol*. Sep 2014;103(9):733-41. doi:10.1007/s00392-014-0708-0
165. Ponomarev D, Kamenskaya O, Klinkova A, et al. Chronic Lung Disease and Mortality after Cardiac Surgery: A Prospective Cohort Study. *J Cardiothorac Vasc Anesth*. Oct 2018;32(5):2241-2245. doi:10.1053/j.jvca.2017.12.016

166. Purdue MP, Gold L, Jarvholm B, Alavanja MC, Ward MH, Vermeulen R. Impaired lung function and lung cancer incidence in a cohort of Swedish construction workers. *Thorax*. Jan 2007;62(1):51-6. doi:10.1136/thx.2006.064196
167. Quan T, Chen FR, Recarey M, et al. Chronic obstructive pulmonary disease is an independent risk factor for postoperative complications following operative treatment of distal radius fracture. *Eur J Orthop Surg Traumatol*. Jul 2022;32(5):945-951. doi:10.1007/s00590-021-03067-0
168. Rello J, Rodriguez A, Torres A, et al. Implications of COPD in patients admitted to the intensive care unit by community-acquired pneumonia. *Eur Respir J*. Jun 2006;27(6):1210-6. doi:10.1183/09031936.06.00139305
169. Rodriguez-Manero M, Lopez-Pardo E, Cordero A, et al. A prospective study of the clinical outcomes and prognosis associated with comorbid COPD in the atrial fibrillation population. *Int J Chron Obstruct Pulmon Dis*. 2019;14:371-380. doi:10.2147/COPD.S174443
170. Romiti GF, Corica B, Mei DA, et al. Impact of chronic obstructive pulmonary disease in patients with atrial fibrillation: an analysis from the GLORIA-AF registry. *Europace*. Dec 28 2023;26(1)doi:10.1093/europace/euae021
171. Rothnie KJ, Smeeth L, Herrett E, et al. Closing the mortality gap after a myocardial infarction in people with and without chronic obstructive pulmonary disease. *Heart*. Jul 2015;101(14):1103-10. doi:10.1136/heartjnl-2014-307251
172. Salisbury AC, Reid KJ, Spertus JA. Impact of chronic obstructive pulmonary disease on post-myocardial infarction outcomes. *Am J Cardiol*. Mar 1 2007;99(5):636-41. doi:10.1016/j.amjcard.2006.09.112
173. Selvaraj CL, Gurm HS, Gupta R, Ellis SG, Bhatt DL. Chronic obstructive pulmonary disease as a predictor of mortality in patients undergoing percutaneous coronary intervention. *Am J Cardiol*. Sep 15 2005;96(6):756-9. doi:10.1016/j.amjcard.2005.05.016
174. Semenzato U, Biondini D, Bazzan E, et al. Low-Blood Lymphocyte Number and Lymphocyte Decline as Key Factors in COPD Outcomes: A Longitudinal Cohort Study. *Respiration*. 2021;100(7):618-630. doi:10.1159/000515180
175. Stone DH, Goodney PP, Kalish J, et al. Severity of chronic obstructive pulmonary disease is associated with adverse outcomes in patients undergoing elective abdominal aortic aneurysm repair. *J Vasc Surg*. Jun 2013;57(6):1531-6. doi:10.1016/j.jvs.2012.11.132
176. Su TH, Chang SH, Chen PC, Chan YL. Temporal Trends in Treatment and Outcomes of Acute Myocardial Infarction in Patients With Chronic Obstructive Pulmonary Disease: A Nationwide Population-Based Observational Study. *J Am Heart Assoc*. Mar 15 2017;6(3)doi:10.1161/JAHA.116.004525
177. Vaes AW, Garcia-Aymerich J, Marott JL, et al. Changes in physical activity and all-cause mortality in COPD. *Eur Respir J*. Nov 2014;44(5):1199-209. doi:10.1183/09031936.00023214
178. Wang R, Tomaniak M, Takahashi K, et al. Impact of chronic obstructive pulmonary disease on 10-year mortality after percutaneous coronary intervention and bypass surgery for complex coronary artery disease: insights from the SYNTAX Extended Survival study. *Clin Res Cardiol*. Jul 2021;110(7):1083-1095. doi:10.1007/s00392-021-01833-y
179. Wu PK, Ho CH, Wu YC, Liao KM, Chien CS. Impact of Chronic Obstructive Pulmonary Disease on Mortality Rates After Hip Pertrochanteric Fracture: A Nationwide Population-Based Cohort Study. *Int J Chron Obstruct Pulmon Dis*. 2025;20:4019-4029. doi:10.2147/COPD.S559900
180. Xiao F, Yang J, Fan R. Effects of COPD on in-hospital outcomes of transcatheter aortic valve implantation: Results from the National Inpatient Sample database. *Clin Cardiol*. Dec 2020;43(12):1524-1533. doi:10.1002/clc.23475
181. Zhang P, Ma Y, Chen X, et al. The Difference in All-Cause Mortality Between Allergic Bronchopulmonary Aspergillosis with and without Chronic Obstructive Pulmonary Disease. *J Asthma Allergy*. 2022;15:1861-1875. doi:10.2147/JAA.S389985

182. Zhang JQ, Cheng TM, Lin WC, Chiu KC, Wu SY. Impact of Smoking-Related Chronic Obstruction Pulmonary Disease on Mortality of Invasive Ductal Carcinoma Patients Receiving Standard Treatments: Propensity Score-Matched, Nationwide, Population-Based Cohort Study. *Cancers (Basel)*. Jul 21 2021;13(15)doi:10.3390/cancers13153654

183. Zhang J, Chiu KC, Lin WC, Wu SY. Survival Impact of Chronic Obstructive Pulmonary Disease or Acute Exacerbation on Patients with Rectal Adenocarcinoma Undergoing Curative Resection: A Propensity Score-Matched, Nationwide, Population-Based Cohort Study. *Cancers (Basel)*. Aug 22 2021;13(16)doi:10.3390/cancers13164221
